# Supplementary material for: Conserved chromatin and repetitive patterns reveal slow genome evolution in frogs
Source: Nat Commun. 2024 Jan 17;15:579. doi: 10.1038/s41467-023-43012-9 (PMC10794172; doi:10.1038/s41467-023-43012-9)
Supplement: Supplementary file 1 — Supplementary Information [file 41467_2023_43012_MOESM1_ESM.pdf]

Conserved chromatin and repetitive patterns reveal slow genome evolution in frogs

Jessen V. Bredeson, Austin B. Mudd, Sofia Medina-Ruiz, et al.

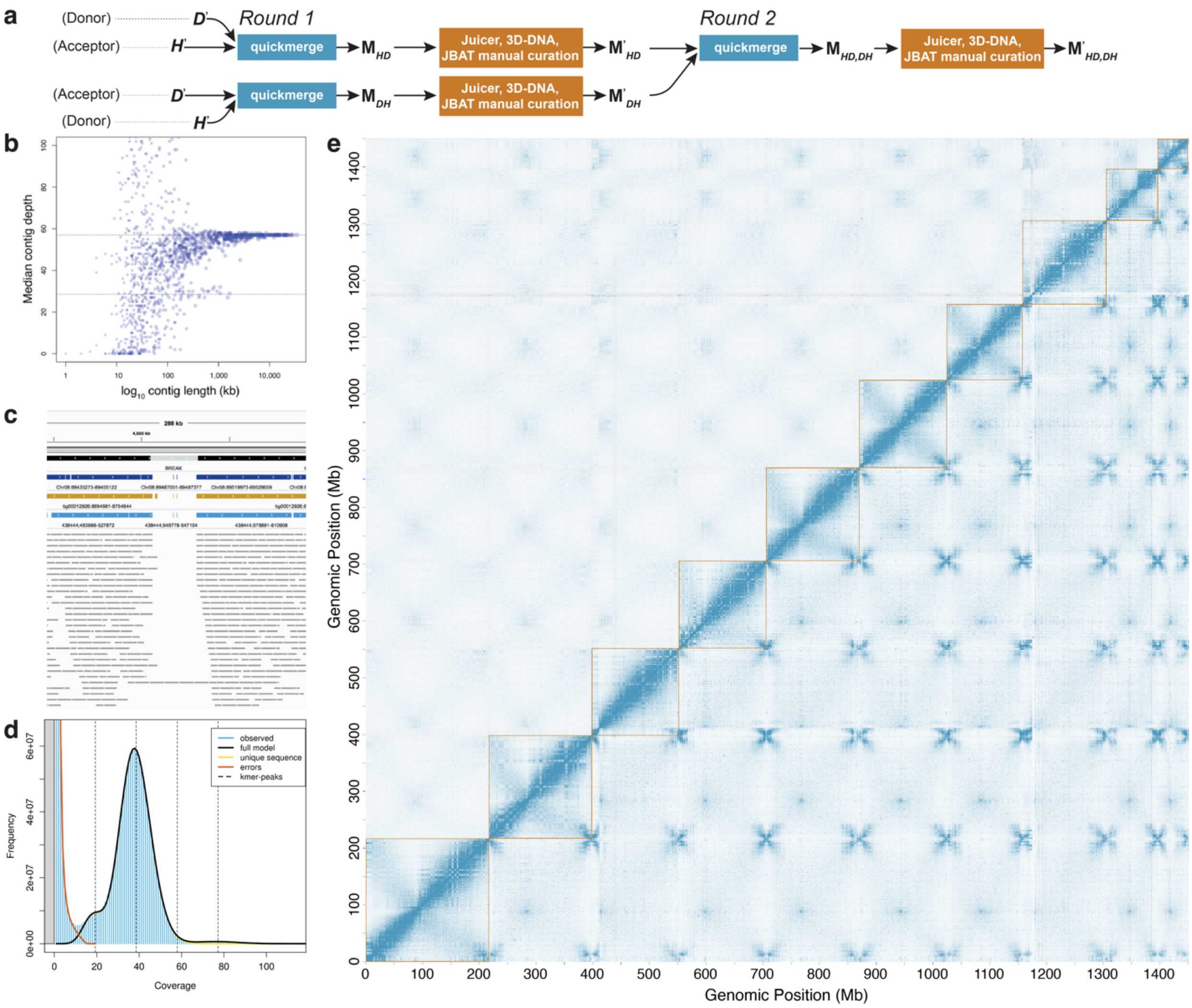

Supplementary Fig. 1 ***Xenopus tropicalis*** **genome assembly process and Hi-C contact map.**
(**a**) Round 1 of metassembly with quickmerge: *de novo* (*D*) and hybrid (*H*) contigs are used as both "donor" and "acceptor" sequences in a reciprocal merging strategy to produce two metassemblies, M_{HD} and M_{DH}, which are then corrected for mis-assemblies using Juicer and Juicebox Assembly Tools (JBAT) with manual curation. This results in two sets of corrected contigs, M'_{HD} and M'_{DH}. Round 2: the process is repeated with M'_{HD} and M'_{DH} to produce a final second-order metassembly, $M'_{HD,DH}$. (**b**) The median depth of coverage (Y-axis) was calculated for each contig (blue dots) and plotted against its $\log_{10}$-transformed length (X-axis) to identify redundant sequences. The plot shows that contig sequences can be stratified into longer full-depth (y = 58×) and shorter half-depth (y = 29×) categories. (**c**) Black horizontal bars are regions of the genome well-supported by spanning PacBio read alignments (thin grey horizontal bars). The light grey region labeled "BREAK" lacks support by read data and represents a DBG2OLC assembly error likely introduced by incorporating a single PacBio polymerase read that was not successfully broken into individual subreads. Dark blue horizontal bars represent the aligned Sanger-based v9 assembly, while gold and light blue horizontal bars represent the raw Canu and Supernova contigs underlying the hybrid assembly. Note that both these sequences flank the BREAK region, meaning these contigs span the artefact and can be used to patch it. (**d**) GenomeScope model fit and genome-size estimate for *X. tropicalis* Nigerian F_{17} female. Observed k-mer frequency per depth-of-coverage bin as blue vertical lines; error model curve fit to k-mer frequencies resulting from sequencing errors in brown; model fit curve to k-mer frequencies generated from unique genomic sequence in yellow; combined unique and error model fit curve in solid black; vertical dashed lines represent one-, two-, three-, and four-copy sequence depth peaks, respectively. (**e**) Hi-C contact matrix from red blood nuclei at 500 kb resolution, balanced using the Knight-Ruiz algorithm, showing reads with a minimum mapping quality (MQ) ≥ 0 below the diagonal and reads with MQ ≥ 30 above the diagonal. Chromosomes (gold boxes) are shown in ascending numeric order along the X-axis, with p-arms oriented toward the lower left of the figure and q-arms toward the upper right. The intensity of blue pixels is proportional to chromatin contact frequencies between X-Y pairs of 500 kb genomic loci. Intra-chromosomal contacts exhibit the highest frequency of contacts between adjacent loci along the linear chromosome (along the diagonal). Above the diagonal, inter-chromosomal contacts are the strongest between centromeres (puncta), while below the diagonal the strongest contacts are observed between subtelomeric sequences. This contact density map was visualized with Juicebox[1,2]. kb, kilobases; Mb, megabases. Source data are provided as a Source Data file.

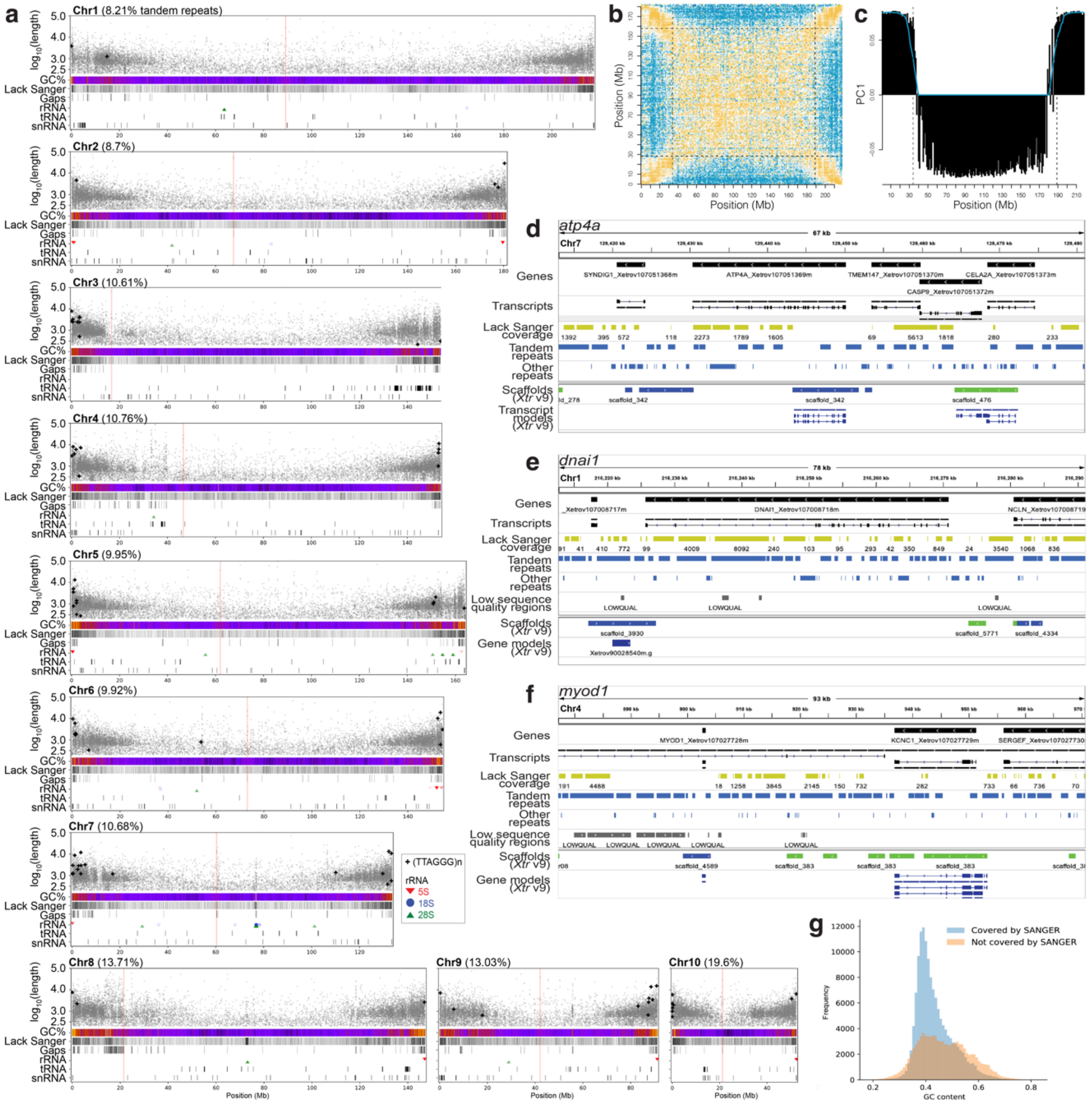

Supplementary Fig. 2 ***Xenopus tropicalis*** **GC landscape, tandem repeats, and recovered genes.**
(**a**) Long arrays of tandem repeats localize to subtelomeric portions of the genome. A total of 68.27 Mb (4.71%) of the *X. tropicalis* (*Xtr*) genome assembly was not covered by Sanger shotgun sequences. Of the regions lacking Sanger read alignments, 48.6% overlapped with tandem repeats and 34.77% with other types of repetitive elements. Of the 4,718 CDS not supported by Sanger reads, 3,511 (74.41%) of them are localized in close proximity (< 2 kb) to tandem repeats. GC% content in distal subtelomeric regions is elevated, and regions lacking Sanger read coverage are more prevalent in the subtelomeres (48.6% of these regions overlap with tandem repeats). Telomere-associated tandem repeats, (TTAGGG)n, are indicated with plus ("+") symbols. Gaps in the current v10 reference genome assembly tend to co-localize with long tandem arrays. The locations of clusters of 5S (green), 18S (blue), and 28S (green) rRNAs, tRNAs, and snRNAs are indicated. Red vertical lines indicate the position of centromeres. (**b**) An example of subtelomere boundary inference for chromosome 1 (vs. chromosome 2) using feature selection with k-means clustering on a matrix selecting for repetitive Hi-C read placements in the subtelomeres (yellow, high-density corners), depletion of subtelomere-specific signal with the rest of the chromosome (blue edges), and background/aspecific repetitive signal (center). Dashed horizontal and vertical lines demarcate the inferred subtelomere boundaries. (**c**) Principal component analysis of the chromosome 1 matrix in panel **b**, decomposing variances into subtelomere-specific (positive Y values) and subtelomere-depleted repeat signals (negative Y values). Dashed vertical lines demarcate the inferred subtelomere boundaries. (**d–f**) IGV[3] (v2.7.2) views from two fully-assembled loci in the current *X. tropicalis* (v10) assembly previously fragmented in the v9 assembly due to lack of Sanger read coverage. (**d**) *atp4a* (*Xetrov107051369m*; Chr7:129,430,384–129,450,182) was partially assembled in v9. Note that most regions not covered by Sanger (yellow) overlap repeats (blue). (**e**) *dnai1* (*Xetrov107008718m*; Chr1:216,212,747–216,291,747) was completely missing from v9 and is now captured in the v10 assembly. (**f**) Due to the highly repetitive nature of the sequence surrounding the *myod1* locus (*Xetrov107027728m*; Chr4:719,782–1,128,311) this gene remains fragmented in the v10 assembly. The number below the “Lack Sanger coverage” track corresponds to the size of the fragment not covered by reads.
(**g**) GC% distribution of genomic regions greater than 100 bp that were covered (n = 140,598) or not-covered (n = 95,475) by Sanger reads. kb, kilobases; Mb, megabases. Source data are provided as a Source Data file.

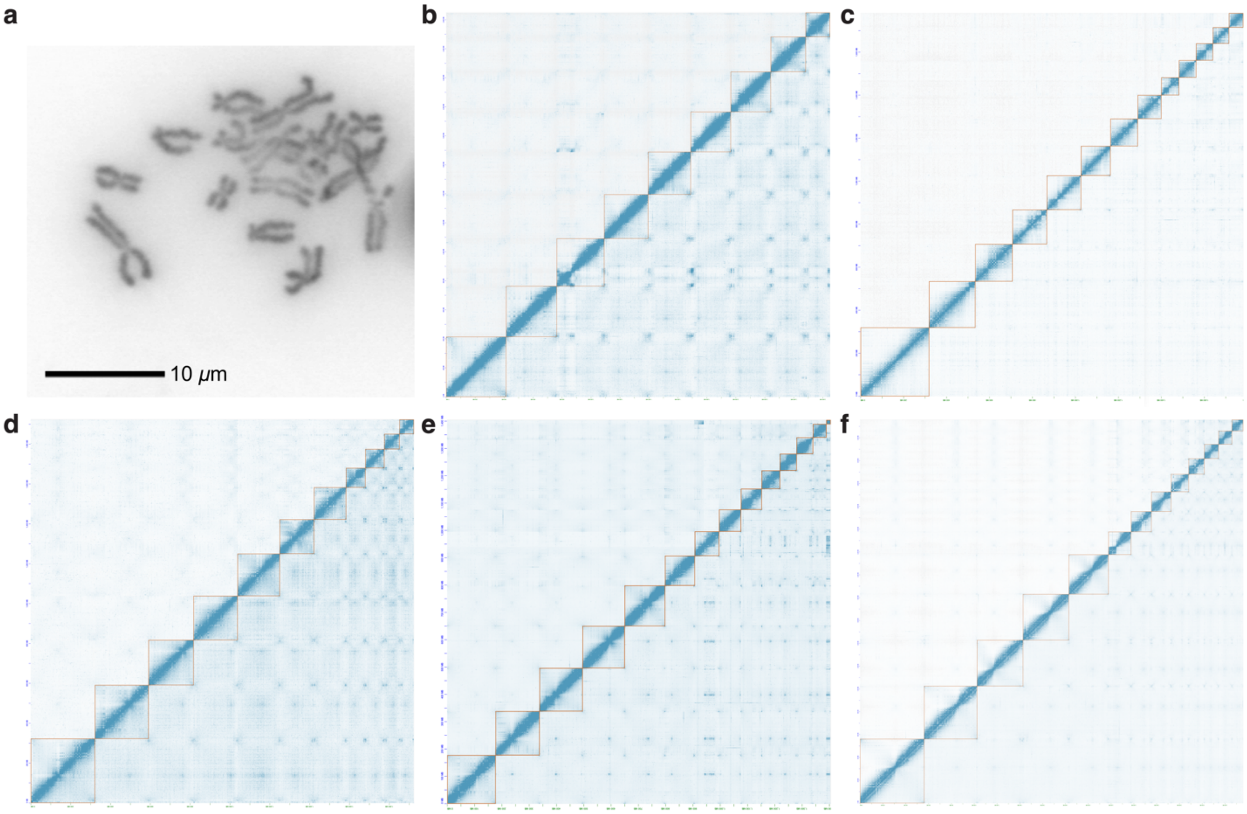

Supplementary Fig. 3 Assembly of five additional frog species.
(**a**) A representative *Hymenochirus boettgeri* metaphase chromosome spread selected from among n = 75 independent cell spreads prepared from ten individual *H. boettgeri* tadpoles. Nine chromosome pairs can be observed for a karyotype of 2n = 18 (mode = 18, mean ± SEM = 18.09 ± 0.22). The scale bar represents 10 □m. Panels **b–f** present whole-genome Juicebox[1,2] visualizations of Hi-C contact maps from (**b**) *H. boettgeri*, (**c**) *Eleutherodactylus coqui,* (**d**) *Engystomops pustulosus*, (**e**) *Pyxicephalus adspersus,* and (**f**) *Leptobrachium* (*Vibrissaphora*) *ailaonicum* chromosomes. Hi-C contact matrices balanced using the Knight-Ruiz algorithm, showing reads with a minimum mapping quality (MQ) ≥ 0 below the diagonal and reads with MQ ≥ 30 above the diagonal. Chromosomes (gold boxes) are shown in ascending numeric order along the X-axis and the intensity of blue pixels is proportional to chromatin contact frequencies between X-Y pairs of non-overlapping genomic loci. Source data are provided as a Source Data file.

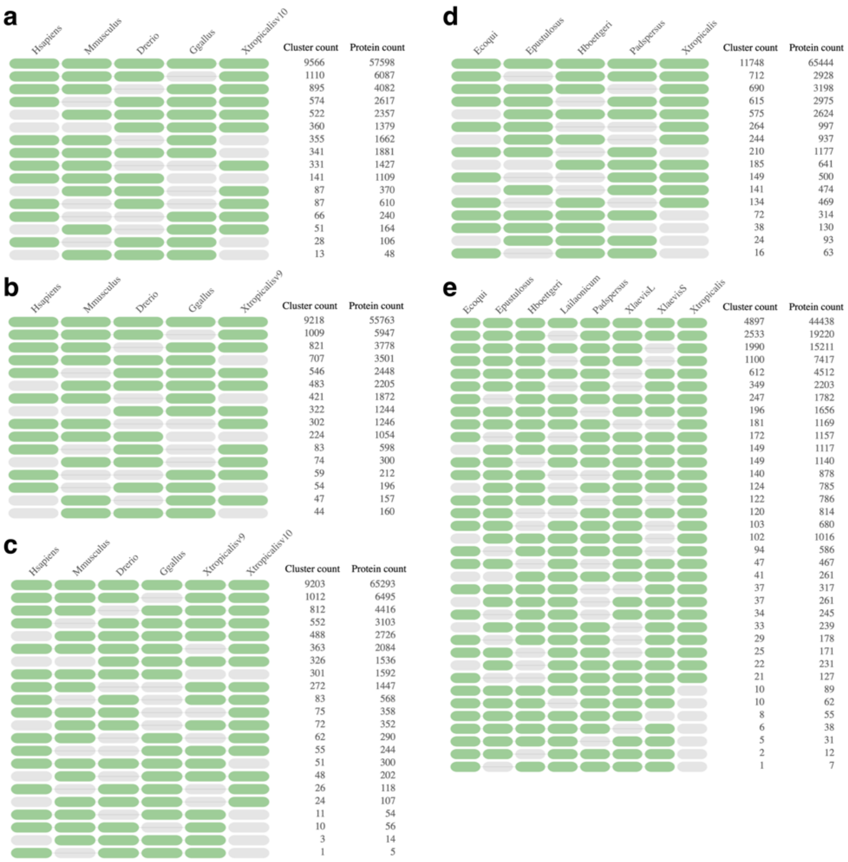

Supplementary Fig. 4 Comparison of gene content among assemblies.
In the above occurrence tables, green and grey represent the presence or absence, respectively, of a species in a gene family cluster. The cluster count column summarizes the number of such gene family clusters with that occurrence pattern, while the protein count column lists the number of genes included in those gene clusters. Tables (**a**) containing three or more species with the v10 annotation primary transcripts, (**b**) containing three or more species with the v9 annotation longest transcripts[4] (NCBI Annotation Release 103 of RefSeq assembly accession GCF_000004195.3 [https://www.ncbi.nlm.nih.gov/datasets/genome/GCF_000004195.3]), and (**c**) containing four or more species with both v9 and v10 against the longest transcripts of *Danio rerio* (NCBI Annotation Release 106 of RefSeq assembly accession GCF_000002035.6 [https://www.ncbi.nlm.nih.gov/datasets/genome/GCF_000002035.6]), *Gallus gallus* (NCBI Annotation Release 104 of RefSeq assembly accession GCF_000002315.6 [https://www.ncbi.nlm.nih.gov/datasets/genome/GCF_000002315.6]) *Homo sapiens* (NCBI Annotation Release 109 of RefSeq assembly accession GCF_000001405.39 [https://www.ncbi.nlm.nih.gov/datasets/genome/GCF_000001405.39]), and *Mus musculus* (NCBI Annotation Release 108 of RefSeq assembly accession GCF_000001635.26 [https://www.ncbi.nlm.nih.gov/datasets/genome/GCF_000001635.26]). Occurrence tables of gene homology clusters containing (**d**) three or more species among the five frog annotations newly generated for this study and (**e**) six or more members among the full set of seven frogs and—with both *X. laevis* L and S homoeolog sets represented—eight (sub)genomes analyzed here. All OrthoVenn2[5] clustering analyses were completed with the longest transcript amino acid sequences extracted using gff3ToGenePred and genePredToProt (UCSC Genomics Institute[6] KentTools binaries downloaded March 5, 2019) as well as custom script largestgenePred.py (v1.0, https://github.com/abmudd/Assembly). Source data are provided as a Source Data file.

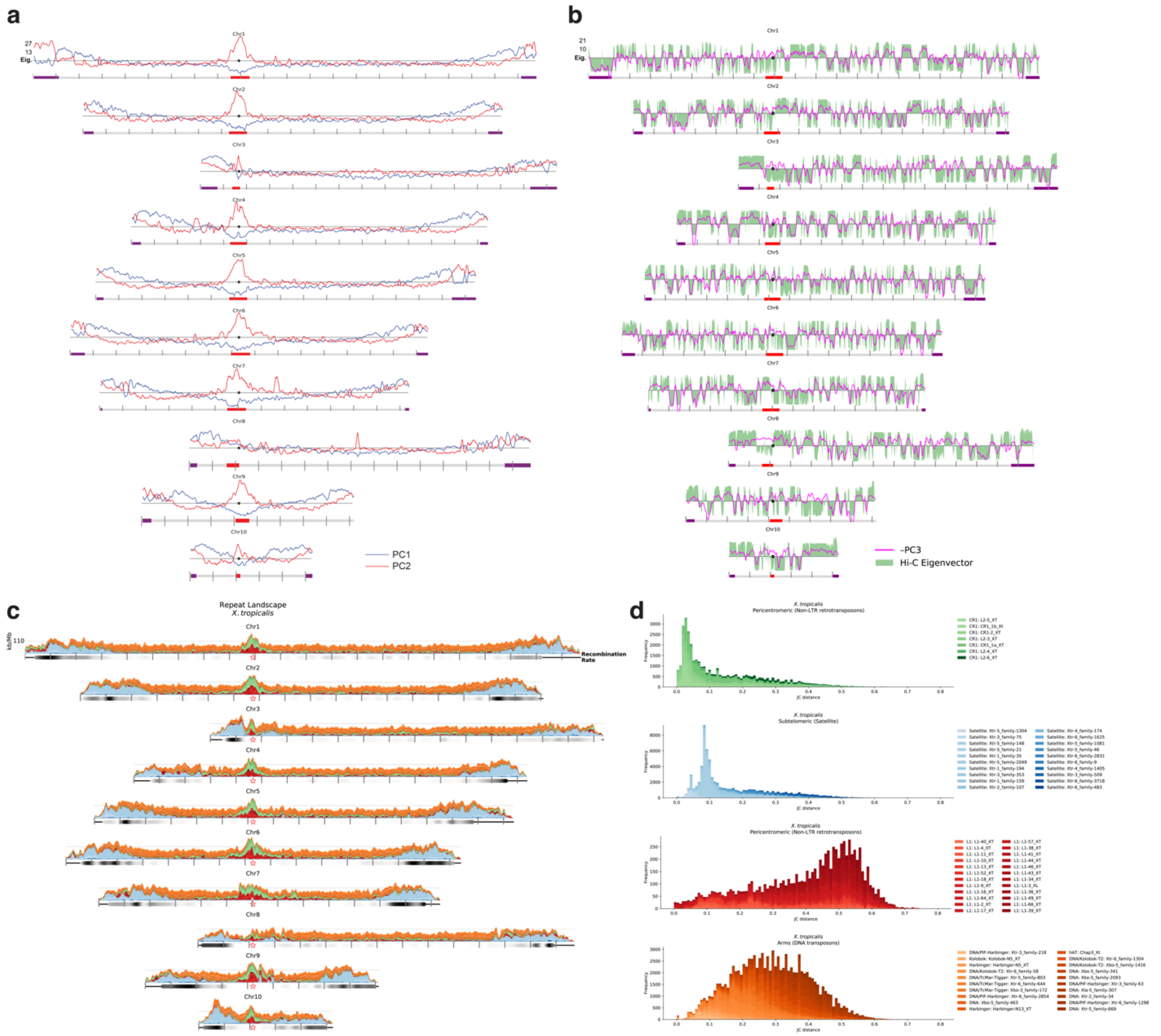

Supplementary Fig. 5 ***Xenopus tropicalis*** **repeat density principal components projected on genomic coordinates.** (**a**) The first two principal components (PCs) of the repeat density matrix describe subtelomeric and pericentromeric regions of the chromosomes. The first component (PC1, blue lines) strongly correlates with GC content (Pearson's r = +0.796). The grey horizontal line is the value for which the smoothed PC2 (red lines) was thresholded to discriminate between distal-subtelomeric (purple boxes), pericentromeric (red boxes), and arm regions (grey) of each chromosome. Chromosomes are centered by the median position of centromere-associated tandem repeats (black dots). Tick lines correspond to distances of 10 Mb. (**b**) Plot as in panel **a** showing the third principal component (PC3, purple lines) of the repeat matrix plotted with the eigenvector that defines A/B compartment structure (green; A and B compartments above and below the X-axis, respectively) obtained from the Hi-C contact matrix. PC3 and the Hi-C-derived eigenvector show a moderately negative correlation (Pearson's r = −0.64) when analyzed in 500 kb windows along the genome. This negative correlation with PC3 is maintained, although weaker, when the analysis is repeated with consecutive Hi-C windows with equivalent signs collapsed into larger A/B compartment domain block intervals (Pearson's r = −0.44). Harbinger-N9_XT is positively associated with PC3 (Pearson's r = +0.50), whereas DNA/hAT-Ac appears strongly negatively correlated with PC3 (Pearson's r = −0.80). (**c**) Repeat landscape of L1 (red) and CR1 (green) Non-LTR retrotransposons, satellite repeats (blue), and DNA transposons (orange). Recombination rate plotted along the X-axis as a density gradient track (black = high, white = low). Centromere positions are represented with red stars. (**d**) Jukes-Cantor (JC) distance (X-axis) from the consensus sequence for the repeats shown in panel **a**. Only the most abundant L1 repeat families are shown from panel **b**. kb, kilobases; Mb, megabases. Source data are provided as a Source Data file.

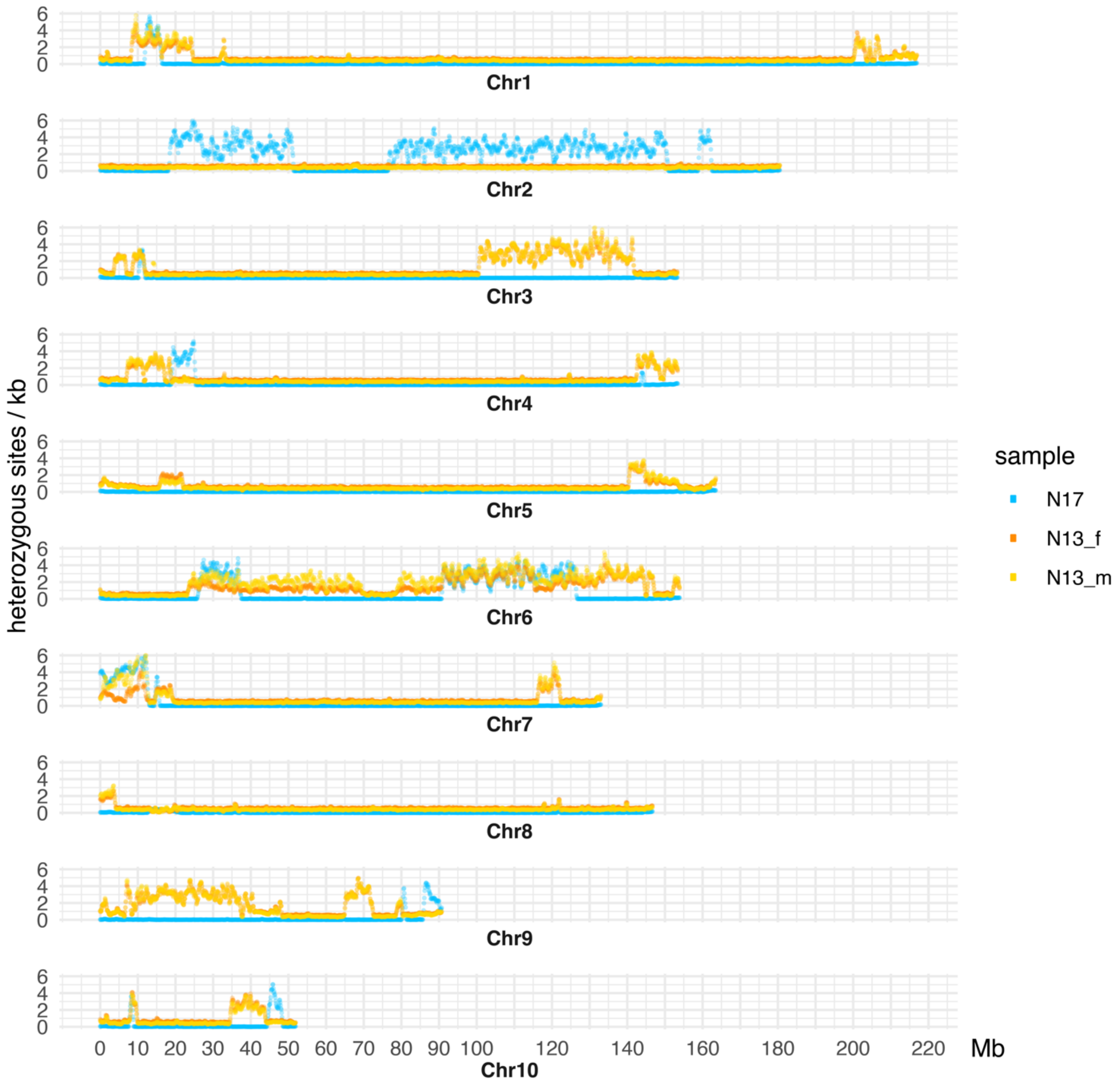

Supplementary Fig. 6 ***Xenopus tropicalis*** **Nigerian strain residual heterozygosity.**
Heterozygosity rates for the N17 genome frog (an F_{17} inbred line; blue) and two pooled F_{13} inbred Nigerian strain datasets: N13_f, eight Nigerian females (orange); and N13_m, twelve Nigerian males (yellow). Rates are measured as heterozygous SNP positions per kilobase. kb, kilobases; Mb, megabases; SNP, single-nucleotide polymorphism. Source data are provided as a Source Data file.

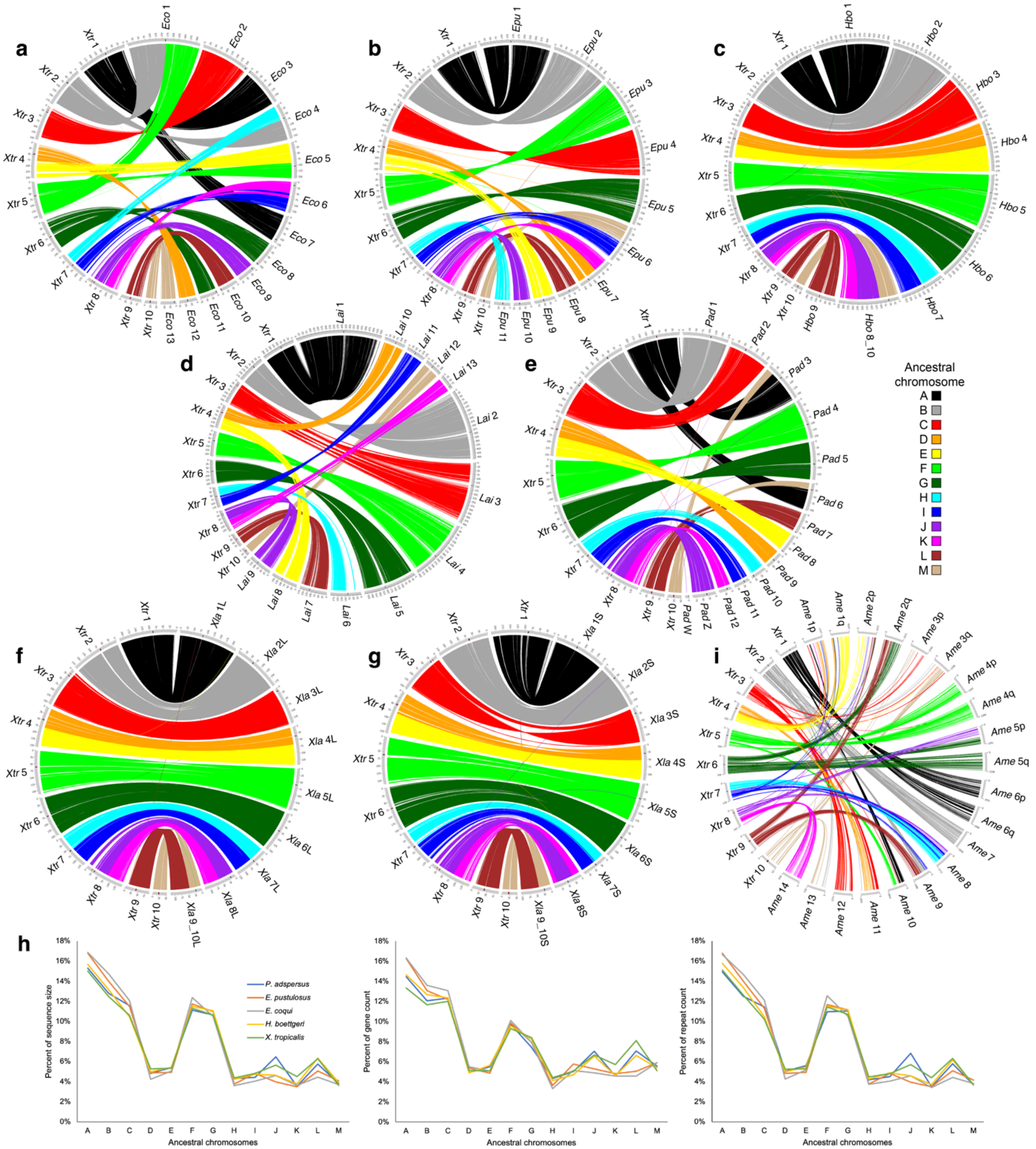

Supplementary Fig. 7 ***Xenopus tropicalis*** **gene collinearity with the genomes of six frogs and axolotl.**
Circos plots with runs of collinearity containing at least 5 kb of aligned sequence between *Xenopus tropicalis* (left, *Xtr*) and (**a**) *Eleutherodactylus coqui* (right, *Eco*), (**b**) *Engystomops pustulosus* (right, *Epu*), (**c**) *Hymenochirus boettgeri* (right, *Hbo*), (**d**) *Leptobrachium* (*Vibrissaphora*) *ailaonicum* (right, *Lai*), (**e**) *Pyxicephalus adspersus* (right, *Pad*), (**f**) *X. laevis* L subgenome (right, *Xla* L), and (**g**) *X. laevis* S subgenome (right, *Xla* S). Chromosomes are represented by light grey bars, Hi-C-based and tandem repeat centromere positions are marked with black and red intersecting lines, respectively. Minor and major ticks delimit 5 Mb and 25 Mb intervals, respectively. (**h**) Percent of total chromosome sequence size for each ancestral unit, gene density in genes per 100 kb for each ancestral unit, and repeat density in repeats per 1 kb for each ancestral unit. Boundaries of the ancestral units were extracted from runs of collinearity containing at least 1 kb of sequence aligned against *L. ailaonicum*. Circos plots with runs of collinearity, as in panels **a**–**g**, for (**i**) *Ambystoma mexicanum* (right, *Ame*); *X. tropicalis* and *A. mexicanum* chromosomes are scaled evenly. Runs of collinearity are colored with respect to the 13 ancestral chromosomes (A: black, B: grey, C: red, D: orange, E: yellow, F: bright green, G: dark green, H: cyan, I: blue, J: purple, K: magenta, L: dark brown, and M: tan). kb, kilobases; Mb, megabases. Source data are provided as a Source Data file.

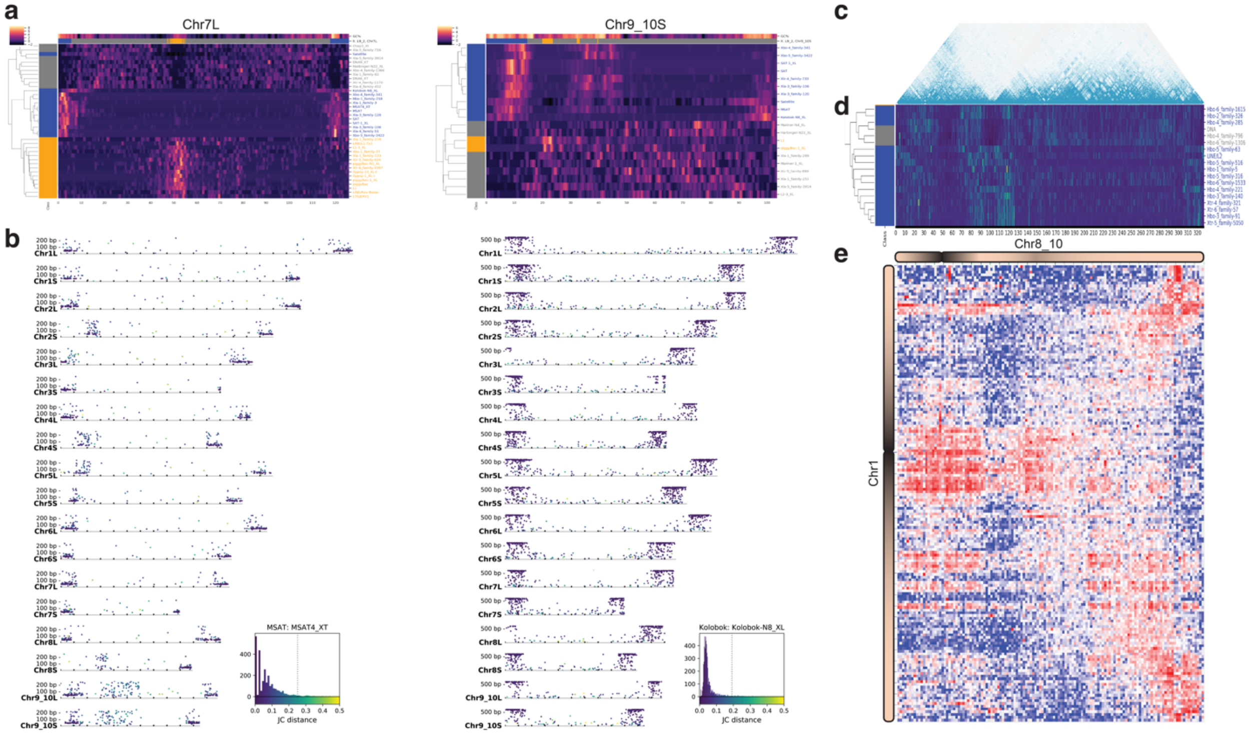

Supplementary Fig. 8 Chromosome fusions in *Xenopus laevis* and *Hymenochirus boettgeri*.
(**a**) Heatmap of repeat density in *X. laevis* showing repeats enriched in subtelomeric (blue tracks), pericentromeric (orange tracks), and arm regions (grey tracks) for chromosome 7L and the fused chromosome 9_10S. Three bands of subtelomeric signal can be detected on chromosome 9_10S, the signal between 30 and 50 Mb corresponds to the fusion of the subtelomeres from ancestral pipid chromosomes 9 and 10. (**b**) Scatterplots of two subtelomeric repeats showing, for each *X. laevis* chromosome (*X*-axis), the length (*Y*-axis) and Jukes-Cantor (JC) distance of the repeats colored as indicated by the histogram on the bottom right. The dotted vertical line on the histogram indicates the 95th percentile. The median JC distances from subtelomeres from all chromosomes (JC = 0.054) is lower than the median JC distances from the region of the chromosome 9_10 fusion (JC = 0.157), and for the relatively recent p-arm inversion on chromosomes 8S (JC = 0.099) and 2S (JC = 0.076), suggesting that the p-arm inversions from chromosomes 8S and 2S[7] occurred after the divergence from the pipid ancestor but after the fusion of chromosome 9_10S. Kolobok-N8_XL, a more recent repeat that expanded post-chromosome fusion and inversion of the p-arm of chromosomes 9S and 2S. (**c**) Hi-C contacts from chromosome 8_10 of *H. boettgeri* shows conserved intra-chromosomal contact boundaries compared to ancestral chromosomes 8 and 10. (**d**) Heatmap showing the density of subtelomeric repeats in *H. boettgeri* chromosome 8_10, between 85 and 120 Mb, from the fusion of ancestral chromosomes 8 and 10. (**e**) Enriched centromere-centromere contacts between *H. boettgeri* chromosome 1 (*Y*-axis) and chromosome 8_10 (*X*-axis), with the strongest centromeric interactions at x, y = 55 Mb, 170 Mb; suggesting that the active centromere of chromosome 8_10 was inherited from ancestral chromosome 10. A schematic of each chromosome, with pericentromeres colored black, are drawn along the axes. Enriched contacts shown in red, depletion in blue, and parity in white. Contacts enriched in the upper- and lower-right corners represent subtelomere-subtelomere contacts between the two chromosomes. Matrix of observed counts divided by expected counts, at 2.5 Mb matrix resolution (mapping quality ≥ 30, Knight-Ruiz balanced). bp, basepairs; Mb, megabases. Source data are provided as a Source Data file.

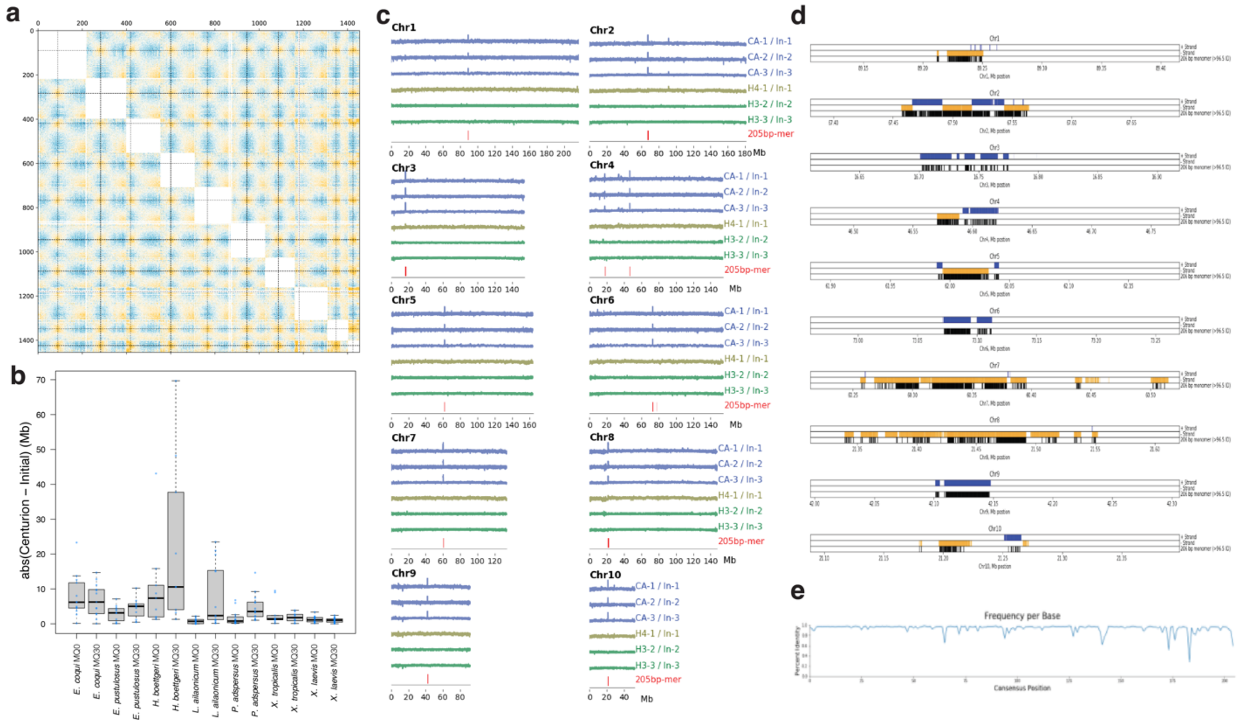

Supplementary Fig. 9 Estimating the positions of *Xenopus tropicalis* centromeres.
(**a**) Genome-wide Hi-C contact map of *X. tropicalis*. Intersecting horizontal and vertical dashed black lines mark the Centurion-estimated centromeres. Yellow puncta reflect centromere-centromere contact enrichment between chromosomes, suggesting centromere clustering on the inner nuclear periphery. Yellow, white, and blue colors indicate the normalized relative enrichment, parity, and depletion (respectively) between a pair of 1 Mb genomic loci. Intra-chromosomal contacts have been masked (solid white boxes). Axis coordinates in Mb. (**b**) Box plots comparing the variation in differences between manual and Centurion centromere estimates by mapping quality (MQ) for thresholds MQ ≥ 0 (MQ0) and MQ ≥ 30 (MQ30), where each blue point represents an estimate for a single chromosome. For each boxplot, n = the haploid number of chromosomes for the species-MQ combination indicated (X-axis). Lower and upper box edges denote the first and third quartiles, respectively, and whiskers extend to 1.5 times the interquartile range, while thick center lines represent medians. (**c**) ChIP-seq signal for Cenp-a, Histones H4 and H3. Samples are normalized by the read depth of the DNA associated with free-mononucleosomes (input). The presence of 205 bp tandem monomer sequences associated with Centromeric Tandem Repeats is indicated in red. (**d**) Zoomed-in view showing the organization of tandem centromeric repeat sequences (monomer = 205 bp) for each chromosome. A 300 kb window surrounding the presumptive centromeric repeats. Monomers found in the forward and reverse strands are shown in blue and yellow, respectively. Monomers sharing above 95% identity to the repeat consensus are shown in black. (**e**) The percentage of the major base per position of the multiple alignment of 3,693 genomic BLASTN hits from the initial 205 bp monomer sequence. To generate the 205 bp consensus we considered the most represented base per site (positions where alignments were over 50% gaps were omitted). bp, basepairs; kb, kilobases; Mb, megabases. Source data are provided as a Source Data file.

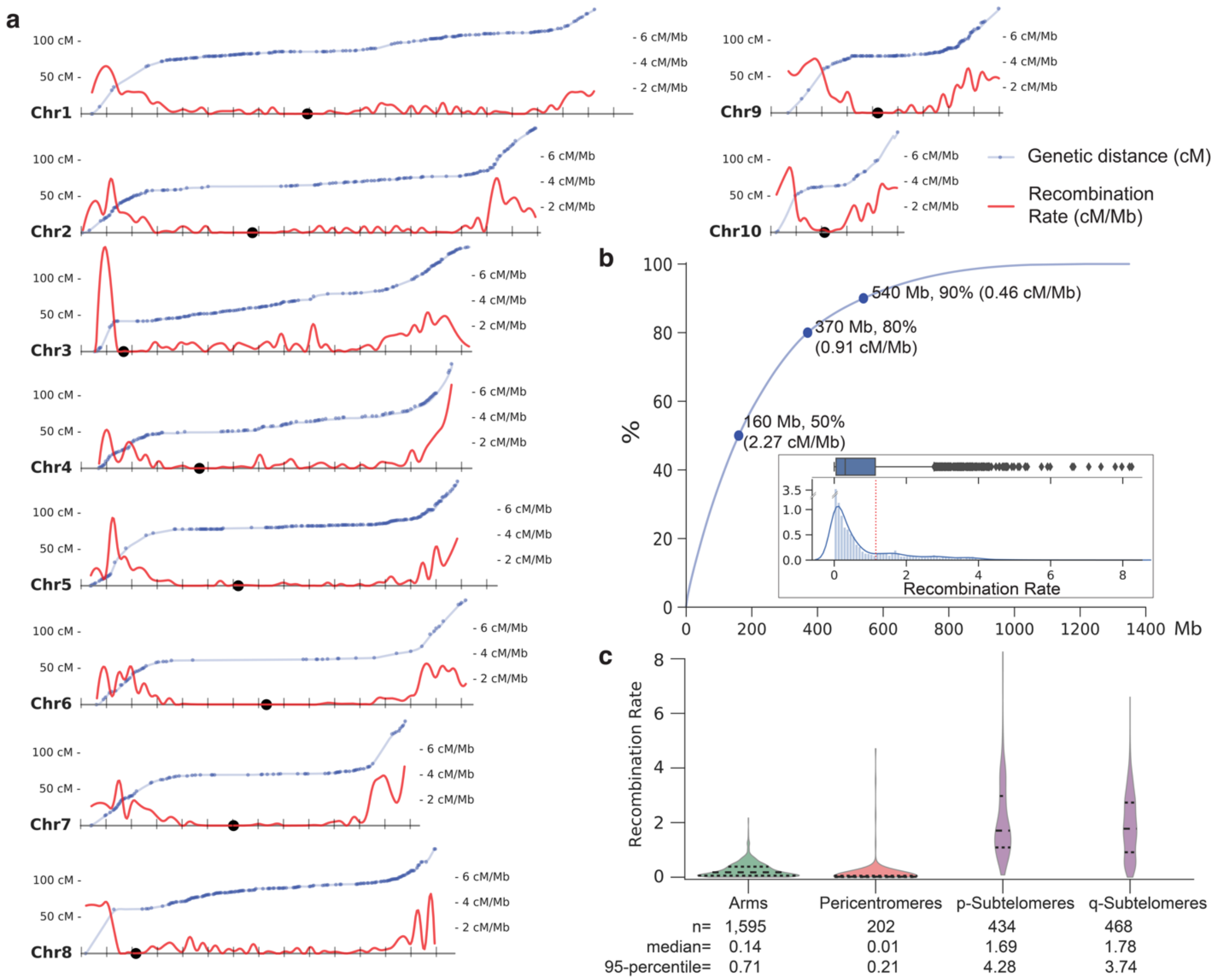

Supplementary Fig. 10 *Xenopus tropicalis* recombination landscape.
(**a**) Scatterplot of physical (X-axis) vs. genetic (Y-axis) position of the genetic map based on the *X. tropicalis* v10 genome assembly. Genetic map distances (blue dots) smoothened by cubic spline linear interpolation (blue line). Recombination rates (cM/Mb, red line) were obtained from the smoothened genetic distances. (**b**) Cumulative distribution of recombination rates. The distribution of recombination rates (lower histogram) was used to determine genetic positions with high and low recombination (75th percentile, red dotted line). Boxplot whiskers represent 1.5 times the interquartile range, the left and right boundaries of the box bound the first and third quartiles, respectively, and the center line marks the median. (**c**) Recombination rate (cM/Mb) distribution per chromosomal segment: 5 Mb surrounding centromere (pericentromeres), 30 Mb from the end of chromosme arms (p- and q-subtelomeres, excluding pericentromeres of acrocentric chromosomes), and the remaining chromosome arms. Significant differences are observed between distributions of the arms vs. subtelomeres (Hochberg-corrected $p = 5.2\times10^{-321}$) and the arms vs. pericentromeres (Hochberg-corrected $p = 3.2\times10^{-42}$) using two-sample Kolmogorov–Smirnov two-sided tests. Mb, megabases; cM, centiMorgans. Source data are provided as a Source Data file.

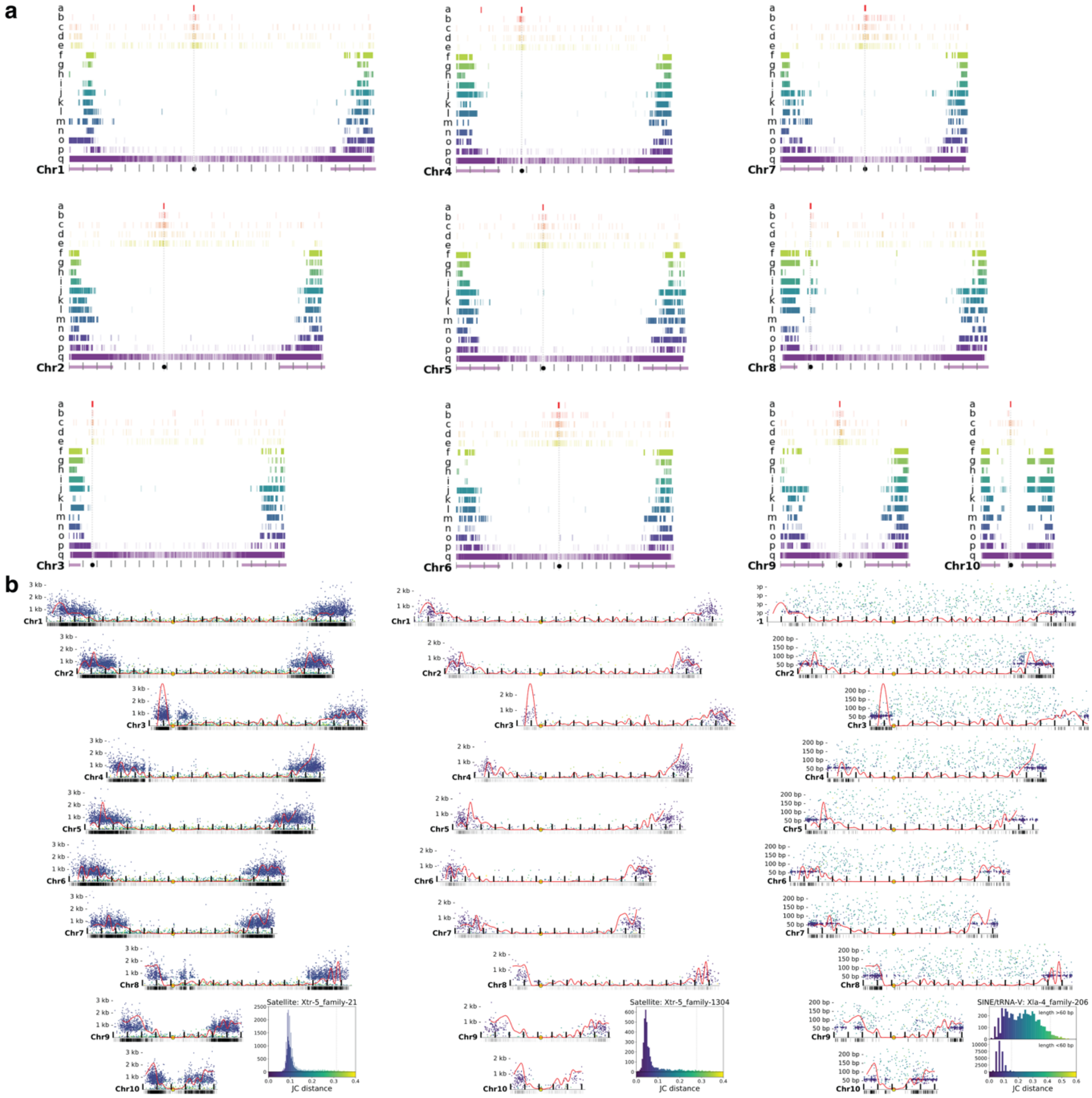

Supplementary Fig. 11 Distributions of satellite repeats in *Xenopus tropicalis*.
(**a**) Distribution of tandem repeats enriched in pericentromeric and subtelomeric regions. Tandem repeat monomers are overrepresented in pericentromeric (tracks *a*–*e*; red and oranges) and subtelomeric (tracks *f*–*q*; yellow–purple) regions. Each tick on the graph represents the alignment of a consensus monomer sequence sharing >90% of sequence identity. Sequence track *a* (red), 205-bp monomer, is the only monomer exclusive to the pericentromeric regions and is referred to as the Centromeric Tandem Repeat (CTR). The median position of CTRs per chromosome is indicated by the vertical dotted line and the black circle. The dashed vertical line indicates the estimated centromeric position from Hi-C using stringent mapping parameters. The purple horizontal lines correspond to 30 Mb spanning the subtelomeric domains. Monomer sequences best hit aligned against the repeat database can be found in **Supplementary Table 16**. (**b**) Scatter plot representing repeat length (Y-axis) and sequence divergence (Jukes-Cantor distance, color scheme) from satellite families: Xtr-5_family-21 (track *q* in panel **a**), Xtr-5_family-1304, and a SINE-V/tRNA: Xla-4_family-206 (track *p* in panel **a**). Long satellite repeats with low sequence divergence localize at subtelomeric portions overlapping areas of high recombination rates (red line). The bottom ticks indicate the presence of the monomer unit of the satellite repeats. The complete sequence of SINE-V/tRNA is relatively uniformly distributed in chromosome arms, except near the pericentromeres and subtelomeres. A minisatellite originated from a portion of the SINE-V/tRNA. Red curves plot the recombination rate profile in cM/Mb. The histogram of JC distances from SINE/tRNA-V sequences is subdivided by sequence length. Note how centromere-associated repeats preclude subtelomeric repeats in acrocentric chromosomes 3, 8, and 10. bp, basepairs; kb, kilobases; Mb, megabases; cM, centiMorgans. Source data are provided as a Source Data file.

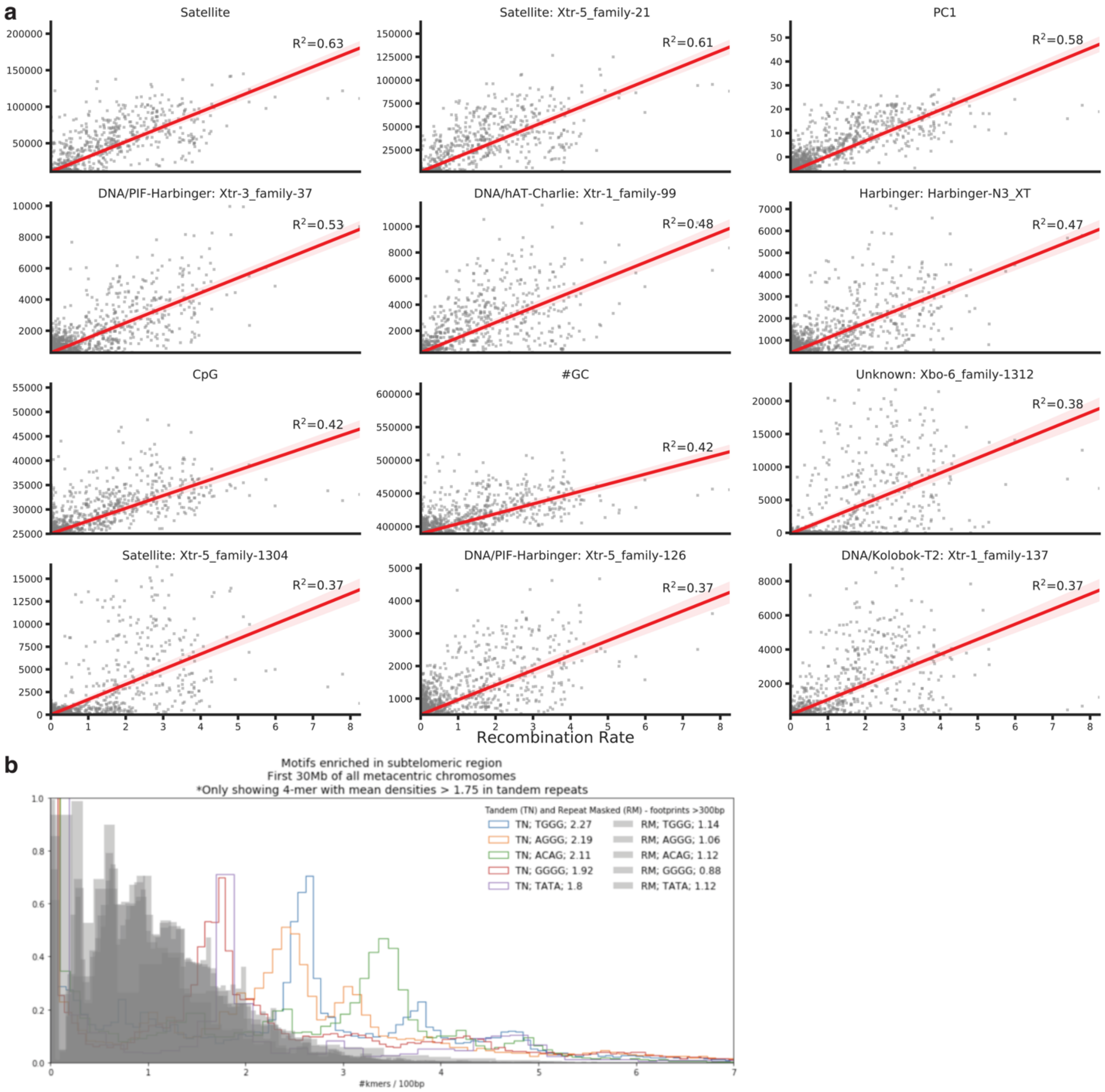

Supplementary Fig. 12 Correlates of recombination rate in *Xenopus tropicalis*.
(**a**) Scatterplots comparing the genome-wide recombination rates (X-axis) against the 12 highest correlated genomic features. PC1 corresponds to the first principal component (PC) obtained from repeat densities. Y-axis is the number of bases per 1-Mb size bins for regions with available genetic markers. A table of all genomic features correlated with recombination rate can be found in **Supplementary Table 14**. Each red line is the linear regression model for the plotted data points, while each semi-transparent red band highlights the 95% confidence interval of the linear model. (**b**) Enriched tetramers in subtelomeric sequences. The distribution of tetramers in genomic regions (> 300 bp) overlapping tandem repeats and other non-tandem repeats (grey) in the distal subtelomeric portions of (sub)metacentric chromosomes. The two most enriched monomers (AGGG/CCCT and TGGG/CCCA) are similar to the 7-nucleotide oligomer CCTCCCT and CCCACCCC that have been associated with recombination hotspots in human[8] and in mouse[9]. bp, basepairs; Mb, megabases. Source data are provided as a Source Data file.

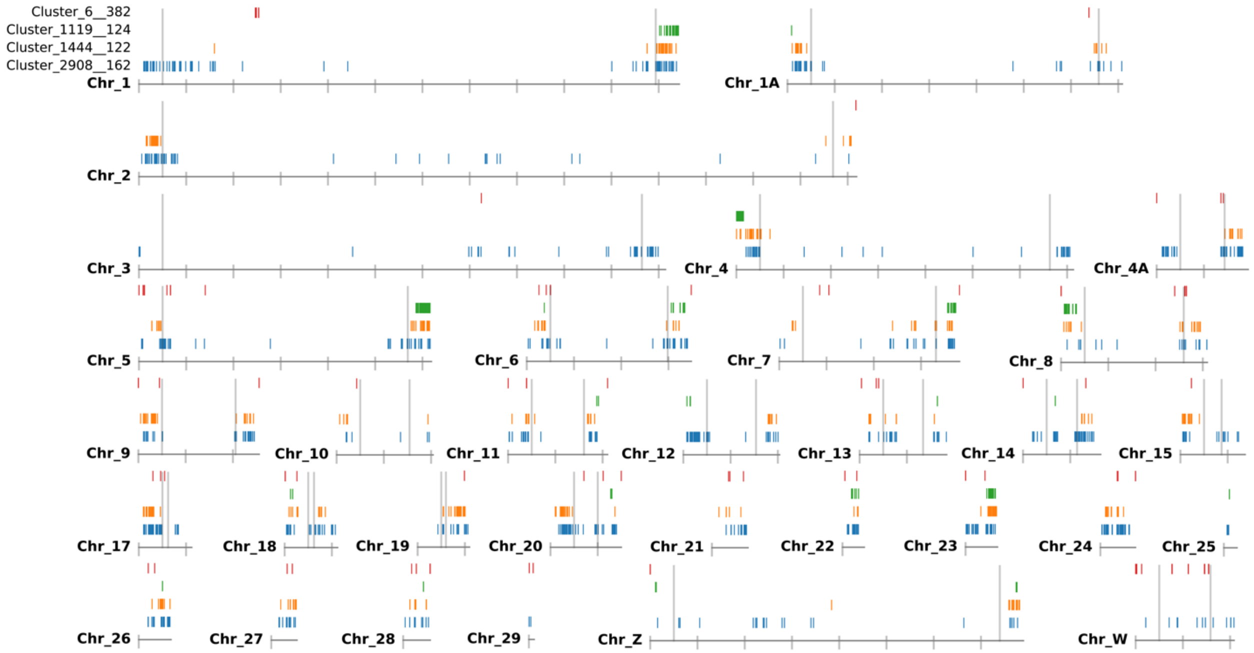

Supplementary Fig. 13 Zebra finch subtelomeric tandem repeats.
Genomic distributions of tandem repeats enriched in the subtelomeric portions of chromosomes (larger than 20 Mb in size). Grey vertical lines demarcate the first and last 5 Mb, where recombination rates are highest[10]. The tandem repeat sequences appear in at least four of the larger chromosomes and in most microchromosomes. Mb, megabases. Source data are provided as a Source Data file.

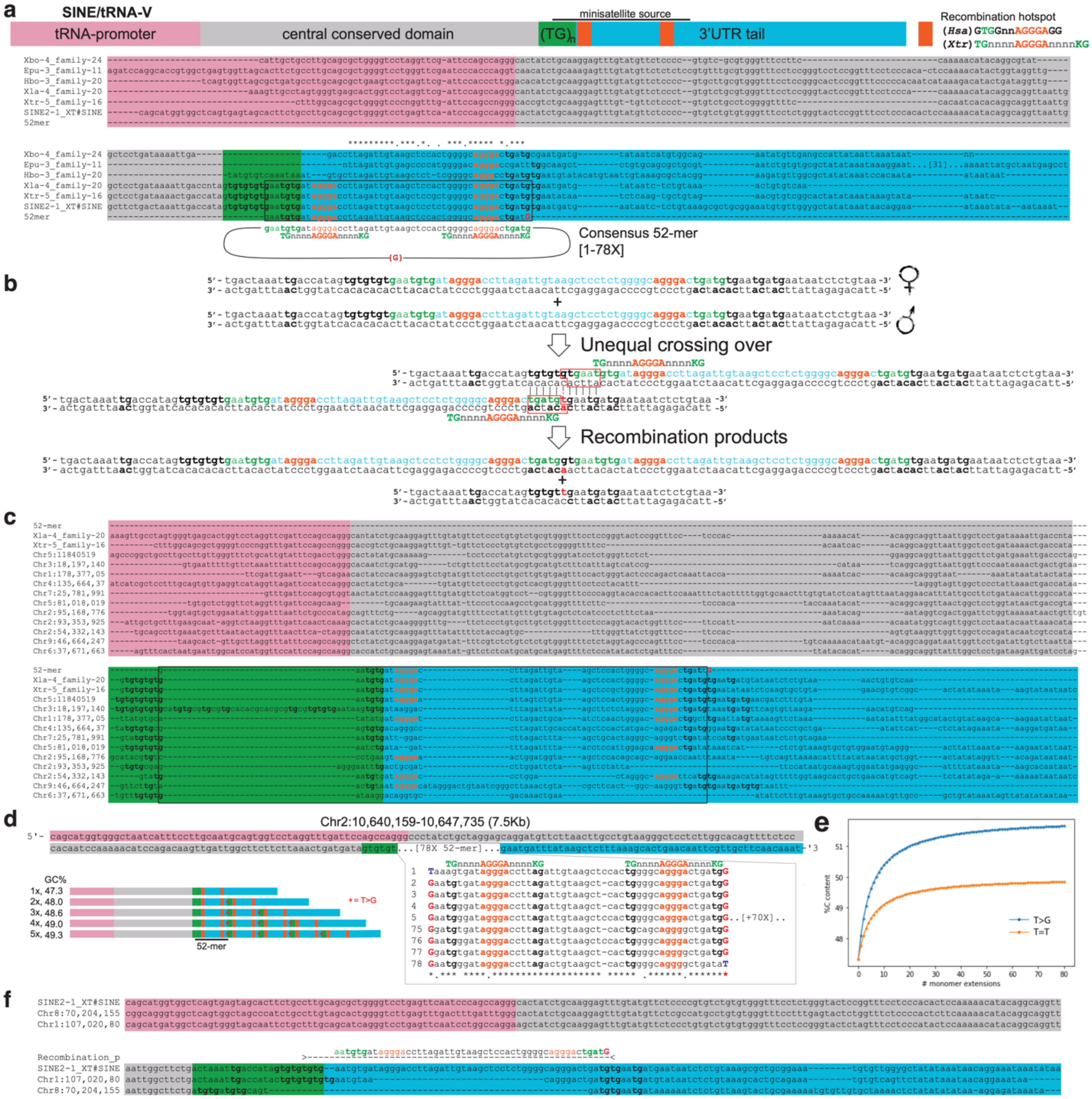

Supplementary Fig. 14 Microsatellite origin SINE/tRNA evolved into a microsatellite sequence.
(**a**) A schematic of the structure of *Xenopus* SINE/tRNA (top), as described by Ogiwara et al.[11]. The nucleotide sequence alignment of consensus SINE/tRNA obtained from different frog species (bottom) and the 52-mer sequence where the minisatellite sequence was derived. The multiple sequence alignment (MSA) of the consensus SINE/tRNA identified for the frogs in this study (except *E. coqui*) shows high levels of sequence similarity. MSA regions colored as in the schematic. The alignment includes the 52-bp monomer sequence that conforms to the minisatellite. The 52-mer consensus sequence shows perfect alignment with *X. tropicalis* (*Xtr*) and *X. laevis* (*Xla*). The last base of the monomer is a “G” substitution of a “T.” Notice that *E. pustulosus* (*Epu*)*, X. borealis* (*Xbo*)*,* and *H. boettgeri* (*Hbo*) lack the first “AGGA” box from the consensus sequence. (**b**) Representation of an unequal crossing-over event between a pair of unequally-aligned 3'UTR SINE/tRNA sequences. The unequal crossing over produces a duplication of the 51-mer + “G” (top) and a deletion of the sequence (bottom). (**c**) An example of the alignment of an intact SINE/tRNA in *X. tropicalis* genome. MSA regions colored as in the schematic in panel **a**. (**d**) Example of an ancestral SINE/tRNA (Chr2:10,640,159–10,647,735) that contains 78 copies of the 52-mer sequence and has extended over 7.5 kb in length. Sequence regions colored as in the schematic in panel **a**. (**e**) The GC% of the consensus SINE/tRNA oscillates between 46 and 48%, while the sequence of the monomer is slightly higher at 51% (51.9% with the addition to the T>G substitution), thus it would be expected that the extension of the tandem would cause a gradual increase in local GC content (see graph on the right). (**f**) Example of the deletion of the 52-mer that possibly resulted from unequal crossing over. MSA regions colored as in the schematic in panel **a**. *Hsa*, *Homo sapiens*; bp, basepairs; kb, kilobases. Source data are provided as a Source Data file.

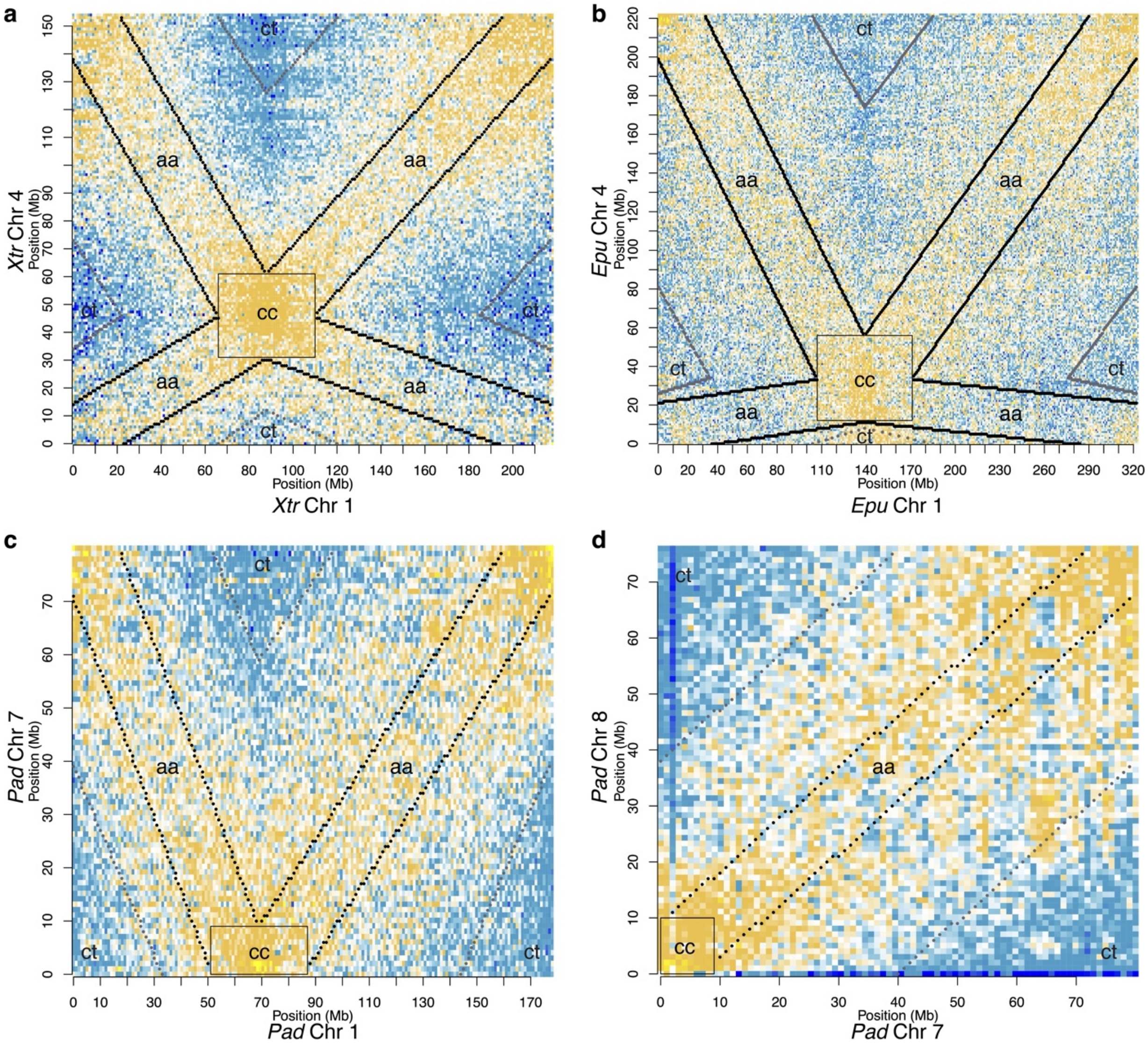

Supplementary Fig. 15 Enrichments of Rabl-like chromosome contacts.
Select inter-chromosomal Hi-C contact matrices exemplifying different chromosome-structure combinations observed. Contact enrichment, parity, and depletion between 1 Mb non-overlapping loci colored gold, white, and blue, respectively. Regions of each matrix sampled to measure median centromere-centromere (cc, black outlined box), centromere-telomere (ct, grey dotted boundary lines), and centromere-to-telomere polarity arm-arm (aa, black dotted boundary lines) contact densities are labeled.
(**a**) submetacentric *X. tropicalis* (*Xtr*) chromosomes 1 and 4; (**b**) submetacentric and acrocentric *E. pustulosus* (*Epu*) chromosomes 1 and 4, respectively; (**c**) metacentric and telocentric/acrocentric *P. adspersus* (*Pad*) chromosomes 1 and 7, respectively; and (**d**) telocentric/acrocentric *P. adspersus* (*Pad*) chromosomes 7 and 8. Mb, megabases. Source data are provided as a Source Data file.

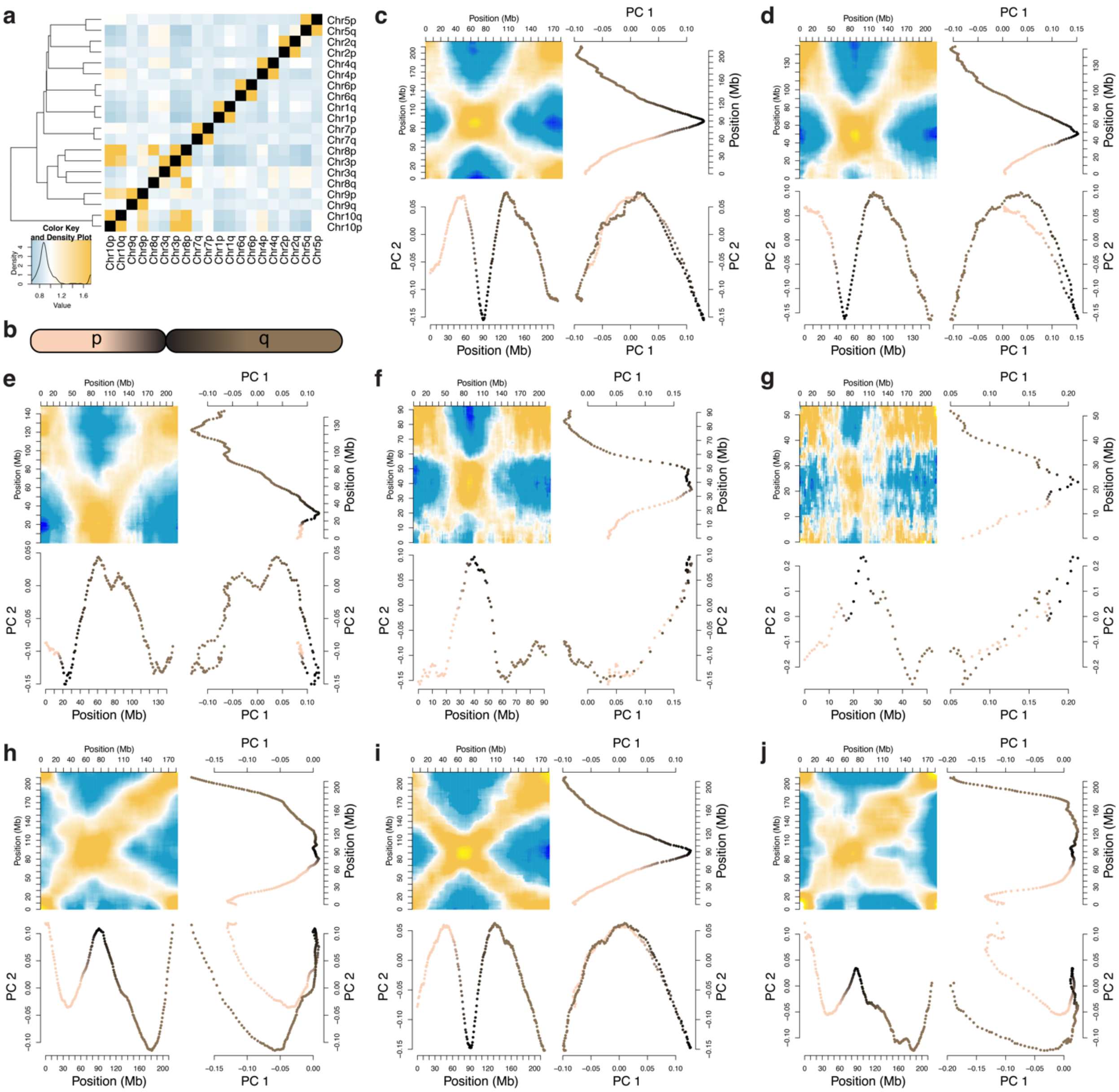

Supplementary Fig. 16 ***Xenopus tropicalis*** **3D chromatin structure and nuclear organization.**
(**a**) Genome-wide, a non-random association of chromosomal arm-arm contacts is observed (χ^2 (361, n = 28,366,570) = 58,25,879.96, Hochberg-corrected p < 4.46×10^{-308}; inter-arm relative range: 0.654–3.829). Chromosome arms are 2.60× enriched for intra-chromosomal contacts over inter-chromosomal contacts (χ^2 (1, n = 4,957,102) = 979,083.44, Hochberg-corrected p < 4.46×10^{-308}). Between chromosomes, p-p and q-q arms contacts are, respectively, 1.077× and 1.0412× enriched over p-q arm contacts (χ^2 (1, n = 24,786,496) = 17,037, Hochberg-corrected p < 4.46×10^{-308}). Note: 4.46×10^{-308} is the post-correction value for 2.23×10^{-308}, the lower numerical limit of a signed, double-precision floating-point value computed in the R statistical language on a 64-bit computer. See **Supplementary Table 21** for association statistics for all species. (**b**) In panels **c–j**, PC1 or PC2 vector points are colored tan, black, and brown to differentiate 1-Mb windows along the p-arm, near the centromere, and the q-arm, respectively; a schematic of this coloring scheme is shown for clarity. Visualizing example Rabl-like chromatin configurations for (**c**) chromosome 1, blood cell nuclei; (**d**) chromosome 4, blood cell nuclei; (**e**) chromosome 8, blood cell nuclei; (**f**) chromosome 9, blood cell nuclei; (**g**) chromosome 10, blood cell nuclei; (**h**) chromosome 1, sperm; (**i**) chromosome 1, NF stage 8; (**j**) chromosome 1, adult brain. All contact maps generated using contacts with mapping quality ≥ 30. For visualization, the contrast level for each Hi-C contact density map has been adjusted to accommodate each dataset. The target chromosome is plotted on the Y-axis of each heatmap, with the comparator chromosome on the X-axis. Contact enrichment, parity, and depletion between 1 Mb non-overlapping loci colored gold, white, and blue, respectively. PC, principal component; Mb, megabases. Source data are provided as a Source Data file.

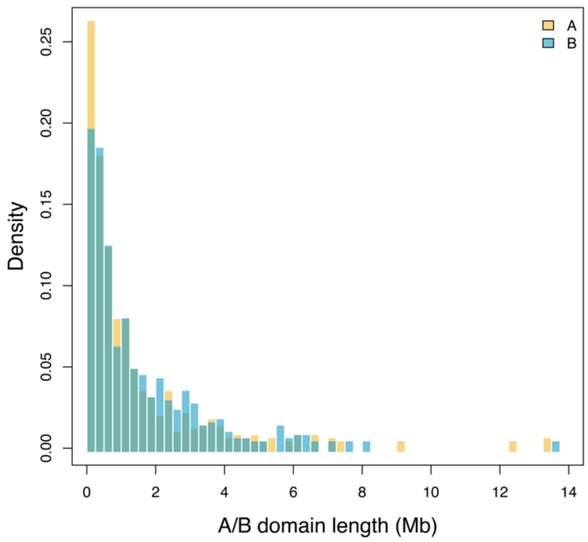

Supplementary Fig. 17 *Xenopus tropicalis* A/B compartment domain size distributions.
The size distributions for A (gold) and B (blue) compartment domains from *X. tropicalis* blood cell nuclei. Mb, megabases. Source data are provided as a Source Data file.

Supplementary Table 1 Sequence completeness of *Xenopus tropicalis* v9 and v10 assemblies.

Statistic	v9		v10	
Total assembled scaffold sequence	1,440,398,454	bp	1,451,261,802	bp
Total assembled contig sequence	1,369,865,365	bp	1,448,444,368	bp
Total placed contig sequence	1,227,064,696	bp	1,446,226,505	bp
Total unplaced contig sequence	142,800,669	bp	2,217,863	bp
Contig N50	5,552		32	
Contig N50 length	71,041	bp	14,634,335	bp
Contig NG50	8,291		41	
Contig NG50 length	51,188	bp	12,113,547	bp

Comparison between *X. tropicalis* v9 and v10 genome assembly statistics. NG50 calculations assume a genome size of 1.7 Gb[12].

Supplementary Table 2 Transcript coverage of *Xenopus tropicalis* v9 and v10 assemblies.

Transcript minimum Coverage (%)	v9 FTS (%)	v10 FTS (%)	v10 SCO (%)
90.0	97.32	99.67	99.71
75.0	98.12	99.80	99.83
50.0	98.76	99.92	99.94
25.0	99.17	99.96	99.97
10.0	99.38	99.96	99.97
<10.0	0.62	0.04	0.03

BUSCO	v9 Genome (%)	v10 Genome (%)	v10 Protein (%)
Complete	88.5	91.7	97.0
Single-copy	87.5	90.7	95.6
Multi-copy	1.0	1.0	1.4
Fragmented	6.0	4.4	1.5
Missing	5.5	3.9	1.5

Translated MGC minimum coverage (%)	v9 MGC (%)	v10 Translated MGC minimum identity (%)	v10 MGC (%)
95.0	98.18	100.0	90.99
80.0	99.38	95.0	99.60
65.0	99.65	90.0	99.89
50.0	99.86	85.0	99.96
35.0	99.95	80.0	99.99
20.0	100.00	75.0	100.00

Minimal coverage and identity percentages of FTS and SCO transcript sets, translated MGC cDNA clones, and BUSCO proteins mapped to the current assembly. BUSCO scores are derived with the BUSCO[13–15] v3.0.2-11-g1554283 pipeline run using tetrapoda_odb9 (n = 3,950). FTS, Full Transcript Set (n = 52,323); SCO, single-copy orthologs (n = 15,613), taken from Session et al.[7], defined using v9 assembly; MGC, Mammalian Gene Collection (n = 8,558).

Supplementary Table 3 Statistics summary of additional frog genome assemblies.

Genomic feature	*E. coqui*	*E. pustulosus*	*H. boettgeri*
Total scaffold length (bp)	2,789,403,129	2,592,984,374	3,214,299,233
Number of scaffolds	105,233	120,236	26,522
Scaffold N50 length (bp)	109,468,876	172,109,237	293,320,900
Total contig length (bp)	2,367,745,368	2,583,262,543	3,210,201,336
Number of contigs	480,045	137,711	42,879
Contig N50 length (bp)	10,801	295,193	783,846
Contigs sequence in chromosomes (%)	65.52	74.22	82.47
Contig GC content (%)	43.46	42.53	39.51
Masked contig repeat sequence (%)	56.10	47.92	48.60

Statistics were calculated using assembly-stats (commit 506a640, https://github.com/sanger-pathogens/assembly-stats).

Supplementary Table 4 *Xenopus tropicalis* protein-coding gene annotation summary statistics.

Statistic	Value
Annotation version	10.7
Taxonomy ID	8364
Primary transcripts (loci)	25,016
Alternate transcripts	37,983
Total transcripts	62,999
**Primary transcripts:**	
Average number of exons	8.7
Median exon length	137
Median intron length	1,075
Number of complete genes	24,167
Number of incomplete genes with start codon	276
Number of incomplete genes with stop codon	360
**Gene model support:**	
Number of genes with Pfam annotation	18,895
Number of genes with Panther annotation	20,727
Number of genes with KOG annotation	12,109
Number of genes with KEGG Orthology annotation	10,252
Number of genes with E.C. number annotation	5,175

Supplementary Table 5 Summary of annotations for additional frog genomes.

Annotation feature	*E. coqui*	*E. pustulosus*	*H. boettgeri*	*P. adspersus*
Number of genes / primary transcripts	23,346	30,613	20,684	18,673
Number of alternate transcripts	11,220	34,629	7,374	2,574
Total number of transcripts	34,566	65,242	28,058	21,247
Average number of exons per gene	7.3	6.5	8.5	8.8
Median exon length	137	141	133	133
Median intron length	1,752	1,002	1,483	1,089
Number of complete genes	19,899	25,989	18,244	16,861
Number of incomplete genes with start codon	1,149	1,417	872	637
Number of incomplete genes with stop codon	1,662	2,125	1,164	941
Number of genes with Pfam annotation	17,101	20,393	16,677	15,711
Number of genes with Panther annotation	19,243	23,598	18,610	17,098
Number of genes with KOG annotation	9,633	10,365	10,162	10,347
Number of genes with KEGG Orthology annotation	9,799	10,528	9,973	9,779
Number of genes with E.C. number annotation	4,795	6,199	4,684	4,211

Statistics were calculated by the DOE-JGI Integrated Gene Call (IGC) pipeline[16].

Supplementary Table 6 BUSCO genome scores of additional frog genome assemblies.

	H. boettgeri		*P. adspersus*		*E. pustulosus*		*E. coqui*	
**BUSCO**	**Genome**	**Protein**	**Genome**	**Protein**	**Genome**	**Protein**	**Genome**	**Protein**
Complete	80.0	88.4	87.5	88.6	75.7	80.2	76.4	82.5
Single-copy	77.7	85.4	86.3	86.6	72.4	76.0	75.4	80.7
Multi-copy	2.3	3.0	1.2	2.0	3.3	4.2	1.0	1.8
Fragmented	9.7	6.7	6.2	6.1	11.9	12.0	12.1	11.1
Missing	10.3	4.9	6.3	5.3	12.4	7.8	11.5	6.4

The following BUSCO scores are derived with the BUSCO[13–15] v3.0.2-11-g1554283 pipeline run using tetrapoda_odb9 (n = 3,950). Percentages are shown for both genome and proteome BUSCO runs.

Supplementary Table 7 *Xenopus tropicalis* repeat abundances.

Repeat type / family	% in Genome	Repeat type / family	% in Genome
**DNA Transposon**	**26.302%**	**LTR Retrotransposon**	**3.312%**
hAT	6.380%	Ty3	1.879%
Kolobok	6.048%	DIRS	0.556%
Harbinger	6.015%	LTR	0.282%
Mariner	4.057%	Ngaro	0.211%
piggyBac	1.882%	Endogenous Retrovirus	0.159%
DNA	1.295%	Copia	0.105%
RC	0.647%	BEL	0.071%
DNA transposon	0.522%	Pao	0.069%
CMC	0.120%		
MULE	0.062%	**Non-LTR Retrotransposon**	**7.197%**
Polinton	0.023%	LINE	6.847%
Novosib	0.019%	SINE	0.351%
Sola	0.009%	SINE?	0.004%
Zisupton	0.002%		
Merlin	0.002%	**Other**	**0.228%**
Ginger	0.001%	Transposable Element	0.188%
Crypton	0.001%	Interspersed repeat	0.008%
Maverick	0.001%	Inverted repeat	0.001%
		tRNA	0.010%
**Tandem Repeats***	**3.817%**	rRNA	0.011%
Satellite	2.889%	snRNA	0.010%
Simple repeat	0.932%	Low complexity	0.093%

Supplementary Table 8 Ancestral chromosome fusions.

Species and chromosome	Fusion region	Fused ancestral chromosomes
E. coqui Chr1	167,326,341–167,391,435	B+F
E. coqui Chr4	64,368,134–64,506,892	H+B
E. coqui Chr5	90,030,948–90,111,922	E+F
E. coqui Chr6	51,920,009–52,085,381<br>62,174,693–62,190,413<br>74,807,529–74,967,042	K+I<br>I+K<br>K+I
E. pustulosus Chr6	78,294,430–79,251,447	M+I
E. pustulosus Chr7	67,080,716–67,193,535	K+D
H. boettgeri Chr4	132,010,981–132,334,876	D+E
H. boettgeri Chr7	112,455,236–114,827,918	H+I
H. boettgeri Chr8_10	103,984,075–104,182,343	M+J
H. boettgeri Chr8_10	227,189,815–227,531,594	J+K
P. adspersus Chr3	27,789,267–27,840,023	M+A
P. adspersus Chr6	25,252,335–25,589,174	M+A
X. tropicalis Chr4	76,607,359–76,630,690	D+E
X. tropicalis Chr7	63,487,360–63,537,129	H+I
X. tropicalis Chr8	81,812,475–81,878,653	J+K

Locations of ancestral chromosome fusions in the examined species using runs of collinearity from *L. ailaonicum* containing at least 1 kb of aligned sequence.

Supplementary Table 9 N50 lengths for collinear runs of orthologous genes between frogs.

Species	*L. ailaonicum*	*P. adspersus*	*E. pustulosus*	*E. coqui*	*H. boettgeri*	*X. tropicalis*	*X. laevis* L
P. adspersus	90	–	–	–	–	–	–
E. pustulosus	89	221	–	–	–	–	–
E. coqui	91	119	150	–	–	–	–
H. boettgeri	105	162	168	101	–	–	–
X. tropicalis	113	175	169	125	323	–	–
X. laevis L	122	167	186	127	346	1064	–
X. laevis S	111	163	199	116	245	688	676

N50 lengths for chromosomal collinear runs of gene orthologs, requiring each run to contain five or more gene orthologs. Calculated with cluster-collinear-bedpe (v0.0.1, https://bitbucket.org/bredeson/artisanal).

Supplementary Table 10 Four-fold degenerate nucleotide divergences.

Species	*A. mexicanum*	*L. ailaonicum*	*P. adspersus*	*E. pustulosus*	*E. coqui*	*H. boettgeri*	*X. tropicalis*	*X. laevis* L
L. ailaonicum	1.686	–	–	–	–	–	–	–
P. adspersus	1.704	1.039	–	–	–	–	–	–
E. pustulosus	1.730	1.065	0.753	–	–	–	–	–
E. coqui	1.705	1.040	0.728	0.356	–	–	–	–
H. boettgeri	1.622	1.067	1.085	1.111	1.086	–	–	–
X. tropicalis	1.516	0.962	0.980	1.005	0.981	0.562	–	–
X. laevis L	1.537	0.982	1.000	1.026	1.001	0.582	0.206	–
X. laevis S	1.550	0.995	1.013	1.039	1.014	0.595	0.218	0.166

Pairwise nucleotide divergence in substitutions per site based on four-fold degeneracy between the examined species extracted from the RAxML[17] (v8.2.11) phylogenetic tree using Newick utilities[18] (v1.6).

Supplementary Table 11 Estimation of divergence times.

Node	TimeTree input minimum	TimeTree input maximum	MEGA7 output estimate
E. coqui – *E. pustulosus*	71	108	74
E. coqui – *P. adspersus*	147	162	149
E. coqui – *L. ailaonicum*	167	205	202
X. laevis L – *X. laevis* S	–	–	38
X. laevis L – *X. tropicalis*	34	79	52
X. laevis L – *H. boettgeri*	104	158	126
X. laevis L – *E. coqui*	187	220	205

Divergence time input intervals from TimeTree[19] and estimated output times from MEGA7[20] (v7.0.26) in millions of years ago.

Supplementary Table 12 Centromeric tandem repeat monomer lengths and counts.

X. tropicalis	*X. tropicalis* centromeric tandem repeat (205 bp monomer)			
chromosome	Start	End	Length (kb)	# monomers
Chr1	89,211,804	89,262,389	50.585	163
Chr2	67,457,118	67,564,135	107.017	512
Chr3	16,701,143	16,799,031	97.888	267
Chr4	46,570,283	46,621,730	51.447	222
Chr5	61,989,064	62,041,259	52.195	227
Chr6	73,071,389	73,112,569	41.180	175
Chr7	60,256,156	60,514,842	258.686	767
Chr8	21,339,510	21,551,929	212.419	834
Chr9	42,101,630	42,147,670	46.040	207
Chr10	21,179,881	21,271,318	91.437	233

Supplementary Table 13 Mapping statistics for ChIP-seq samples.

Sample	Total reads (Millions)	% mapped	% properly paired	% singletons	% mate mapped to different chr	Enrichment		
						CTR region	No-CTR region	CTR / Non-CTR
In-1	60.72	96.93%	78.68%	0.83%	1.46%	1065.6	410.2	2.6
In-2	43.60	96.49%	77.70%	1.25%	1.60%	834.8	292.5	2.9
In-3	80.31	96.53%	78.21%	1.24%	1.14%	1539.2	539.1	2.9
Ig-2	4.15	96.64%	80.58%	1.27%	1.21%	83.7	27.9	3.0
Ig-3	45.93	97.37%	82.03%	1.01%	1.02%	2246.9	742.8	3.0
H3-2	66.02	96.68%	79.64%	1.23%	1.19%	1371.6	443.8	3.1
H3-3	110.50	96.68%	81.26%	1.22%	1.07%	1145.1	311.1	3.7
H4-1	52.94	97.39%	85.16%	0.65%	1.07%	1613.5	359.2	4.5
CA-2	54.48	96.98%	80.25%	0.67%	1.16%	1600.3	92.7	17.3
CA-3	13.84	97.11%	81.47%	1.11%	1.10%	35698.8	1563.8	22.8
CA-1	233.54	97.39%	82.45%	1.02%	1.01%	9855.7	363	27.2

Supplementary Table 14 Correlates of recombination rate.

Genetic map v1	Recomb. Rate Correlation	*p*-value*	Median Jungle	Median Desert	Delta	Ratio
Recombination Rate	1.00	0.00	2.511	0.146	2.37	0.89
PCA1	0.76	0.00	13.768	−5.885	19.65	2.49
Satellite: Xtr-5_family-21	0.68	0.00	49	5	44.00	0.81
Unknown: Xbo-6_family-1312	0.67	0.00	7	0	7.00	1.00
CpG	0.66	0.00	32934	24550	8384.00	0.15
GC%	0.65	0.00	43.816	38.843	4.97	0.06
Satellite	0.62	0.00	131	57	74.00	0.39
MSAT	0.59	0.00	3	0	3.00	1.00
SINE/tRNA-V: Xla-4_family-206	0.58	0.00	47	3	44.00	0.88
SINE/tRNA-V	0.58	0.00	51	6	45.00	0.79
CTCTCCC	0.56	0.00	338	132	206.00	0.44
DNA/PIF-Harbinger: Xtr-3_family-37	0.56	0.00	13	4	9.00	0.53
MSAT: MSAT4_XT	0.55	0.00	2	0	2.00	1.00
CTCF_Hashimoto	0.54	0.00	28	11	17.00	0.44
Unknown: Xtr-4_family-155	0.54	0.00	4	0	4.00	1.00
CCCCCCC	0.51	0.00	520	194	326.00	0.46
Unknown: Xbo-3_family-280	0.47	0.00	5	0	5.00	1.00
CpG_ctrl	0.47	0.00	95898	88169	7729.00	0.04
Rec_hotspot	0.43	0.00	193	141	52.00	0.16
Cohesin	0.42	0.00	22	8	14.00	0.47
PCA2	0.27	1.72×10^{-245}	4.039	−4.094	8.13	−147.87
LINE/CR1	0.22	9.17×10^{-151}	68	57	11.00	0.09
PCA3	0.17	2.78×10^{-98}	0.092	−2.172	2.26	−1.09
N	0.10	3.18×10^{-31}	0	0	0.00	NaN
Ty3/Metaviridae	0.00	6.29×10^{-1}	14	16	−2.00	−0.07
DNA/TcMar-Tigger: Xbo-2_family-40	−0.10	5.13×10^{-32}	6	7	−1.00	−0.08
DNA/PiggyBac: Xtr-5_family-424	−0.10	5.60×10^{-35}	0	2	−2.00	−1.00
piggyBac: piggyBac-N2_XT	−0.13	1.68×10^{-55}	0	2	−2.00	−1.00
Harbinger: Harbinger-1_XT	−0.14	7.86×10^{-65}	1	2	−1.00	−0.33
piggyBac	−0.15	8.96×10^{-75}	11	15	−4.00	−0.15
DNA_transposon: DNA1_Xt	−0.15	1.01×10^{-75}	13	16	−3.00	−0.10
piggyBac: piggyBac-N1_XT	−0.18	2.32×10^{-102}	0	1	−1.00	−1.00
DNA/PiggyBac	−0.19	2.94×10^{-121}	14	20	−6.00	−0.18
DNA/TcMar-Tigger	−0.20	1.93×10^{-126}	89	109	−20.00	−0.10
piggyBac: piggyBac-N2A_XT	−0.21	7.55×10^{-147}	0	1	−1.00	−1.00
DNA_transposon: DNA10_XT	−0.25	3.59×10^{-205}	6	10	−4.00	−0.25
L1	−0.26	5.55×10^{-227}	5	12	−7.00	−0.41
LINE/Penelope	−0.28	5.44×10^{-257}	37	48	−11.00	−0.13
CR1: CR1_1a_XT	−0.30	1.62×10^{-297}	4	7	−3.00	−0.27
Helitron: Helitron-N1A_XT	−0.34	0.00	0	3	−3.00	−1.00
DNA_transposon	−0.34	0.00	27	41	−14.00	−0.21
DNA	−0.35	0.00	83	119	−36.00	−0.18
Kolobok: Kolobok-1N3_XT	−0.36	0.00	0	2	−2.00	−1.00
DNA/Kolobok-T2: Xtr-1_family-281	−0.38	0.00	0	1	−1.00	−1.00
DNA/TcMar-Tigger: Xtr-2_family-11	−0.38	0.00	8	15	−7.00	−0.30
DNA_transposon: DNA6_XT	−0.40	0.00	2	6	−4.00	−0.50
LINE/CR1: Xtr-4_family-283	−0.41	0.00	2	7	−5.00	−0.56
Unknown: Xtr-1_family-262	−0.42	0.00	0	3	−3.00	−1.00
DNA/Kolobok-T2: Xtr-1_family-33	−0.51	0.00	0	4	−4.00	−1.00
DNA/Kolobok-T2: Xtr-1_family-31	−0.53	0.00	0	4	−4.00	−1.00
Mariner/Tc1: DNA4_Xt	−0.59	0.00	7	23	−16.00	−0.53
DNA/TcMar-Tigger: Xtr-4_family-702	−0.61	0.00	4	21	−17.00	−0.68
Pos_Rel_to_end	−0.72	0.00	16.167	17.784	−1.62	−0.05

*p-values corrected for multiple comparisons using Hochberg's step-up procedure.

Supplementary Table 15 Subtelomeric enrichment for tandem repeats.

Chromosome	Subtelomere	Non-subtelomere	Ratio
Chr1	23.68%	3.59%	6.6
Chr2	24.27%	2.76%	8.79
Chr3	26.26%	3.08%	8.53
Chr4	24.83%	3.99%	6.22
Chr5	24.17%	3.72%	6.5
Chr6	23.36%	3.49%	6.69
Chr7	22.76%	3.46%	6.58
Chr8	32.63%	3.99%	8.18
Chr9	20.60%	3.85%	5.35
Chr10	20.28%	5.82%	3.48

Sampled from 25.0 Mb from chromosome ends non-overlapping with pericentromeres.

Supplementary Table 16 Correspondence of monomer sequence with annotated repeat elements.

ID	Size	Type	Class/Family	Subfamily	Monomer region
a	205	Satellite	Satellite	Centromeric Tandem Repeat	Chr10:21,202,863–21,221,377:206;90.0;206
b	140	DNA	PIF-Harbinger	Harbinger-1_XT	Chr4:44,687,900–44,688,471:140;4.1;140
c	58	Non-LTR	LINE/CR1	CR1-2_XT, CR1-1_XL	Chr1:5,339,979–5,340,451:58;8.2;58
d	134	DNA	PIF-Harbinger	Xtr-1_family-625, Xtr-1_family-181	Chr9:43,007,496–43,007,890:132;2.9;134
e	55	DNA	PiggyBac	Xtr-4_family-150, Xtr-6_family-62	Chr6:78,080,043–78,080,155:55;2.1;55
f	57	N/A	N/A	N/A	Chr7:130,271,437–130,276,838:57;94.8;57
g	104	N/A	N/A	N/A	Chr10:15,393,501–15,394,099:104;5.8;104
h	112	Non-LTR	CR1	Xtr-5_family-2393, Xla-1_family-417	Chr2:177,493,031–177,494,141:112;9.9;112
i	82	N/A	N/A	N/A	Chr7:3,459,827–3,460,032:84;2.5;82
j	135	Satellite	MSAT	MSAT1_XT	Chr10:16,832,997–16,834,248:135;9.3;135
k	71	N/A	N/A	N/A	Chr1:214,792,050–214,792,755:71;9.9;71
l	100	Satellite	SAT	SAT-1_XL	Chr4:142,973,443–142,974,440:100;9.9;100
m	93	Satellite	SAT	Xtr-5_family-2049	Chr6:148,956,044–148,956,867:93,9.5,93
n	132	N/A	N/A	N/A	Chr1:210,557,533–210,557,838:135;2.3;132
o	61	N/A	N/A	N/A	Chr6:74,371,563–74,371,758:61;3.2;61
p[*]	52	Non-LTR	SINE/tRNA-V	SINE2-1_XT	Chr3:13,767,515–13,767,949:52;8.5;52
q	113	Satellite	Satellite	DNA4Sat_Xt, Xtr-5_family-21	Chr10:15,491,890–15,492,956:113;9.4;113

[*]See **Supplementary Table 17**.

Supplementary Table 17 Copy counts of 52-mer minisatellite.

Species	Count
X. tropicalis	41,014
X. laevis	17,375
N. parkeri	3,738
H. boettgeri	305
L. ailaonicum	128
E. pustulosus	46

Number of BLAST hits of 52-mer monomer sequence derived from SINE2/tRNA in frog genome assemblies.

Supplementary Table 18 Enrichment of Rabl-like chromosome contacts.

Species/tissue	Inter-arm*	Inter-centromere*	ACA[21] score
X. tropicalis blood cell nuclei	1.813	3.200	0.842
X. tropicalis stage 8†	1.640	3.041	1.426
X. tropicalis stage 9†	1.831	5.195	1.661
X. tropicalis stage 10†	2.281	7.926	2.026
X. tropicalis stage 11†	2.537	7.971	2.463
X. tropicalis stage 12†	2.769	8.623	2.365
X. tropicalis stage 13†	2.687	8.001	2.521
X. tropicalis stage 15†	3.070	9.819	3.082
X. tropicalis stage 17†	3.276	10.689	2.305
X. tropicalis stage 23†	2.335	6.567	1.978
X. tropicalis brain†	1.209	1.257	2.244
X. tropicalis liver†	1.514	2.212	1.015
X. tropicalis sperm†	1.056	1.201	0.614
E. coqui	1.133	1.712	−2.920
E. pustulosus	1.318	1.859	0.736
H. boettgeri	1.300	1.971	0.725
L. ailaonicum	1.525	3.408	1.707
P. adspersus	1.036	1.190	1.944
X. laevis L	1.542	4.379	2.170
X. laevis S	1.887	5.856	0.664

†Data from Niu et al.[22].
*Contact density enrichment values reported are relative to centromere-telomere contact densities.

Supplementary Table 19 Strength and significance of Rabl-like chromosome folding structure in *Xenopus tropicalis*.

Chr	Blood cell nuclei	Stage 8†	Stage 9†	Stage 10†	Stage 11†	Stage 12†	Stage 13†
1	0.213***	0.078***	0.125***	0.136***	0.157***	0.155***	0.202***
2	0.031***	0.022***	0.010***	0.009***	0.004***	0.003***	0.008***
3	0.233***	0.402***	0.524***	0.586***	0.364***	0.273***	0.341***
4	0.101***	0.057***	0.194***	0.159***	0.103***	0.100***	0.105***
5	0.017***	0.013***	0.013***	0.008***	0.008***	0.013***	0.012***
6	0.185***	0.064***	0.059***	0.060***	0.062***	0.067***	0.058***
7	0.076***	0.042***	0.026***	0.043***	0.038***	0.044***	0.049***
8	0.027***	0.141***	0.135***	0.143***	0.066***	0.053***	0.045***
9	0.163***	0.499***	0.411***	0.491***	0.674***	0.676***	0.412***
10	0.421***	0.061**	0.076***	0.076***	0.038***	0.062***	0.074***
**Sum**	**1.465**	**1.376**	**1.573**	**1.711**	**1.514**	**1.447**	**1.308**

Chr	Stage 15†	Stage 17†	Stage 23†	Liver†	Brain†	Sperm†
1	0.202***	0.211***	0.176***	0.952***	1.260***	0.864***
2	0.018***	0.020***	0.015***	0.687***	0.885***	0.875***
3	0.246***	0.223***	0.080***	0.113***	0.179***	0.265***
4	0.098***	0.112***	0.050***	0.354***	1.077***	0.596***
5	0.025***	0.032***	0.037***	0.584***	0.432***	0.675***
6	0.056***	0.063***	0.068***	0.454***	0.745***	0.553***
7	0.050***	0.056***	0.067***	0.070***	0.123***	0.546***
8	0.042***	0.042***	0.036***	0.031***	0.203***	0.362***
9	0.379***	0.348***	0.443***	0.170***	1.146***	0.914**
10	0.096***	0.117***	0.118***	0.559***	0.441*	0.635***
**Sum**	**1.213**	**1.224**	**1.088**	**3.975**	**6.490**	**6.285**

Numerical values reported are the residual sum of squares (RSS) estimates for the constraint of Rabl-like folding structure; i.e., the degree to which p and q arms of the same chromosome freely interact along their lengths. Significance p-values (uncorrected) calculated by permutation testing (one-sided; 10,000 iterations) (**Supplementary Note 5**). Triple-asterisks ("***") label chromosomes with $p \leq 1\times10^{-4}$, double-asterisks ("**") denote $p = 2\times10^{-4}$, and chromosomes with $p = 3\times10^{-4}$ are labled with single-asterisks ("*"). All stage-chromosome combinations maintain $p = 3.0\times10^{-4}$ after Hochberg family-wise p-value correction.
†Data from Niu et al.[22].

Supplementary Table 20 Gene and repeat densities associated with A/B compartment structure

Class	Compartment sample size (n)		D statistic	p-value*
**Gene density**	**A**	**B**		
Compartment (genome-wide)	2,745	3,056	0.42957	1.0×10^{-6}
Chr1	379	491	0.45111	9.9×10^{-6}
Chr2	356	369	0.46881	9.9×10^{-6}
Chr3	242	374	0.38454	9.9×10^{-6}
Chr4	326	290	0.51980	9.9×10^{-6}
Chr5	288	369	0.40108	9.9×10^{-6}
Chr6	259	359	0.40049	9.9×10^{-6}
Chr7	262	273	0.42070	9.9×10^{-6}
Chr8	313	276	0.40656	9.9×10^{-6}
Chr9	183	182	0.36516	9.9×10^{-6}
Chr10	137	73	0.39466	2.0×10^{-5}
**Repeat density**				
DNA/Kolobok-T2	518	515	0.48289	9.9×10^{-6}
DNA/hAT-Charlie	518	514	0.26881	9.9×10^{-6}
DNA/TcMar-Tc1	511	508	0.35489	9.9×10^{-6}
Mariner/Tc1	517	513	0.21499	9.9×10^{-6}
DIRS	464	440	0.18064	7.4×10^{-5}
LTR_Retrotransposon	411	438	0.21924	9.9×10^{-6}
Transponsable_Element	462	478	0.23253	9.9×10^{-6}
hAT	518	515	0.14429	2.9×10^{-5}
Ty3-Metaviridae	501	501	0.34731	9.9×10^{-6}
DNA_transposon	516	508	0.29691	9.9×10^{-6}
LINE/CR1	518	514	0.22322	9.9×10^{-6}
LTR/Ty3-Metaviridae	511	514	0.35610	9.9×10^{-6}
DNA/TcMar-Tigger	517	513	0.17939	2.8×10^{-5}
DNA	518	513	0.41570	9.9×10^{-6}
DNA/hAT-Ac	517	513	0.61520	9.9×10^{-6}

*Two-sample Kolmogorov-Smirnov two-sided test p-values (10M Monte Carlo repititions) corrected for multiple comparisons using Hochberg's step-up procedure.

Supplementary Table 21 Contact enrichment among chromosomes and chromosome arms.

Interchromosomal enrichment	Degrees of Freedom (df)	Sample size (n)	χ^2 statistic	p-value†	Relative Enrichment Min.	Relative Enrichment Max.
E. coqui	144	6,918,603	769,032.76	$< 4.46\times10^{-308}$	0.714	1.602
E. pustulosus	100	21,963,827	2,650,230.54	$< 4.46\times10^{-308}$	0.853	1.313
H. boettgeri	64	11,472,000	1,544,949.63	$< 4.46\times10^{-308}$	0.874	1.190
L. ailaonicum	144	57,076,729	7,466,579.43	$< 4.46\times10^{-308}$	0.680	1.723
P. adspersus	121	128,641,903	13,700,811.18	$< 4.46\times10^{-308}$	0.882	1.201
*P. adspersus**	169	144,706,271	14,272,071.75	$< 4.46\times10^{-308}$	0.626	18.763
X. laevis	289	23,901,183	1,519,869.44	$< 4.46\times10^{-308}$	0.924	1.281
X. tropicalis	81	24,987,749	3,049,787.53	$< 4.46\times10^{-308}$	0.828	1.168

Intrachromosomal interarm contact enrichment	Degrees of Freedom (df)	Sample size (n)	χ^2 statistic	p-value†	Relative Enrichment Min.	Relative Enrichment Max.
E. coqui	625	8,199,375	6,186,850.95	$< 4.46\times10^{-308}$	0.256	10.999
E. pustulosus	441	26,317,421	11,057,282.37	$< 4.46\times10^{-308}$	0.610	5.842
H. boettgeri	289	14,196,106	7,422,295.55	$< 4.46\times10^{-308}$	0.510	6.145
L. ailaonicum	625	71,298,515	54,254,909.53	$< 4.46\times10^{-308}$	0.317	7.842
P. adspersus	529	143,487,710	27,537,888.69	$< 4.46\times10^{-308}$	0.618	4.401
*P. adspersus**	729	160,073,454	29,474,072.39	$< 4.46\times10^{-308}$	0.626	18.763
X. laevis	1225	26,203,393	7,391,101.40	$< 4.46\times10^{-308}$	0.579	5.781
X. tropicalis	361	28,366,571	5,825,879.96	$< 4.46\times10^{-308}$	0.654	3.830

Enrichment of intrachromosomal over interchromosomal contacts	Degrees of Freedom (df)	Sample size (n)	χ^2 statistic	p-value†	Relative enrichment
E. coqui	1	1,634,268	691,927.92	$< 4.46\times10^{-308}$	4.725
E. pustulosus	1	5,644,909	2,131,647.86	$< 4.46\times10^{-308}$	4.188
H. boettgeri	1	3,486,504	1,215,445.75	$< 4.46\times10^{-308}$	3.883
L. ailaonicum	1	17,073,360	8,945,125.49	$< 4.46\times10^{-308}$	6.242
P. adspersus	1	21,829,747	4,806,499.22	$< 4.46\times10^{-308}$	2.768
*P. adspersus**	1	22,157,873	5,585,252.08	$< 4.46\times10^{-308}$	3.017
X. laevis	1	3,332,189	1,136,530.06	$< 4.46\times10^{-308}$	3.808
X. tropicalis	1	4,957,102	979,083.44	$< 4.46\times10^{-308}$	2.600

Enrichment of p-p and q-q arm contacts over p-q arm contacts	Degrees of Freedom (df)	Sample size (n)	χ^2 statistic	p-value†	Relative enrichment
E. coqui	1	6,850,547	3,914.92	$< 4.46\times10^{-308}$	0.928
E. pustulosus	1	21,760,538	4,752.74	$< 4.46\times10^{-308}$	1.032
H. boettgeri	1	11,423,575	8,322.19	$< 4.46\times10^{-308}$	1.061
L. ailaonicum	1	56,582,771	8,462.43	$< 4.46\times10^{-308}$	1.026
P. adspersus	1	127,451,200	3,059.42	$< 4.46\times10^{-308}$	1.011
*P. adspersus**	1	143,432,205	160.96	$= 9.99\times10^{-37}$	1.002
X. laevis	1	23,564,270	59,815.60	$< 4.46\times10^{-308}$	1.119
X. tropicalis	1	24,786,496	17,037.87	$< 4.46\times10^{-308}$	1.059

†Chi-squared test p-values corrected for multiple comparisons using Hochberg's step-up procedure; note: 4.46×10^{-308} is the post-correction value for 2.23×10^{-308}, the lower numerical limit of a signed, double-precision floating-point value computed in the R statistical language on a 64-bit computer.
*Sex chromosomes included in calculations.

Supplementary Table 22 Relative enrichment of Hi-C contacts among chromosomes.

X. tropicalis	**Chr1**	**Chr2**	**Chr3**	**Chr4**	**Chr5**	**Chr6**	**Chr7**	**Chr8**	**Chr9**
Chr2	1.090	–	–	–	–	–	–	–	–
Chr3	1.069	1.018	–	–	–	–	–	–	–
Chr4	1.058	1.017	0.999	–	–	–	–	–	–
Chr5	1.052	1.069	0.997	1.001	–	–	–	–	–
Chr6	1.044	1.016	0.984	1.000	1.010	–	–	–	–
Chr7	1.010	0.992	0.971	0.981	0.990	0.999	–	–	–
Chr8	1.041	0.999	1.052	0.984	0.978	0.968	0.954	–	–
Chr9	0.952	0.952	0.916	0.952	0.958	0.981	0.983	0.929	–
Chr10	0.828	0.876	0.878	0.943	0.890	0.962	1.000	0.971	1.168

Supplementary Note 1: High-throughput sequencing of *Xenopus tropicalis*.

High molecular weight DNA isolation

DNA was isolated from blood cells of an *X. tropicalis* F_{17} Nigerian strain female using a method similar to that described in Mitros et al.[4] (2019). Briefly, blood was collected from a benzocaine-anesthetized frog into a 15 mL polypropylene tube filled with 0.85× SSC. The tube was capped and inverted to mix and then spun down at 900 *g* for 5 min. The supernatant was removed by decanting. Cells were resuspended in 10 mL 0.85× SSC and then spun down again at 500 *g* for 5 min. The supernatant was removed and cells were resuspended in 10 mL lysis buffer (1% SDS, 20 mM EDTA, 100 mM NaCl, 20 mM Tris pH 7.5–8.0; supplemented with 200 µg/ml Proteinase K) and incubated overnight at 55°C. Phenol extraction was performed with an equal volume of buffered phenol and mixed very gently end-over-end overnight, then spun at 900 *g* for 5 min. DNA was precipitated from the aqueous phase by the addition of 1/10 vol 3 M ammonium acetate, thorough mixing, then the addition of 0.6 vol isopropanol, then more thorough mixing. DNA was spooled with an end-closed Pasteur pipet and scraped into a tube of 70% EtOH, then spooled again into a clean tube and all traces of EtOH were removed. DNA was resuspended in 1 mL of TE pH 8.0. This DNA was used for Pacific Biosciences (PacBio) Single-Molecule Real-Time (SMRT), 10x Genomics Chromium Genome linked-read, and Illumina whole-genome shotgun (WGS) sequencing libraries.

PacBio SMRT sequencing

DNA from the F_{17} Nigerian strain female was sent to HudsonAlpha Institute for Biotechnology (HAIB) for library preparation and sequencing. A total of 4.83 million SMRT continuous long reads (CLR) were generated using a PacBio RSII with P6-C4 chemistry, resulting in 65.4 Tb and an estimated 38.5× depth of coverage. Reads were filtered for lengths of 3 kb or longer; half of the sequenced bases were captured in reads 18.7 kb or longer. The sequences are deposited in the NCBI under BioProject PRJNA577946 [https://www.ncbi.nlm.nih.gov/bioproject/PRJNA577946] with SRA accession codes SRR10427757–SRR10427759, SRR10427764–SRR10427783, SRR10427786–SRR10427826.

10x Genomics Chromium Genome linked reads

We generated 366.2 million pairs (65× sequencing depth) of Illumina 2×151 bp sequences on a HiSeq X Ten from a 10x Genomics Chromium Genome paired-end linked-read library prepared and sequenced at HAIB from DNA from an F_{17} Nigerian strain female frog. The average molecule length from 1.36 million Chromium GEMs was inferred to be 55.3 kb, with 84.8% of molecules estimated to be 20 kb or longer, and 20% of molecules 100 kb or longer. The sequences are deposited in the NCBI under BioProject PRJNA577946 [https://www.ncbi.nlm.nih.gov/bioproject/PRJNA577946] with SRA accession code SRR10427785 [https://www.ncbi.nlm.nih.gov/sra/?term=SRR10427785].

In vivo, high-throughput chromatin conformation capture (Hi-C)

Three DpnII Hi-C libraries were constructed by Dovetail Genomics LLC from blood and sequenced by the QB3 Vincent J. Coates Genomics Sequencing Laboratory (VGCSL) at the University of California, Berkeley (UCB) for 381,335,524 total pairs (**Supplementary Data 1**). The sequences are deposited in the NCBI under BioProject PRJNA577946 [https://www.ncbi.nlm.nih.gov/bioproject/PRJNA577946] with SRA accession codes SRR10427762 [https://www.ncbi.nlm.nih.gov/sra/?term=SRR10427762], SRR10427763 [https://www.ncbi.nlm.nih.gov/sra/?term=SRR10427763], and SRR10427784 [https://www.ncbi.nlm.nih.gov/sra/?term=SRR10427784].

Shotgun sequencing

Whole-genome shotgun mate-pair Sanger sequencing of the F_7 female Nigerian strain used in this study is described by Mitros et al.[4]. Additionally, a 670 bp-insert library was prepared by the Functional Genomics Laboratory (FGL) at UCB from the female F_{17} Nigerian strain genomic DNA using the KAPA HyperPrep Kit. We then generated 192.5 M pairs (58× depth of coverage) of 2×251 bp Illumina TruSeq WGS sequencing reads (NCBI SRA accession codes SRR10427760 [https://www.ncbi.nlm.nih.gov/sra/?term=SRR10427760] and SRR10427761 [https://www.ncbi.nlm.nih.gov/sra/?term=SRR10427761]) on a HiSeq 2500 at the QB3 VCGSL. Additionally, DNAs from pools of frogs contemporaneous with the cross reported in Mitros et al.[4] were extracted from adult toe clippings. Illumina libraries were prepared by Covaris shearing and gel selection for 500 bp insert size followed by adapter annealing using TruSeq protocol V2. Frogs were sequenced as pools of 12 F_{13} Nigerian strain males, 8 F_{13} Nigerian strain females, and 6 F_7 Ivory Coast B (ICB) male frogs on a HiSeq 2500 at the QB3 VCGSL. The sequences are deposited at the NCBI under BioProject PRJNA526297 [https://www.ncbi.nlm.nih.gov/sra/?term=PRJNA526297] with SRA accession codes SRR18189668 [https://www.ncbi.nlm.nih.gov/sra/?term=SRR18189668], SRR18189669 [https://www.ncbi.nlm.nih.gov/sra/?term=SRR18189669], and SRR18189667 [https://www.ncbi.nlm.nih.gov/sra/?term=SRR18189667], respectively.

Supplementary Note 2: Chromosome-scale genome assembly and annotation of *Xenopus tropicalis*.

Genome assembly

De novo and hybrid contig assemblies

Two independent Pacific Biosciences (PacBio) continuous long-read (CLR)-based contig assemblies were constructed; the first contig dataset was assembled *de novo* using Canu[23], and the second using the hybrid genome assembler, DBG2OLC[24].

Canu (v1.6-132-gf9284f8) assembled 1,561 Mb of total sequence, with half of the assembled bases represented in (n = 91) contigs 3.5 Mb or longer. Canu was invoked with the following parameters: *genomeSize=1.7g minReadLength=5000 minOverlapLength=2000*.

DBG2OLC (commit 1f7e752) hybrid contigs were assembled by incorporating 10x Genomics Supernova-assembled contigs (described below) with the raw/uncorrected PacBio CLR sequences. Parameter sweeps to maximize assembly contiguity and total assembled sequence determined that *k 17 KmerCovTh 2 MinOverlap 20 AdaptiveTh 0.005 RemoveChimera 1 MinLen 3000* yielded the optimal assembly for the data. DBG2OLC parameters were varied in all combinations over the following ranges: *KmerCovTh*, 2–10; *MinOverlap*, 10–150; and *AdaptiveTh*, 0.0001–0.02. The optimal contig assembly was polished twice with PBDAGCON[25] (v0.3) using reads aligned by BLASR[26] (v5.3) as input (PBDAGCON params: *-t 0 -c 0*; BLASR params: *--bestn 1 --nproc 8 --minAlnLength 2500 --minPctSimilarity 70*). These contigs represent 1,429 Mb of total assembled sequence, with half of the assembled bases in (n = 174) contigs 2.4 Mb or longer.

The Supernova[27] (v1.1.5) assembly used in the hybrid assembly above was generated by the HudsonAlpha Institute for Biotechnology (HAIB) using default parameters and output in “pseudohaploid1” mode. Of the 380 million Chromium Genome linked read pairs input to Supernova, 35% of the reads passed its internal QC procedures and were used in assembly. This resulted in an effective depth of 30.4× in sequence coverage. Supernova scaffolds were broken into contigs at scaffolding gaps prior to inputting to DBG2OLC. These Supernova-assembled scaffolds represent 1,365 Mb of total sequence, 1,204 Mb of which is captured in contig sequence (N50 length = 18.3 kb, N50 count = 17,057).

The 10x Genomics Chromium Genome linked reads were later reassembled with Supernova (v2.0.1) using default parameters and output in “pseudohaploid1” mode, resulting in a more complete assembly: 1,507 Mb total scaffold sequence and 1,327 Mb total contig sequence, with an N50 length of 20.5 kb (N50 count = 16,433).

Contig meta-assembly

Both *de novo*- and hybrid-assembled contig datasets were used as quickmerge[28] (commit e4ea490) "donor" and "acceptor" inputs in a two-way hierarchical contig merging strategy. This process was motivated by observing complementary overlaps between the contig sets when compared using nucmer[29] (MUMmer v3.23). The merging strategy is illustrated for clarity in **Supplementary Fig. 1a** and described in detail below, where "*D*" and "*H*" represent *de novo* (Canu) and hybrid (DBG2OLC) assemblies, respectively.

Prior to merging, contig extension errors in the initial *D* and *H* contig sets were first broken. These mis-assemblies were detected by performing a preliminary round of scaffolding on each contig set with Juicer[30,31] (commit 94ec691) and 3D-DNA[31,32] (commit 2796c3b), identified via visualization in Juicebox[1] (v1.9.0), and then manually broken with Juicebox Assembly Tools[2] (JBAT). Merging was then performed in two rounds:

Round 1: Corrected *D* and *H* contig sets (*D*' and *H*') were merged (*-hco 16 -c 5 -l 100000 -ml 10000*) using alignments generated by nucmer (default parameters) and filtered with delta-filter[29] (*-q -r -i 95*). However, the outcome of merging is asymmetric and depends on the contig set input order (i.e., their "acceptor" or "donor" status). To exhaust potential merges and maximize the amount of metassembled sequences, reciprocal metassemblies were performed. This resulted in M_{DH} and M_{HD} metassembled contig sets, where subscripts list which contig sets were used as acceptor and donor in each merge, respectively. These initial metassembled contig sets were then corrected for merge errors with JBAT, as was performed above.

Round 2: Correcting the reciprocal metassemblies above created two contig sets, called M'_{DH} and M'_{HD}, each accomplishing a subset of the total potential merges (a result of the asymmetric nature of the quickmerge algorithm). However, the unrealized potential merges in Round 1 could be exploited by a second round of merging to produce a single, optimal metassembly, $M_{HD,DH}$. Finally, this metassembly was corrected of merge errors, as was performed above, generating a final metassembly ($M'_{HD,DH}$) with a contig N50 length of 7.7 Mb (N50 count = 48) and capturing 1,453.4 Mb of the genome sequence.

Removing redundant contig sequences

Upon aligning the whole-genome shotgun (WGS) short-read Illumina sequences to the metassembled contigs, we examined the median depth profiles for each contig (**Supplementary Fig. 1b**) and observed an abundance of contigs with approximately half the depth expected from sequencing, as well as many contigs that have near-zero depth. The latter contigs were discarded as assembly artifacts, while the former were aligned all-vs.-all to the entire metassembled contig set using nucmer[29] (MUMmer v3.23; default parameters). After removing self-mapped alignments, the shorter of two contigs that aligned over at least 90% of their length at 90% identity (as these were yet-unpolished sequences) or greater were discarded (redund-contigs v0.1.0, https://bitbucket.org/bredeson/artisanal). This process was repeated until half-depth sequences could no longer be purged from the contig set. If a half-depth sequence could be identified as redundant to another, larger contig in Juicebox[1], it was also

removed. The final non-redundant assembly converged to 1,453 Mb, with 127 Mb of duplicate/repetitive sequences removed.

Recovering genic contigs

Because the process for removing redundant sequences was aggressive, it had the potential to remove legitimate contigs that partially overlapped (i.e., were partially redundant with) another, larger contig, yet still contained uniquely-assembled sequence. To assess the degree to which this happened and recover lost sequences, we aligned all 52,323 *X. tropicalis* transcript CDS sequences available at XenBase (http://ftp.xenbase.org/pub/Genomics/JGI/Xentr9.1) to the filtered metassembled contigs using GMAP[33] (version 2019-03-15; *--npaths=0 --min-identity=90 --format=gff3_match_cdna*) and Minimap2[34] (v2.5-284-g1739a26; *-c -x splice --secondary=no*). Of those sequences, 131 were found to be mitochondrial or other contaminants and were excluded from further analysis. Among the 52,192 remaining CDS sequences, only 356 (0.68%) could not be found with minimum thresholds of 90% identity and 50% sequence coverage. These unmapped sequences were then used to probe (in the following order) the Canu, DBG2OLC, Supernova (v1.1.5, then v2.0.1), v9, and v4 assemblies to recover gene-containing contigs that were excluded previously or that were not assembled at all. In total, 315 CDS sequences were recovered and 41 (0.079%) could not be (see “Evaluating assembly completeness and correctness” section below).

Mate-pair, Hi-C, and synteny-based scaffolding

Merged and error-corrected contigs from the metassembly procedure were scaffolded using SSPACE[35] v3.0 with 3 kb, 8 kb, and 40 kb mate-pair libraries and 140 kb BAC-end libraries[4]. These reads were downloaded from the NCBI Trace Archive (query: *SPECIES_CODE = "XENOPUS TROPICALIS" and CENTER_NAME = "JGI" and SOURCE_TYPE = "GENOMIC"*) and their 5’ ends trimmed to remove low-quality (baseQ < 20) bases. Reads were then truncated to a maximum length of 750 bases and aligned to the metassembly with BWA-MEM[36] (v0.7.17-r1188) independently as single-end reads. Read alignments were then paired and filtered for proper pair orientation and insert distance (3 kb, 8 kb, 40 kb libraries, within 6 SDs from the mean; 140 kb BAC-ends, within 4 SDs from the mean) using custom scripts (repair and scaffold-read-filter.py v0.1.0, https://bitbucket.org/bredeson/artisanal). Pairs with mapping quality (MQ) ≥ 30 were converted to TAB format and used for scaffolding with SSPACE v3.0 (*-Z 6 -k 2 -n 1e12*).

Mate-pair scaffolded contigs were ordered and oriented into chromosomal scaffolds with 3D-DNA[31,32] (commit 2796c3b) using 108.9M Hi-C contacts (MQ ≥ 1) extracted by Juicer[30,32] (commit d3ee11b). The Hi-C scaffolded assembly was manually curated in the Juicebox[1] (v1.9.0) visualizer with JBAT[2] to optimize the adjacency of high-density inter-contig contacts along the main diagonal. This was performed by identifying and correcting the Hi-C contact patterns created by scaffolding errors, such as those described by Dudchenko et al.[2]. Additionally, contigs not placed into scaffolds by 3D-DNA were scaffolded manually if sufficient linking signal was observed.

Synteny with a preliminary version of a PacBio- and Hi-C-based *X. laevis* assembly (NCBI GenBank accession GCF_017654675.1 [https://www.ncbi.nlm.nih.gov/datasets/genome/GCF_017654675.1]) was used to refine ambiguities in contig order and orientation, particularly within subtelomeric regions of the *X. tropicalis* assembly, where contigs tended to be small and the Hi-C contact densities may have been too sparse or signal-to-noise too low. Contigs were aligned using nucmer (MUMmer v3.23) with default parameters, filtered using delta-filter (*-q -r -o 10*), then visualized using mummerplot. Contigs were ordered and oriented along the chromosomes using layout files (via *-Q* and *-R* options). Of the 20 chromosome ends, four telomeric sequences were captured in the assembly, and are capped by (TTAGGG)n repeats. The final chromosome-scale scaffolding is presented in **Supplementary Fig. 1e**.

Gap closing

Gaps introduced by Hi-C scaffolding are of unknown size and were assigned a fixed length of 1,000 bp. A fixed-length gap was re-sized if it could be spanned by two or more Sanger mate pairs (aligned as described in the previous section), with both ends in a pair with mapping quality 30 or greater. Gaps were then re-sized using a custom script (mpGapLen v0.1.0, https://bitbucket.org/bredeson/artisanal; *-q1 -k3 -S -Z6*). Given the orders and orientations of contigs within chromosomal scaffolds, we were able to resolve with greater specificity overlaps between segments of chromosome created by premature contig extension termination. These overlaps constitute gaps of negative length in the assembly. For a potential negative gap between two contigs to be closeable, contigs were required to overlap by at least 1,000 bp and align at 95% sequence identity; the shorter of the two overlapping sequences was then trimmed and the two contigs concatenated together. This procedure closed 80 of 1,248 gaps in the chromosomes. To close positive gaps, we performed two rounds of PBJelly[37] (PBSuite v15.8.24) gap filling, re-sizing gaps after each round as described above, and closed 478 gaps. An additional six gaps were closed via polishing with Pilon (see next section).

Assembly Polishing

Canu and DBG2OLC assemblies were evaluated for short-range structural correctness with custom assembly error detection software called WOMBAT v0.1.11 (https://gitlab.com/Bredeson/wombat, parameters: *-A3 -a1 -m10000 -l10000 -W2000 -w100 -S500 -s50*) using PacBio read alignments. Common errors identified included collapsed multi-copy sequences and artifactual sequences introduced by the DBG2OLC assembly process (**Supplementary Fig. 1c**). Sequences identified to be artifactual were hard-masked with Ns and the Canu contig sequences aligned to them with nucmer[29] (MUMmer v3.23). Canu contigs completely spanning artifacts with flanking alignments 10 kb or longer were used to fill hard-masked regions using custom Python scripts (assembly-patch-finder and assembly-patch-patcher v0.1.0, https://bitbucket.org/bredeson/artisanal).

Long-read, signal-based polishing was performed twice on the final scaffolds with the Arrow[25] polishing tool from the SMRTlink (v5.0.0.6792) software suite. BLASR[26] (v5.3) was used to perform the read alignments (*--algorithm arrow --fancyChunking --skipUnrecognizedContigs --*

noEvidenceConsensusCall lowercasereference --refineDinucleotideRepeats --reportEffectiveCoverage).

Illumina-based polishing was performed using 2×251 bp (SRR10427760 [https://www.ncbi.nlm.nih.gov/sra/?term=SRR10427760] and SRR10427761 [https://www.ncbi.nlm.nih.gov/sra/?term=SRR10427761]) WGS sequences and Pilon[38] (v1.23; --*fix all --minqual 20 --mindepth 10 --minmq 20 --diploid*). Manual inspection of read alignments to the Pilon-polished scaffolds, however, revealed errors persisted (24.4/Mb) in the assembly. This prompted us to devise and implement the following custom polishing procedure: Reads were aligned using BWA-MEM[36] (v0.7.17-r1188) and filtered for proper pairing using SAMtools[39] (v1.6). Variants were then called using FreeBayes[40] (v1.1.0-54-g49413aa; *--use-mapping-quality --genotype-qualities --report-genotype-likelihood-max --min-alternate-count 2 --min-alternate-fraction 0.05 --min-base-quality 20 --min-mapping-quality 20 --min-repeat-entropy 1 --max-complex-gap -1 --strict-vcf*) and homozygous variants (SNVs and indels) within the depth range 10–80× were selected. Furthermore, because the predominant error type inherent to long-read sequencing data are indels, and because the Arrow consensus algorithm does not fully support diploid datasets, heterozygous multi-nucleotide indel variants in the depth range 10–80× were also selected to allow incorporating heterozygous loci that may have been excluded by the Arrow consensus procedure. These selected variants were patched into the contigs using a custom Python script called ILEC (v0.1.3, https://bitbucket.org/rokhsar-lab/map4cns). This procedure was iterated (6 times) until the number of frameshift errors observed in the coding regions converged.

Evaluating assembly completeness and correctness

The sequence length placed in chromosome-scale scaffolds (1,449,319,640 bp) is 99.86% of the assembly, while the remaining unplaced sequences (1,963,959 bp) are distributed in 156 scaffolds. The assembly comparisons between the v9 sequence[4] and this new *X. tropicalis* v10 are shown in **Supplementary Tables 1** and **2**. To evaluate assembly completeness, we measured the fraction of Illumina reads (n = 385,527,636) that mapped to the assembled sequence. We were able to map 99.74% of the read total aligned, of these 96.57% were properly paired (**Supplementary Table 1**).

The comparison of the mapping of the 52,323 full-length *X. tropicalis* transcript predictions show that the current version recovered the sequences of genes that were missing from the previous assembly (**Supplementary Fig. 2d,e**, **Supplementary Table 2**). To determine the genic completeness of this assembly we utilized two sets of *X. tropicalis* transcriptomes obtained from Sanger data and independent from other genome annotations. We aligned the 8,558 full-insert cDNA sequences from the Mammalian Gene Collection (which, despite its name, includes non-mammalian species) using exonerate v2.4.0 (model: coding2genome) (**Supplementary Table 2**). A total of 119 frameshifts were identified in 60 cDNA alignments that spanned 11,423,049 bp. Further inspection of the sequences with indels revealed that the substantial majority of these were caused by Sanger sequencing errors, rather than errors in the assembly. The second estimate of gene set completeness was assessed by BUSCO[13–15] v3.0.2-11-g1554283 pipeline run using the OrthoDB tetrapoda dataset (n = 3,950). Although the BUSCO

completeness estimate for the genome sequence is low (91.7%), we find 99.4% of known *X. tropicalis* genes from the Mammalian Gene Collection[41] are present in the assembly and 97.0% of BUSCOs among its predicted proteins, attesting to its substantial completeness (**Supplementary Table 2**).

Two examples of fixed gene annotation predictions with respect to the previous assembly are *dnai1* and *atp4a* (**Supplementary Fig. 2d,e**). In these cases, some of the exons of the genes lacked Sanger read support and were surrounded by tandem repeats. One case of broken gene prediction corresponds to *myod1,* a locus also surrounded by large stretches of tandem repeats and low-quality sequence (**Supplementary Fig. 2f).** These artifacts were not able to be resolved, possibly due to the limitation of library insert size and the high complexity of the region caused by tandem arrays. In the current assembly most of the gaps are in the subtelomeres (**Supplementary Fig. 2**), as these are packed with larger tandem arrays and have a high GC content compared to the inner portions of the chromosomes (**Supplementary Fig. 2g**).

Estimating the genome size and residual heterozygosity of *Xenopus tropicalis*

Prior to estimating the genome size of *X. tropicalis* using a k-mer counting approach, positively identified contaminants were removed from the short-insert Illumina shotgun sequencing reads (SRR10427760 [https://www.ncbi.nlm.nih.gov/sra/?term=SRR10427760] and SRR10427761 [https://www.ncbi.nlm.nih.gov/sra/?term=SRR10427761]). To reduce the amount of sequence to search databases for contaminants, Illumina reads were first aligned to the v10 genome sequence. Read pairs that did not map properly or did not map at all against the nuclear genome were aligned against 1) the complete *X. tropicalis* mitochondrial genome and 2) the NCBI NT database (downloaded 2019-01-22) using BLASTN[42] (BLAST+ v2.9.0; *-num_threads 4 -evalue 1e-3 -num_alignments 1*). Pairs containing at least one read with a best-hit to a non-vertebrate species or to the mitochondrial genome were discarded (129,575 pairs). Likewise, any pair with at least one read aligned for 90% of its length at 99% identity to a non-*Xenopus* vertebrate sequence was also discarded (627 pairs). Counted k-mers reached a maximum frequency of one million.

Non-contaminant reads were then k-mer counted canonically using jellyfish[43] (v2.2.10); where k = 51. The resulting 51-mer histogram was analyzed to estimate the genome size with GenomeScope[44] (v1.0.0-6-gd2aefdd). The genome size of *X. tropicalis* was estimated to be 1.452 Gb, with 0.336 Gb (23.1%) of that determined to be captured in repetitive elements (**Supplementary Fig. 1d**).

The reference individual used for sequencing was an F_{17} female derived from the same Nigerian line used to produce the previous v4 and v9 assemblies[4,45]. To assess the degree of residual heterozygosity potentially latent in the inbred reference strain, we used BWA-MEM[36] v0.7.17-r1188 to align the 2×251 bp paired-end WGS sequencing reads (SRR10427760 [https://www.ncbi.nlm.nih.gov/sra/?term=SRR10427760] and SRR10427761 [https://www.ncbi.nlm.nih.gov/sra/?term=SRR10427761]) to the v10 assembly, then filtered for

proper read-pairing (pair orientation and distance) and variants called with FreeBayes[40] (v1.1.0-54-g49413aa; parameters: *--genotype-qualities --use-mapping-quality --report-genotype-likelihood-max --min-base-quality 20 --min-mapping-quality 30 --haplotype-length 1 --use-best-n-alleles 4 --strict-vcf --report-monomorphic*). Multi-allelic variants and indels were discarded, and bi-allelic SNVs were filtered for depth of coverage between □ $\pm$ 1.78σ and allele balance between 0.3 and 0.7, inclusive, to mitigate false positive variant calls caused by low mappability and/or genomic repeats. Rates of heterozygous loci and loci homozygous for non-reference alleles were estimated[46] using 500-kb sliding windows every 50 kb along each chromosome. This approach reveals that this F_{17} Nigerian strain female retains blocks of heterozygosity in 14.1% of its genome (**Supplementary Fig. 6**). The average heterozygosity within these blocks is estimated as 2.96×10^{-3}, consistent with previous estimates within this species[47]. To expand the catalog SNP variation among *X. tropicalis* strains, the above methods were repeated using the reads from pools of 12 F_{13} Nigerian strain males (NCBI SRA accession SRR18189668 [https://www.ncbi.nlm.nih.gov/sra/?term=SRR18189668]), 8 F_{13} Nigerian strain females (NCBI SRA accession SRR18189669 [https://www.ncbi.nlm.nih.gov/sra/?term=SRR18189669]), and 6 F_7 Ivory Coast B (ICB) male frogs (NCBI SRA accession SRR18189667 [https://www.ncbi.nlm.nih.gov/sra/?term=SRR18189667]).

Genome annotation

Repeat annotation and estimation of sequence divergence

RepeatModeler[48] v1.0.11 was run on an intermediate, unpolished version of the assembled *X. tropicalis* contigs. Identified repeats were manually curated to exclude false positives and recover false negatives, leading to a total of 973 repeats. Repeats from seven other anuran genome assemblies (*A. truei* [n = 1,769], *E. coqui* [n = 1,441], *E. pustulosus* [n = 1,146], *H. boettgeri* [n = 1,160], *P. adspersus* [n = 908], *X. borealis* [n = 1,026], and *X. laevis* [n = 913]) were also identified and included into a pan-anuran repeat library. We combined the frog and ancestral RepBase[49] v23.12 dataset (n = 934) with the curated repeats above to create the final repeat library (n = 10,270). The curated repeat library was subsequently utilized as input for RepeatMasker[50] v4.0.7 to annotate repeats on *Xenopus tropicalis* v10. A corrected measurement of sequence divergence was obtained by applying the Jukes-Cantor model to the substitution rate of the sequence calculated by RepeatMasker with respect to the consensus repeat family obtained by RepeatModeler and the RepBase database. The repeat annotation using RepeatMasker found that 40.12% of the *X. tropicalis* genome is covered by repeats (**Supplementary Table 7**). Tandem Repeats Finder[51] v4.09 was used to identify all tandem repeats in the chromosomes (**Methods**). A total of 154.75 Mb in the genome are covered by tandem arrays consisting of > 5 consecutive monomers that are greater than 10 bp long (**Supplementary Fig. 2**).

Selection of ESTs and valid transcript models for genome annotation

We obtained 1,271,375 *Xenopus tropicalis* EST sequences from the NCBI Nucleotide database (query: "Xenopus tropicalis"[Organism] AND is_est[filter]). ESTs sequenced from the forward and reverse orientations were assembled using PEAR[52] v0.9.8. Successfully assembled ESTs

and single ESTs with a minimum sequence length of 250 bp were mapped against the v10 genome assembly using STARlong[53] v2.7.0e. Stranded RNA-seq data from adult tissues[54] and non-stranded RNA-seq data from different developmental stages[55] were obtained from the Sequence Read Archive (SRA). We only considered samples that were poly-A selected. We pooled samples from equivalent stages or tissue types to increase sequencing read depth (9 stages: cleavage, blastula, gastrula_early, gastrula_late, neurula_early, neurula_mid, neurula_late, tadpole_early, tadpole_mid, tadpole_late; 6 adult tissues: heart, testis, brain, liver, ovary, kidney. **Supplementary Data 1**). The RNA-seq reads were aligned against the unmasked version of the *Xenopus tropicalis* genome assembly v10 using STAR[53] v2.7.0e. Alignments became the inputs for the Trinity transcriptome assembler[56,57] v2.5.1. We obtained a total of 2.4 billion transcript assemblies (TAs), which were subsequently evaluated after being mapped to the genome using STARlong. The EST and TA predictions mapped by STARlong were used to evaluate the quality of the TAs. To prevent fused TAs we required that the read coverage spanning splice junctions remained consistent along the transcript. TAs and ESTs were also discarded if they presented abnormal mapping features (e.g., low mapping quality, short intron lengths, short first or last exon lengths).

Protein-coding gene annotation

X. tropicalis clones (n = 8,980) from the Mammalian Gene Collection[41] (MGC) were used as the set of confident and full-length mRNA evidence. MGC clones from *X. laevis* (n = 11,515) were utilized as sister transcripts. Human, mouse, chicken, and zebrafish proteins were used for peptide homology evidence. Several rounds of genome annotation and evaluation were implemented to assess the completeness of the gene predictions. Filtered EST and TAs were used as mRNA evidence by the DOE-JGI Integrated Gene Call[16] (IGC) v5.0 pipeline for genome annotation (**Supplementary Table 4**).

Supplementary Note 3: Chromosome-scale genome assembly and annotation of additional frogs.

Sequencing

Genome and transcriptome sequencing

All sequencing data have been deposited in the NCBI SRA and are summarized in **Supplementary Data 1**.

DNA extraction and sequencing of *E. pustulosus*.

High molecular weight DNA was extracted, as previously described[7], from whole blood from two sisters (237g6f4 and 237g6f5) maintained at the University of the Pacific. Using DNA from one sister (237g6f4), a 10x Genomics Chromium Genome library[27] was prepared and sequenced on the Illumina HiSeq X by the HudsonAlpha Institute for Biotechnology (HAIB). Using DNA from the other sister (237g6f5), Pacific Biosciences (PacBio) Single-Molecule Real-Time (SMRT) continuous long-read (CLR) libraries were prepared and sequenced on the PacBio Sequel I by the DNA Technologies and Expression Analysis Cores at the University of California Davis Genome Center. Using liver dissected from a niece of the sisters (291g2f_3603 also coded 291g2f3), also maintained at the University of the Pacific, a Hi-C library was prepared using the Dovetail Genomics Hi-C library preparation kit and sequenced on the Illumina HiSeq 4000 by the QB3 Vincent J. Coates Genomics Sequencing Laboratory (VGCSL) at the University of California, Berkeley (UCB). Two additional Hi-C libraries were prepared from the dissected liver and sequenced on the Illumina NextSeq by Dovetail Genomics.

DNA extraction and sequencing, *H. boettgeri*.

High molecular weight DNA was extracted, as previously described[7], from whole blood from one female (F_2) purchased at the Albany Aquarium. A 10x Genomics Chromium Genome paired-end linked-read library[27] was prepared and sequenced on the Illumina HiSeq X by the HAIB. PacBio SMRT CLR libraries were prepared and sequenced on the PacBio Sequel I by the HAIB. Using liver dissected from a second, unrelated female (F_3) purchased at the Albany Aquarium, a Hi-C library was prepared using the Dovetail Genomics Hi-C library preparation kit and sequenced on the Illumina HiSeq 4000 by the VCGSL at UCB.

Additional sequencing of *H. boettgeri*.

DNA was extracted, as previously described[7], from whole blood from a female (F_1) purchased at the Albany Aquarium. A short insert library was prepared using the Takara PrepX DNA Library Kit by the QB3 Functional Genomics Laboratory (FGL) at UCB and sequenced on the Illumina HiSeq 2500 by the VCGSL at UCB.

DNA extraction and sequencing of *E. coqui*.

Kidney and liver tissue were dissected from one male collected in Hawaii (HN-11 male), and DNA was extracted from these tissues using the Zymo Research Quick gDNA MiniPrep Kit (cat# D3007). Two short insert libraries were prepared using the Takara PrepX DNA Library Kit with the kidney DNA by the FGL at UCB and sequenced on the Illumina HiSeq 2500 and 4000 by the VCGSL at UCB. Two mate-pair libraries were prepared using liver DNA and sequenced on the Illumina HiSeq 2500 by the HAIB. Using the liver tissue sample, a Hi-C library was prepared and sequenced on the Illumina NextSeq by Dovetail Genomics. High molecular weight DNA was extracted, as previously described[7], from whole blood from a second, unrelated male (C4M) maintained at Harvard University. Using DNA from this second male, a 10x Genomics Chromium Genome library[27] was prepared and sequenced on the Illumina HiSeq X by the HAIB.

Additional sequencing of *E. coqui*.

Liver tissue was dissected from one female collected in Hawaii (HN-13 female), and DNA was extracted from the tissue using the Zymo Research Quick gDNA MiniPrep kit (cat# D3007). Two short insert libraries were prepared using the Takara PrepX DNA Library Kit by the FGL at UCB and sequenced on the Illumina HiSeq 2500 and 4000 by the VCGSL at UCB.

RNA extraction and sequencing of *E. pustulosus*.

In addition to the two whole tadpoles (excluding gut) at approximated stages 45 and 56, the following tissues were dissected from adult frogs maintained at the University of the Pacific: brain (n = 3), dorsal skin (n = 2), eggs (n = 2), eye (n = 2), heart (n = 2), intestine (n = 2), larynx (n = 3), liver (n = 2), lung (n = 2), and ventral skin (n = 2). All samples were washed twice with PBS, homogenized in TRIzol Reagent, and centrifuged, followed by flash freezing of the supernatant. RNA was isolated following the *TRIzol Reagent User Guide* (Pub. No. MAN0001271 Rev. A.0) protocol. Illumina mRNA libraries were prepared using the Illumina TruSeq Stranded mRNA Library Prep Kit and sequenced on the Illumina HiSeq 4000 by the VCGSL at UCB.

RNA extraction and sequencing of *H. boettgeri*.

Eggs were homogenized in TRIzol Reagent and processed according to the manufacturer's instructions. RNA was then isolated using the QIAGEN RNeasy Mini Kit (cat# 74104). An Illumina mRNA library was prepared using the Takara PrepX RNA-Seq for Illumina Library Kit by the FGL at UCB and sequenced on the Illumina HiSeq 4000 by the VCGSL at UCB.

Genome assembly

The assembly of *E. coqui, E. pustulosus*, and *H. boettgeri*, detailed below, followed a hierarchical strategy, starting with short-read data and progressing to longer reads and linkages. Hi-C sequence libraries were prepared and sequenced as described in **Methods**.

Shotgun assembly of *E. pustulosus*

10x Genomics Chromium Genome paired-end linked reads were assembled with Supernova[27] (v2.0.1). As previously described[58], putative archaeal, bacterial, viral, and vector contaminants were identified and removed by querying the assembly using BLAST+[42] (v2.6.0) against the respective NCBI RefSeq and UniVec databases, using general_decon.sh[58] (v1.0, https://github.com/abmudd/Assembly). Putative mitochondrial sequences were also identified and removed by querying the assembly using BLAST+ (v2.6.0) against the closest available mitochondrial assembly[59] (NCBI GenBank accession JX564888.1 [https://www.ncbi.nlm.nih.gov/nuccore/JX564888.1]), using mt_decon.sh[58] (v1.0, https://github.com/abmudd/Assembly). Finally, putative nonvertebrate contamination was identified and removed by two rounds of filtering, using custom script nt_decon.sh (v1.0, https://github.com/abmudd/Assembly): (1) the assembly was queried using BLAST+ (v2.6.0) against the NCBI NT database, flagging sequences with an E-value less than 1×10^{-10} best hit to a nonvertebrate sequence, as identified by the corresponding taxonomic information; (2) flagged sequences were queried using BLAST+ (v2.6.0) against previously published frog genomes (*Hyla arborea*[60], *Nanorana parkeri*[61,62], *P. adspersus*[63] (v29Jun2017), *Rana catesbeiana*[64] (v3-20170621), *R. temporaria*[65,66], *X. laevis*[7] (GCA_001663975.1 [https://www.ncbi.nlm.nih.gov/datasets/genome/GCA_001663975.1]), and *X. tropicalis*[4] (GCA_000004195.3 [https://www.ncbi.nlm.nih.gov/datasets/genome/GCA_000004195.3])) as well as frog sequences from NCBI EST, GSS, and nucleotide databases, removing sequences without any hits based on a cutoff of 75% identity and an E-value less than 1×10^{-10}. The decontamination removed 8,581 scaffolds totaling 2.11 Mb from the Supernova assembly.

Initial PacBio scaffolding of *E. pustulosus*

To improve the contiguity of the *E. pustulosus* assembly, decontaminated Supernova contigs were scaffolded with PacBio CLR data. This was achieved by performing a hybrid assembly of the filtered Supernova contigs and PacBio reads using DBG2OLC[24] (commit 1f7e752). PacBio reads were then mapped to the DBG2OLC output assembly with BLASR[26] (commit 4323a52) and polished with PBDAGCON[25] (commit 1a2f1e7) two times, using the map4cns pipeline (commit dd89f52, https://bitbucket.org/rokhsar-lab/map4cns). Decontaminated Supernova contigs were mapped back to the polished DBG2OLC assembly using MUMmer[29] (v3.23) with a cutoff of 90% identity and then ordered and oriented into scaffolds using tsvtk and maptk from the GBS analysis pipeline[67] (commit 80613d5, https://bitbucket.org/rokhsar-lab/gbs-analysis).

Initial chromosome assembly of *E. pustulosus*

After PacBio-based CLR scaffolding, the assembly was organized into chromosomes with Hi-C data using the Dovetail Genomics HiRise pipeline[68]. Hi-C reads were aligned to the assembly with Juicer[30,31] (commit d3ee11b), then the assembly was manually corrected in Juicebox[1] (v1.9.0) with Juicebox Assembly Tools[2]. PacBio reads were aligned to the assembly with BWA[36] (v0.7.17-r1188) and gaps were resized using custom scripts (pbGapLen v0.0.2, https://bitbucket.org/rokhsar-lab/xentr10/src/master/assembly; expand-gaps.py v0.1.0, https://bitbucket.org/bredeson/artisanal). Gaps in the assembly were then filled with PacBio data using PBJelly[37] (PBSuite v15.8.24).

Revised PacBio and 10x Genomics scaffolding of *E. pustulosus*

Given the limited resolution of the *E. pustulosus* Hi-C data for determining the proper orders and orientations of scaffolds as well as the large number of gaps listed as overfilled by PBJelly—suggesting incorrect scaffolding, rearrangements, or other assembly errors—the gap-filled assembly was broken into contigs and then scaffolded with PacBio CLR and 10x Genomics data. First, the contigs were scaffolded against the error-corrected DBG2OLC assembly using MUMmer[29] (v3.23) as well as tsvtk and maptk in the GBS analysis pipeline[67] (commit 80613d5, https://bitbucket.org/rokhsar-lab/gbs-analysis). Next, the assembly was scaffolded with the 10x Genomics Chromium Genome linked reads using Scaff10X (v2.1, https://sourceforge.net/projects/phusion2/files/scaff10x). Gaps were resized with PacBio CLR data, as described above, and filled with PBJelly[37] (PBSuite v15.8.24).

Revised chromosome assembly of *E. pustulosus*

The resulting assembly was organized back into chromosomes based on alignment against the initial chromosome assembly using MUMmer[29] (v3.23) as well as tsvtk and maptk in the GBS analysis pipeline[67] (commit 80613d5, https://bitbucket.org/rokhsar-lab/gbs-analysis). Gaps were again resized with PacBio data using custom scripts (pbGapLen v0.0.2, https://bitbucket.org/rokhsar-lab/xentr10/src/master/assembly; expand-gaps.py v0.1.0, https://bitbucket.org/bredeson/artisanal), and gaps were filled with PBJelly[37] (PBSuite v15.8.24).

Final assembly correction of *E. pustulosus*

The assembly was polished with two rounds of Illumina error correction. In this, 10x Genomics data were adapter trimmed using trim_10X.py[58] (v1.0, https://github.com/abmudd/Assembly) and aligned to the assembly with BWA[36] (v0.7.17-r1188). Variants called by FreeBayes[40] (commit 49413aa) with a read depth within two standard deviations of the Gaussian fit (mean of 26.4 and standard deviation of 10.4) were corrected using the script ILEC in the map4cns pipeline (commit dd89f52, https://bitbucket.org/rokhsar-lab/map4cns).

After error correction, the Hi-C data were realigned to the assembly with Juicer[30] (commit d3ee11b). Misjoins were identified and broken in Juicebox[1] (v1.9.0) with JBAT[2]. The remaining gaps were resized with PacBio CLR data using BWA[36] (v0.7.17-r1188) and custom scripts (pbGapLen v0.0.2, https://bitbucket.org/rokhsar-lab/xentr10/src/master/assembly; expand-gaps.py v0.1.0, https://bitbucket.org/bredeson/artisanal), then closure was attempted with the adapter-trimmed 10x Genomics Chromium Genome paired-end reads using Platanus[69] (v1.2.1).

Final assembly release of *E. pustulosus*

Scaffolds smaller than one kb were removed from the final assembly with seqtk (v1.3-r106, https://github.com/lh3/seqtk), and chromosomes and scaffolds were numbered in order of size using SeqKit[70] (v0.7.2-dev). Chromosomes were oriented arbitrarily.

Assembly of *H. boettgeri*

The assembly process followed the same procedure as outlined for *E. pustulosus* with four differences: the closest available mitochondrial assembly[71] used in decontamination was NCBI GenBank sequence NC_015615.1 [https://www.ncbi.nlm.nih.gov/nuccore/NC_015615.1]; the decontamination removed 108 scaffolds totaling 39.9 kb from the Supernova assembly; variants with a read depth within only one standard deviation of the Gaussian fit (mean of 18.4 and standard deviation of 12.3) were corrected; and chromosomes were numbered and oriented based on alignment with MUMmer[29] (v3.23) to the *X. tropicalis* chromosomes.

Shotgun assembly of *E. coqui*

The short insert libraries were adapter trimmed with ea-utils[72] fastq-mcf (commit bd148d4). The mate-pair libraries were adapter trimmed and split with NxTrim[73] (commit 53c2193). Using custom script nxtrim_pipeline.sh (v1.0, https://github.com/abmudd/Assembly), the output from NxTrim was divided into two files: (1) reads flagged as mate pair or unknown were merged into a final mate pair file; (2) reads flagged as short insert paired-end or single-end were merged into a final short insert library, with the single-end reads given a corresponding blank second end. All trimmed data were then assembled with Meraculous[74,75] (v2.2.4). Mitochondrial sequence was assembled from adapter-trimmed short insert data using custom script organelle_pipeline.py (v1.0, https://github.com/abmudd/Assembly) and NOVOPlasty[76] (v2.6.3), with other Hyloidea mitochondrial assemblies available on NCBI as input seeds.

As for the *E. pustulosus* shotgun assembly process—and as previously described[58]—putative archaeal, bacterial, viral, and vector contaminants were identified and removed by querying the assembly with BLAST+[42] (v2.3.0) against the respective NCBI RefSeq and UniVec databases, using general_decon.sh[58] (v1.0). Putative mitochondrial sequence was also identified and removed by querying the assembly with BLAST+ (v2.3.0) against the assembled mitochondrial sequence, using mt_decon.sh[58] (v1.0). In addition, the assembly was queried with BLAST+ (v2.3.0) against the NCBI NT database, previously published frog genomes (*H. arborea*[60], *N. parkeri*[61,62], *P. adspersus*[63] (v29Jun2017), *R. catesbeiana*[64] (v3-20170621), *R. temporaria*[65,66], *X. laevis*[7] (GCA_001663975.1 [https://www.ncbi.nlm.nih.gov/datasets/genome/GCA_001663975.1]), and *X. tropicalis*[4] (GCA_000004195.3 [https://www.ncbi.nlm.nih.gov/datasets/genome/GCA_000004195.3])), and frog sequences from NCBI EST, GSS, and nucleotide databases to identify and remove non-vertebrate sequences, using custom script nt_decon.sh (v1.0, https://github.com/abmudd/Assembly). The decontamination removed 7,272 scaffolds totaling 2.06 Mb from the meraculous assembly.

Residual redundancy due to split haplotypes was identified and removed using the custom script align_pipeline.sh (v1.0, https://github.com/abmudd/Assembly). To summarize, the adapter-trimmed libraries were aligned to the assembly with BWA[36] (v0.7.15-r1140). Read depth was extracted from the alignments and used as a cutoff to separate half-depth and full-depth scaffolds. Half-depth scaffolds were then queried against each other with BLAST+ (v2.3.0), and the smaller of each best-hit scaffold pair was extracted. Half-depth scaffolds were also k-mer

counted with Jellyfish[43] (v2.1.4), and the smaller of each scaffold pair with a unique, shared 31-mer was extracted. Scaffolds identified in both BLAST+ and Jellyfish analyses were removed from the assembly. The redundancy pipeline removed 192,996 scaffolds totaling 31.1 Mb from the decontaminated assembly. The assembly was then scaffolded with SSPACE[35] (v3.0).

Chromosome assembly of *E. coqui*

The assembly was next scaffolded with 10x Genomics Chromium Genome linked reads using Scaff10X (v2.1, https://sourceforge.net/projects/phusion2/files/scaff10x). Attempts to further scaffold the assembly into chromosomes with the Hi-C data using the Dovetail Genomics HiRise pipeline[68] and 3D-DNA[31,32] (commit 745779b) were unsuccessful. Therefore, the Scaff10X output was mapped to the *E. pustulosus* assembly using MUMmer[29] (v3.23) and then scaffolded based on synteny using tsvtk and maptk from the GBS analysis pipeline[67] (commit 80613d5, https://bitbucket.org/rokhsar-lab/gbs-analysis). Hi-C reads were aligned to the assembly with Juicer[30,31] (commit d3ee11b), and the synteny-based scaffolding was manually corrected in Juicebox[1] (v1.9.0) with JBAT[2]. Closure of the remaining gaps was attempted with the adapter-trimmed short insert data using Platanus[69] (v1.2.1).

Final assembly release of *E. coqui*

Scaffolds smaller than one kb were removed from the final assembly with seqtk (v1.3-r106, https://github.com/lh3/seqtk), and chromosomes and scaffolds were numbered in order of size using SeqKit[70] (v0.7.2-dev). Chromosomes were oriented arbitrarily.

Correction of published genomes

Reassembly of *L. ailaonicum*

As described for *E. pustulosus*, putative archaeal, bacterial, viral, and vector contamination was checked by querying the published assembly[77,78] using BLAST+[42] (v2.9.0) against the respective NCBI RefSeq and UniVec databases. Putative mitochondrial sequences were also checked by querying the assembly using BLAST+ (v2.9.0) against the closest available mitochondrial assembly[79] (NCBI GenBank accession NC_024427.1 [https://www.ncbi.nlm.nih.gov/nuccore/NC_024427.1]). No contaminant scaffolds were identified or removed from the assembly.

Hi-C reads[77] (BioProject PRJNA523649 [https://www.ncbi.nlm.nih.gov/sra/?term=PRJNA523649]) were aligned to the assembly with Juicer[30] (commit d3ee11b), and the existing scaffolding was manually error corrected in Juicebox[1] (v1.11.08) with JBAT[2]. All gaps were then resized to 100 bp. Chromosomes and scaffolds were numbered in order of size using SeqKit[70] (v0.7.2-dev). Chromosomes were oriented arbitrarily.

Reassembly of *P. adspersus*

As described for *E. pustulosus*, putative archaeal, bacterial, viral, and vector contamination was identified and removed by querying a precursor (v29Jun2017) of the released assembly[63] (GCA_004786255.1 [https://www.ncbi.nlm.nih.gov/datasets/genome/GCA_004786255.1]) using BLAST+ (v2.6.0) against the respective NCBI RefSeq and UniVec databases. Putative mitochondrial sequences were also identified and removed by querying the assembly using BLAST+ (v2.6.0) against the closest available mitochondrial assembly[59] (NCBI GenBank accession JX564898.1 [https://www.ncbi.nlm.nih.gov/nuccore/JX564898.1]). The decontamination removed one scaffold totaling 1.45 kb from the assembly.

Dovetail Chicago and Hi-C reads[63] (BioProject PRJNA439445 [https://www.ncbi.nlm.nih.gov/sra/?term=PRJNA439445]) were aligned to the published assembly with Juicer[30,31] (commit d3ee11b) and errors in the scaffolding were manually corrected in Juicebox[1] (v1.9.0) with JBAT[2]. PacBio reads[63] (BioProject PRJNA439445 [https://www.ncbi.nlm.nih.gov/sra/?term=PRJNA439445]) were aligned to the assembly with BWA[36] (v0.7.17-r1188), and gaps were resized using custom scripts (pbGapLen v0.0.2, https://bitbucket.org/rokhsar-lab/xentr10/src/master/assembly; expand-gaps.py v0.1.0, https://bitbucket.org/bredeson/artisanal). Closure of the remaining gaps was attempted with Platanus[69] (v1.2.1) using Illumina TruSeq data[63] (BioProject PRJNA439445 [https://www.ncbi.nlm.nih.gov/sra/?term=PRJNA439445]) adapter-trimmed with ea-utils[72] fastq-mcf (commit bd148d4).

Scaffolds smaller than one kb were removed from the final assembly with seqtk (v1.3-r106, https://github.com/lh3/seqtk), and chromosomes and scaffolds were numbered in order of size using SeqKit[70] (v0.7.2-dev). Chromosomes were later renamed and reoriented based on alignment with MUMmer[29] (v3.23) to the released assembly[63] (GCA_004786255.1 [https://www.ncbi.nlm.nih.gov/datasets/genome/GCA_004786255.1]).

Protein-coding gene annotation

Using the previously described final repeat library (n = 10,270; **Supplementary Note 2**), which combined the pan-anuran repeat library and ancestral RepBase dataset, the chromosome-scale assemblies of *E. coqui*, *E. pustulosus*, *H. boettgeri*, *L. ailaonicum*, and *P. adspersus* were soft masked with RepeatMasker[48,50] (v4.0.7 and v4.0.9). In addition to the RNA sequencing above, additional RNA data for *H. boettgeri*[7] (BioProject PRJNA306175 [https://www.ncbi.nlm.nih.gov/sra/?term=PRJNA306175]) and *P. adspersus*[63] (BioProject PRJNA439445 [https://www.ncbi.nlm.nih.gov/sra/?term=PRJNA439445]) were downloaded from the NCBI SRA. *E. coqui* RNA sequencing datasets of stages 7, 10, and 13 hindlimb (contact Mara Laslo at ml125@wellesley.edu) and stage 9–10 tail fin skin (BioProject PRJNA1022815 [https://www.ncbi.nlm.nih.gov/sra/?term=PRJNA1022815]) were obtained from Harvard University and the French National Center for Scientific Research, respectively. All RNA sequencing data were adapter trimmed with ea-utils[72] fastq-mcf (commit bd148d4) and aligned to the respective assemblies with STAR[53] (v2.5.3a and v2.7.0f), using the custom script STARalign.sh (v1.0, https://github.com/abmudd/Assembly).

Genome-guided transcriptomes were assembled with Trinity[56,57] (v2.5.1) for each individual RNA library: *E. coqui* (n = 7), *E. pustulosus* (n = 24), *H. boettgeri* (n = 9), and *P. adspersus* (n = 2). The assembled transcriptomes for these four species were aligned to the respective assemblies with STARlong (v2.7.1a) and then split into single-exon and multi-exon transcripts based on their alignments, using custom script filter_trinity.py (v1.0, https://github.com/abmudd/Assembly). Multi-exon transcripts were discarded if the first exon and/or last exon was less than 60 bp in length, if an intron was less than 60 bp or greater than 300,000 bp in length, or if the total transcript alignment length was less than 250 bp. Single-exon transcripts larger than 80 amino acids and containing start and stop codons were extracted with TransDecoder[57] (v3.0.1).

Filtered single-exon and multi-exon transcripts were combined and used as mRNA evidence by the IGC v5.0 pipeline[16] to annotate the four aforementioned genome assemblies. Peptides from the *X. tropicalis* annotation (**Supplementary Note 2**), as well as SwissProt eukaryotes[80] (downloaded November 2018), were similarly used as protein homology evidence.

Supplementary Note 4: Comparative analysis.

Gene homology and orthology

From the resulting annotations, gene homology between *E. coqui*, *E. pustulosus*, *H. boettgeri*, *L. ailaonicum*[77,78], *P. adspersus*, *X. laevis*[7] (v9), and *X. tropicalis* was analyzed with OrthoVenn2[5] (https://orthovenn2.bioinfotoolkits.net) using an E-value of 1×10^{-5} and an inflation value of 1.5. One-to-one gene orthologs between *E. coqui*, *E. pustulosus*, *H. boettgeri*, *P. adspersus*, and *X. tropicalis* were extracted from the OrthoVenn2 output, after requiring the ortholog sets to be either present in a single copy or absent in *L. ailaonicum*[77,78] and the L and S subgenomes of *X. laevis*[7] (v9). Regarding the exceptions for *L. ailaonicum* and *X. laevis*, an initial analysis of the OrthoVenn2 output found that the *L. ailaonicum* annotation[77,78] was missing 37% (3,670) of the one-to-one gene orthologs found in the five main frog species, whereas the *X. laevis* annotation[7] (v9) has confounding factors associated with the allopolyploidization and resulting gene evolution. In constructing this set, we also excluded genes on the *P. adspersus* W chromosome.

Whole-genome multiple alignment

The assemblies for *Ambystoma mexicanum*[81,82] (GCA_002915635.2 [https://www.ncbi.nlm.nih.gov/datasets/genome/GCA_002915635.2]), *E. coqui*, *E. pustulosus*, *H. boettgeri*, *L. ailaonicum*, and *P. adspersus* were each aligned pairwise against *X. tropicalis* with Cactus[83] (commit e4d0859). *X. laevis*[7] (v9) was broken into subgenomes, and the chromosomes of each subgenome were aligned against *X. tropicalis* with Cactus (commit e4d0859). As previously described[58], all pairwise output HAL alignment files were filtered and converted into MAF, using cactus_filter.py[58] (v1.0, https://github.com/abmudd/Assembly), and runs of collinearity were extracted from each pairwise MAF file. The pairwise MAF files were also merged with ROAST/MULTIZ[84] (v012109), using the phylogenetic topology from TimeTree[19] (http://www.timetree.org), and sorted with last[85] (v979).

Phylogeny

Using the 9,624 identified one-to-one orthologous genes and the ROAST-merged MAF file, as previously described[58], four-fold degenerate bases were extracted with script 4Dextract.py[58] (v1.0, https://github.com/abmudd/Assembly) and converted into PHYLIP format with BeforePhylo (commit 0885849, https://github.com/qiyunzhu/BeforePhylo). The maximum likelihood tree was estimated with RAxML[17] (v8.2.11) using the GTR+Gamma model of substitution with outgroup *A. mexicanum*.

Estimated divergence times

We estimated divergence times from the four-fold synonymous site alignment with MEGA7[20] (v7.0.26), as previously described[86]. The MEGA7 time tree was constructed using the Reltime method[87] with the GTR+Gamma model of substitution. The confidence intervals provided by TimeTree (http://www.timetree.org, retrieved on October 31, 2019) for all nodes except the *X. laevis* L – *X. laevis* S node were used as input to MEGA7. These input ranges and output times

are noted in **Supplementary Table 11**. The time calculated for the split between L and S subgenomes in *X. laevis* was substantiated in the literature[7,88].

Chromosome evolution

As previously described[58], pairwise alignments were extracted from the ROAST-merged MAF file using custom script extract2speciesmaf.py[58] (v1.0, https://github.com/abmudd/Assembly) and converted into runs of collinearity following the process used in cactus_filter.py[58] (v1.0, https://github.com/abmudd/Assembly). The runs of collinearity were visualized (**Supplementary Fig. 7**) with Circos[89] (v0.69-6) and, following file conversion with custom scripts mcscan_convert_links.py[58] (v1.0, https://github.com/abmudd/Assembly) and mcscan_invert_chr.py[58] (v1.0, https://github.com/abmudd/Assembly), with jcvi.graphics.karyotype (v0.8.12, https://github.com/tanghaibao/jcvi). The previously extracted one-to-one gene orthologs were similarly visualized with Circos[89] (v0.69-6) and jcvi.graphics.karyotype (v0.8.12, https://github.com/tanghaibao/jcvi). Based on these visualizations and the analyzed phylogeny, with the assumption of the parsimony principle, we extracted chromosome changes using the following logic: changes that were shared in the same order and orientation between two sister species were present in the common ancestor. Any changes that did not meet this criterion were classified as lineage-specific changes.

Propensity for centric translocation under a random break model

As shown in **Fig. 1**, only one of the 14 inferred translocation breakpoints split a chromosome arm—in *P. adspersus*, chromosomes 3 and 6 arise from a reciprocal translocation that breaks ancestral elements A and M. The remaining breaks are either Robertsonian (i.e., occur close to a centromere) or end-to-end (near a telomere). We asked whether the apparent concentration of breaks near centromeres and telomeres was consistent with a random breakage model.

The average chromosome size across our genomes (counting the L and S subgenomes of *X. laevis* separately) is 170 Mb (average genome size 1.88 Gb; n ~ 11 chromosomes/genome). To keep our model simple we generously assume that a break would be considered “arm preserving” if it occurred in a 10 Mb window centered on a centromere, or within 5 Mb of the end of a chromosome. Thus each chromosome contributes ~20 Mb of arm-preserving targets and 150 Mb of target space that would result in a non-Robertsonian or end-end event. Thus, under a random breakage model, the probability that an individual break is arm-preserving is 150 / 170 = 0.88. If the 14 breaks occurred at random along the genome, we would expect 14 × 0.88 = 12.3 chromosome arms to be broken, yet we observe only 2 (from the one reciprocal translocation in ancestral elements A and M that result in *P. adspersus* chromosomes 3 and 6). Under a simple Poisson random break model, the probability of 2 or fewer breaks is $(1 + 12.3 + 12.3^2 / 2) \times e^{-12.3} = 4\times10^{-4}$. We, therefore, reject a simple random break model.

Supplementary Note 5: Genomic landscape and 3D structure analysis.

Genomic landscape

Recombination rate and genomic landscapes

The genetic map from Mitros et al.[4] using GBS sequencing data (PRJNA526297 [https://www.ncbi.nlm.nih.gov/sra/?term=PRJNA526297]) was re-made on the final version of the genome (**Methods**). Of the total of 1,277 genetic markers, we considered 1,168 (91.5%) markers whose genetic distance was equal to or less than the distance of the marker immediately adjacent to the right. Recombination rates were calculated using the first derivative of the interpolated genetic distances (**Supplementary Fig. 10**). We observed that the recombination rate correlated the highest with satellite repeats, GC content, and the first principal component obtained from the repeat density matrix (**Supplementary Table 14**). We considered the distribution of recombination rates in the p- and q-arm subtelomeres (defined operationally as the 30 Mb at the ends of each chromosome, excluding 5 Mb around the centromere), and the remaining portion of each chromosome. Since the difference between p- and q-subtelomere recombination rate distributions were only slightly different (two-sample Kolmogorov–Smirnov, two-sided, Hochberg-corrected $p = 6.4\times10^{-3}$), we combined them. Comparing the combined subtelomeric distribution of recombination rate with the chromosome arms (excluding the pericentromere), however, we find a strongly significant difference by the two-sample Kolmogorov-Smirnov test (two-sided, Hochberg-corrected $p = 5.2\times10^{-321}$) (**Supplementary Fig. 10c**).

Notably, the 2p chromosome arm was absent in a previous map produced by Wells et al.[90] relative to the early and highly fragmented *X. tropicalis* genome assembly. While Wells et al. noted that they found scaffolds in the early draft genome that they could cytogenetically map to 2p (reported in Macha et al.[91]), nearly three-quarters of genotyped SSLP markers on these scaffolds were non-polymorphic in their F_2 mapping panel. Wells et al. compared these scaffolds to size-mapped controls and concluded that (in their mapping population) “the level of polymorphism on the p arm of chromosome 2 is substantially reduced relative to the regions represented on the map.” Such a situation could occur if the two parents of the F_2 population used by Wells *et al.* were cryptically related and shared haplotypes on chromosome 2p. Thus, the presence/absence of the 2p arm in our map/Wells *et al.* may simply be due to the difference between the founders of our respective mapping populations. Previously, we[4] inferred the existence of a recessive lethal allele in the ICB population on chromosome 2 that could lead to distorted segregation, and it is possible that these phenomena are related.

Identifying extended subtelomere boundaries with Hi-C read data

Inter-chromosomal Hi-C contact matrices were extracted at 1 Mb resolution at minimum mapping quality (MQ) ≥ 0 and MQ ≥ 30 thresholds for each chromosome 1-by-chromosome i pair with Juicer Tools (commit d3ee11b). To isolate repeat-specific signals observed in the

presumptive subtelomeres (**Fig. 4** and **Supplementary Fig. 1e**), MQ ≥ 30 observed counts were subtracted from MQ ≥ 0 observed counts. We used two methods to extract subtelomere region boundaries from the subtracted matrices: (1) a k-means clustering method implementing nine tessellated rectangular Voronoi cells (**Supplementary Fig. 2b**) categorized into three classes (corners, center, and edges) constrained to change only their relative dimensions during the optimization procedure, and (2) a principal component analysis (PCA) of the same subtracted matrix (**Supplementary Fig. 2c**). Computations were performed in R v3.5.0 with the "prcomp" function. See (hic-analysis.R, https://bitbucket.org/rokhsar-lab/xentr10/src/master/hic) for implementation details.

Classification of pericentromeric and subtelomeric regions based on repeat content

To determine the sources of variation in repeat content across the genome we used the repeat density matrix as input for the Principal Component Analysis (PCA) (**Methods**). The first three principal components described 7.53% of the total variance and discriminate landmarks of repetitive regions. The first two principal components describe the higher-order structure of the chromosomes (**Supplementary Fig. 5a**). The first principal component (PC1) is correlated with GC% content (Pearson's r = +0.796) and recombination rate (Pearson's r = +0.77, **Supplementary Fig. 5a**). The second principal component (PC2) describes the repeats present in the pericentromeric and distal subtelomeric regions. The peaks observed in PC2 at the interior of the chromosomes are in close proximity to the Hi-C-estimated centromere positions and surrounding pericentromeric repeats. To estimate the centromere positions using repeat content, we smoothed the PC2 vector of each chromosome using the natural cubic spline method (*knots = 40*). The position of the peak summit, the highest and widest PC2-smoothed values, was considered the centromere estimate of each chromosome (**Supplementary Fig. 5a**). The third principal component (PC3) captures the variance of repeats differentially associated with A/B compartments (**Supplementary Fig. 5b**). The alternating signs in the Hi-C PC1 vector loadings coincide (inversely) with the shift in signs from repeat density PC3 vector, showing a moderate (negative) correlation between repeat composition and A/B compartments (Pearson's r = −0.44). Harbinger-N9_XT is positively associated with PC3 (Pearson's r = +0.50), whereas DNA/hAT-Ac appears strongly negatively correlated with PC3 (Pearson's r = −0.80).

Identification of tandem repeats enriched in centromeric and subtelomeric regions

Centromeric tandem repeats were identified as described in **Methods**. Pericentromeric tandem repeats shown in **Supplementary Fig. 11a** are enriched 4-fold in the pericentromeric region compared to the rest of the chromosome and have a footprint > 20 kb inside the pericentromeric region. Subtelomere-enriched repeats were enriched at least 8-fold in subtelomeric regions and a footprint > 50 Kb in the subtelomeres of all (sub)metacentric chromosomes (**Supplementary Fig. 11a**, **Supplementary Table 15**). Monomer "a" represented in **Supplementary Fig. 11a** corresponds to the 205-bp consensus monomer (below) that forms part of the centromeric tandem repeat in all chromosomes in *Xenopus tropicalis* (**Supplementary Fig. 9d**). The multiple sequence alignment of the monomeric units span blocks between 41 and 258 kb per

chromosome (**Supplementary Table 12**) and exhibit an average sequence identity over 95% at the per-base level (**Supplementary Fig. 9e**).

Some tandem repeats (**Supplementary Fig. 11a**) have sequence similarity to consensus repetitive elements (**Supplementary Table 16**). The distribution of the repeats, their lengths, density, and the JC distances from the consensus sequence indicate that repeats near the subtelomeres are targets of tandem repeat expansion (**Supplementary Fig. 5**).

```
>Xt_CR Xenopus tropicalis centromeric tandem repeat
TTGAATGCTACATGGCATTTGAAAGACAGTGCAGAAAATAAGCTCCTGCTAACGTTTCATGCTTGCAAACCAATCAGAGCACTTG
CAGTAACATGGGCTAAAACGCTTTACAGAGCAAACCGGCAAACTGAAGCAAATCACGCGAATGCCTCAGAAAAGCTCATTTAACA
CACAAAATTGACTATCTATAAGACAAAGTAAATAG
```

Centromere inference with Hi-C

Examining the genome-wide Hi-C contact matrix revealed “angel wing”-shaped inter-chromosomal contact patterns and puncta derived from centromere clustering (**Fig. 1** and **Supplementary Fig. 1e**), similar to the Rabl-like patterns described for budding yeast by Duan et al.[92]. This motivated estimating the centromeric positions using Hi-C data (**Supplementary Fig. 9a**). Centurion[93] v0.1.0-3-g985439c was invoked using MQ ≥ 0 contact maps (for which automated estimates were more stable; **Supplementary Fig. 9b**) at 1 Mb matrix resolution, and with *coef* = 10, to refine initial centromere positions made by eye in Juicebox. All ten estimates localized between the centromere-flanking genes described by Uno et al.[94].

The above procedure was then similarly conducted for *E. coqui*, *E. pustulosus*, *P. adspersus*, *H. boettgeri*, and *X. laevis*. Some of these species, however, have telocentric and/or acrocentric chromosomes and Centurion could not reliably estimate the centromere positions for those chromosomes. The centromere positions for these chromosomes, and those with Centurion-estimated (C_c) positions differing from their initial estimates (C_i) by more than 10% (calculated by equation (1)) were reverted to their initial by-eye estimate.

$$(1)\ C_\Delta = 100 \cdot (C_c - C_i)/\max(C_i, L - C_i)$$

Where L is chromosome length. Such cases include *E. coqui* chromosomes 2, 3, 6, 7, and 9–13; *E. pustulosus* chromosomes 8–11; *H. boettgeri* chromosomes 4, 6, 7, and 9; and *P. adspersus* chromosomes 9 and W.

Cenp-a binding to CTR-A tandem repeats

ChIP-seq targeting Cenp-a and Histones H3 and H4 was performed as described in **Methods**. Over 97% of all Cenp-a ChIP-seq reads were mapped to the genome (**Supplementary Table 13**). With H3 and H4 ChIP-seq there is an apparent enrichment of reads at the pericentromeric regions, suggesting a ~2–5× assembly collapse in the length of the centromeric repeats. The collapse is likely caused by the lack of variants that would enable the assembly of our PacBio

reads across the long centromeric sequence. The ratios between Cenp-a/H4 and Cenp-a/S2 are shown in **Supplementary Fig. 9c** and **Supplementary Table 13**.

Three-dimensional chromosome structure

A/B compartment structure inference using Hi-C

Hi-C reads from *X. tropicalis* blood cells were aligned to the v10 assembly with Juicer[30,31] (commit d3ee11b). Normalized (observed over expected, Knight-Ruiz balanced) intra-chromosomal Hi-C contact matrices were then extracted at 250 kb matrix resolution with Juicer Tools (commit d3ee11b) from read pairs with MQ ≥ 30. An R[95] (v3.5.0) script implementing a sliding-window-based principal component analysis (PCA) algorithm was used to call compartment structure along each chromosome (call-compartments.R v0.1.0, https://bitbucket.org/bredeson/artisanal). Localizing the PCA along the diagonal of the Pearson correlation matrix with sliding windows (width 80 with a step of 40) mitigates the confounding signal introduced by intra-chromosomal p-q arm contacts, thereby amplifying the underlying A/B compartment signal. **Fig. 5a**,**b** presents the correlation matrix describing the compartment structure for chromosome 1 and the corresponding principal component (PC) vector obtained from the localized, sliding window PCA algorithm.

Gene densities (gene count/Mb) were calculated for each compartment bin with BEDtools intersect[96] (v2.28.0). The A and B compartment assignments were determined for each chromosome by obtaining the correlation between gene density (genes count/Mb) and the eigenvector of the Hi-C correlation matrix. If a chromosome exhibited a negative correlation between gene density and the Hi-C eigenvector, then the signs of the eigenvector for that chromosome was inverted (i.e., multiplied by −1.0). Compartment A was assigned to bins associated with high gene density. Repeat densities were subsequently calculated for A and B compartments. The above methods were performed for all species included in this study.

Rabl-like HiC patterns observed in other species

Hoencamp et al.[21] surveyed 24 plant and animal species using Hi-C and observed Rabl-like patterns in 14 (58.3%) of them: *Xenopus laevis*, *Ciona robusta*, (formerly *C. intestinalis*), *Aedes aegypti*, *Culex quinquefasciatus*, *Drosophila melanogaster*, *Hypsibius dujardini*, *Clonorchis sinensis*, *Cristatella mucedo*, *Acropora millepora*, *Pleurobrachia bachei*, *Agaricus bisporus*, *Saccharomyces cerevisiae*, *Arachis hypogaea*, and *Triticum aestivum* (see https://tiny.3dg.io/hdeb2021s2). Out of 7 vertebrates sampled in this study, however, only *Xenopus laevis* fibroblasts showed a Rabl-like pattern. Rabl-like Hi-C patterns have also been observed in single-cell organisms, such as baker's yeast[93] (as noted above) and apicomplexan parasites[97]. Such a broad sampling of species with Rabl-like Hi-C patterns suggests that this chromosome configuration may be a primitive feature of eukaryotic chromosome biology.

In addition to Hoencamp et al., numerous mammalian genome analyses conspicuously lack such Rabl-like patterns, including muntjak[58] fibroblasts; eight human cell types[98]; Rhesus macaque[99] female fibroblasts; mouse CH12-LX B-Lymphoblasts[98], Patski cell lines[99], and

activated B cells[100]; California sea lion (*Zalophus californianus*) whole blood[101,102]; Cheetah (*Acinonyx jubatus*) blood[102,103]; Collared lemur (*Eulemur collaris*) liver tissue[102]; Red panda (*Ailurus fulgens*) blood[102,104]; Virginia opossum (*Didelphis virginiana*) blood[102]; and Red kangaroo (*Macropus rufus*) blood[102]. The presence of Rabl-like Hi-C patterns, however, evidently depends on cell type and cell cycle stage with Rabl-like patterns observed in mouse hematopoietic cells[105], haploid G1-phase ES cells[106], diploid embryonic stem cells[106], and peripheral blood mononuclear cells[106].

Dissecting Rabl-like chromosome structure in *Xenopus tropicalis*

Because A/B compartmentalization (i.e., the checkerboard pattern) and high-density contacts between adjacent genomic loci (i.e., the strong diagonal band) were dominant sources of variance within chromosomes, dense inter-chromosomal contact matrices were used as a proxy for analyzing the Rabl-like structure of chromosomes within the nucleus. We extracted the observed counts of MQ ≥ 30 contacts at 1 Mb resolution for each chromosome 1-by-chromosome i pair. All calculations were performed using a custom R script (hic-analysis.R, https://bitbucket.org/rokhsar-lab/xentr10/src/master/hic).

ACA scores[21] were calculated using the four largest (sub)metacentric autosomal chromosomes from each species with MQ ≥ 60 contacts. The degree of polarized chromosome organization (**Supplementary Fig. 15**, **Supplementary Table 18**) was quantified by estimating the relative enrichment of two classes of Rabl-like contacts extracted from the above matrices: (1) centromere-centromere contact densities, collected within a window centered on the centromere with a radius 10% of the chromosome arm's length, and (2) inter-chromosome inter-arm contacts along centromere-to-telomere axes, within bands 10% the width of the chromosome's arm length. The median was calculated from the extracted class of contact densities, then divided by the median contact densities collected (within a 25% chromosome arm-length radius) from centromere-telomere intersected edges of the matrix. The median relative enrichment was used because it proved more robust to extreme-valued outliers).

For PCA-based visualization, raw matrix counts were smoothed using a radius 5% of each chromosome's length and taking the median of the sampled values (the median provided more stable smoothing in the presence of extreme-valued outliers, and so was preferred over the arithmetic mean). We performed PCA with the "prcomp" function in R[95] (v3.5.0) on the smoothed matrix and plotted PC1 vs. PC2 (**Supplementary Fig. 16**). We measured the strength of each chromosome's Rabl-like structure as the sum of squared distances (SSD) between chromosome arms (**Supplementary Table 19**) with the expectation that perfectly constrained chromosome arms in a Rabl configuration will result in an SSD approaching zero, and unconstrained (i.e., freely-moving) arms taking large values. SSD was computed as the squared Euclidean distances, in PC1-PC2 dimensions, between points $c - i$ and $c + i$, for all $i = 1 \rightarrow n$, where c is the index of the 1-Mb window containing the centromere and n is the number of non-overlapping windows tiled along the shortest chromosome arm. The significance p-value for each chromosome's observed Rabl-like structure was calculated by permutation testing (10,000 iterations, one-sided α = 0.01), with variance as the test statistic. Each permutation started from a smoothed matrix, itself derived from a raw-count matrix reordered by 5,000 random row-

/column-swap operations, the PCA computed, and the variance of radian angle change (i.e., second derivative) calculated between two vectors created by triplets of consecutively-ordered points along a chromosome. Mathematically, this is calculated using equations (2–6):

(2) $\sigma^2 = \mathrm{var}_{i=1\to n-2}\big(\theta(\boldsymbol{v}_{i+1},\boldsymbol{u}) - \theta(\boldsymbol{v}_i,\boldsymbol{u})\big),$

where

(3) $\theta(\boldsymbol{v},\boldsymbol{u}) = \arccos\left((\boldsymbol{v}\cdot\boldsymbol{u})/(|\boldsymbol{v}|\cdot|\boldsymbol{u}|)\right)$

(4) $\boldsymbol{v}_{i+1} = (\mathrm{PC1}_{i+2} - \mathrm{PC1}_{i+1}, \mathrm{PC2}_{i+2} - \mathrm{PC2}_{i+1})$

(5) $\boldsymbol{v}_i = (\mathrm{PC1}_{i+1} - \mathrm{PC1}_i, \mathrm{PC2}_{i+1} - \mathrm{PC2}_i)$

(6) $\boldsymbol{u} = (\mathrm{sign}\,(\mathrm{PC1}_{i+1} - \mathrm{PC1}_i), 0)$

Two vectors connecting three consecutively ordered points along a linear path introduce relatively little angular change (i.e., little variance), whereas unpatterned (randomly ordered) points tend to induce random directional changes, and greater angular change (i.e., greater variance), between connected vectors. Family-wise p-value correction was performed using the Hochberg[107] step-up procedure with the "p.adjust" function in R[95] (v3.5.0).

Supplementary References

1. Durand, N. C. *et al.* Juicebox provides a visualization system for Hi-C contact maps with unlimited zoom. *Cell Systems* **3**, 99–101 (2016).
2. Dudchenko, O., Shamim, M. S., Batra, S. S. & Durand, N. C. The Juicebox Assembly Tools module facilitates de novo assembly of mammalian genomes with chromosome-length scaffolds for under $1000. *Biorxiv* (2018).
3. Robinson, J. T. *et al.* Integrative genomics viewer. *Nat. Biotechnol.* **29**, 24–26 (2011).
4. Mitros, T. *et al.* A chromosome-scale genome assembly and dense genetic map for *Xenopus tropicalis*. *Dev. Biol.* **452**, 8–20 (2019).
5. Xu, L. *et al.* OrthoVenn2: A web server for whole-genome comparison and annotation of orthologous clusters across multiple species. *Nucleic Acids Research* **47**, W52–W58 (2019).
6. Kent, W. J., Baertsch, R., Hinrichs, A., Miller, W. & Haussler, D. Evolution's cauldron: duplication, deletion, and rearrangement in the mouse and human genomes. *Proc. Natl. Acad. Sci. U. S. A.* **100**, 11484–11489 (2003).
7. Session, A. M. *et al.* Genome evolution in the allotetraploid frog *Xenopus laevis*. *Nature* **538**, 336–343 (2016).
8. Myers, S., Bottolo, L., Freeman, C., McVean, G. & Donnelly, P. A fine-scale map of recombination rates and hotspots across the human genome. *Science* **310**, 321–324 (2005).
9. Shifman, S. *et al.* A high-resolution single nucleotide polymorphism genetic map of the mouse genome. *PLoS Biol.* **4**, e395 (2006).
10. Backstrom, N. *et al.* The recombination landscape of the zebra finch *Taeniopygia guttata* genome. *Genome Research* **20**, 485–495 (2010).
11. Ogiwara, I. V-SINEs: A new superfamily of vertebrate SINEs that are widespread in vertebrate genomes and retain a strongly conserved segment within each repetitive unit. *Genome Research* **12**, 316–324 (2002).
12. Tymowska, J. Karyotype analysis of *Xenopus tropicalis* Gray, Pipidae. *Cytogenetic and Genome Research* **12**, 297–304 (1973).
13. Simão, F. A., Waterhouse, R. M., Ioannidis, P., Kriventseva, E. V. & Zdobnov, E. M. BUSCO: Assessing genome assembly and annotation completeness with single-copy orthologs. *Bioinformatics* **31**, 3210–3212 (2015).
14. Waterhouse, R. M. *et al.* BUSCO applications from quality assessments to gene prediction and phylogenomics. *Mol. Biol. Evol.* **35**, 543–548 (2018).
15. Zdobnov, E. M. *et al.* OrthoDB v9.1: cataloging evolutionary and functional annotations for animal, fungal, plant, archaeal, bacterial and viral orthologs. *Nucleic Acids Res.* **45**, D744–D749 (2017).
16. Shu, S., Rokhsar, D., Goodstein, D., Hayes, D. & Mitros, T. *JGI Plant Genomics Gene Annotation Pipeline*. https://www.osti.gov/biblio/1241222 (2014).
17. Stamatakis, A. RAxML version 8: a tool for phylogenetic analysis and post-analysis of large phylogenies. *Bioinformatics* **30**, 1312–1313 (2014).
18. Junier, T. & Zdobnov, E. M. The Newick utilities: high-throughput phylogenetic tree processing in the UNIX shell. *Bioinformatics* **26**, 1669–1670 (2010).

19. Kumar, S., Stecher, G., Suleski, M. & Hedges, S. B. TimeTree: A resource for timelines, timetrees, and divergence times. *Mol. Biol. Evol.* **34**, 1812–1819 (2017).
20. Kumar, S., Stecher, G. & Tamura, K. MEGA7: Molecular Evolutionary Genetics Analysis version 7.0 for bigger datasets. *Mol. Biol. Evol.* **33**, 1870–1874 (2016).
21. Hoencamp, C. *et al.* 3D genomics across the tree of life reveals condensin II as a determinant of architecture type. *Science* **372**, 984–989 (2021).
22. Niu, L. *et al.* Three-dimensional folding dynamics of the *Xenopus tropicalis* genome. *Nat. Genet.* **53**, 1075–1087 (2021).
23. Koren, S. *et al.* Canu: Scalable and accurate long-read assembly via adaptive k-mer weighting and repeat separation. *Genome Res.* **27**, 722–736 (2017).
24. Ye, C., Hill, C. M., Wu, S., Ruan, J. & Ma, Z. S. DBG2OLC: Efficient assembly of large genomes using long erroneous reads of the third generation sequencing technologies. *Sci. Rep.* **6**, 31900 (2016).
25. Chin, C.-S. *et al.* Nonhybrid, finished microbial genome assemblies from long-read SMRT sequencing data. *Nat. Methods* **10**, 563–569 (2013).
26. Chaisson, M. J. & Tesler, G. Mapping single molecule sequencing reads using basic local alignment with successive refinement (BLASR): Application and theory. *BMC Bioinformatics* **13**, 238 (2012).
27. Weisenfeld, N. I., Kumar, V., Shah, P., Church, D. M. & Jaffe, D. B. Direct determination of diploid genome sequences. *Genome Res.* **27**, 757–767 (2017).
28. Chakraborty, M., Baldwin-Brown, J. G., Long, A. D. & Emerson, J. J. Contiguous and accurate de novo assembly of metazoan genomes with modest long read coverage. *Nucleic Acids Research* **44**, e147–e147 (2016).
29. Kurtz, S. *et al.* Versatile and open software for comparing large genomes. *Genome Biol.* **5**, R12 (2004).
30. Durand, N. C. *et al.* Juicer provides a one-click system for analyzing loop-resolution Hi-C experiments. *Cell Syst* **3**, 95–98 (2016).
31. Tange, O. *GNU Parallel 2018*. (Lulu.com, 2018). doi:10.5281/zenodo.1146014.
32. Dudchenko, O. *et al.* De novo assembly of the *Aedes aegypti* genome using Hi-C yields chromosome-length scaffolds. *Science* **356**, 92–95 (2017).
33. Wu, T. D. & Watanabe, C. K. GMAP: a genomic mapping and alignment program for mRNA and EST sequences. *Bioinformatics* **21**, 1859–1875 (2005).
34. Li, H. Minimap2: Pairwise alignment for nucleotide sequences. *Bioinformatics* **34**, 3094–3100 (2018).
35. Boetzer, M., Henkel, C. V., Jansen, H. J., Butler, D. & Pirovano, W. Scaffolding pre-assembled contigs using SSPACE. *Bioinformatics* **27**, 578–579 (2011).
36. Li, H. Aligning sequence reads, clone sequences and assembly contigs with BWA-MEM. *arXiv [q-bio.GN]* (2013).
37. English, A. C. *et al.* Mind the gap: upgrading genomes with Pacific Biosciences RS long-read sequencing technology. *PLoS One* **7**, e47768 (2012).
38. Walker, B. J. *et al.* Pilon: An integrated tool for comprehensive microbial variant detection and genome assembly improvement. *PLoS ONE* **9**, e112963 (2014).
39. Li, H. *et al.* The Sequence Alignment/Map format and SAMtools. *Bioinformatics* **25**, 2078–2079 (2009).

40. Garrison, E. & Marth, G. Haplotype-based variant detection from short-read sequencing. *arXiv [q-bio.GN]* (2012).
41. Temple, G. *et al.* The completion of the mammalian gene collection (MGC). *Genome Res.* **19**, 2324–2333 (2009).
42. Camacho, C. *et al.* BLAST+: Architecture and applications. *BMC Bioinformatics* **10**, 421 (2009).
43. Marçais, G. & Kingsford, C. A fast, lock-free approach for efficient parallel counting of occurrences of k-mers. *Bioinformatics* **27**, 764–770 (2011).
44. Ranallo-Benavidez, T. R., Jaron, K. S. & Schatz, M. C. GenomeScope 2.0 and Smudgeplot for reference-free profiling of polyploid genomes. *Nat. Commun.* **11**, 1432 (2020).
45. Hellsten, U. *et al.* The genome of the Western clawed frog *Xenopus tropicalis*. *Science* **328**, 633–636 (2010).
46. Bredeson, J. V. *et al.* Sequencing wild and cultivated cassava and related species reveals extensive interspecific hybridization and genetic diversity. *Nat. Biotechnol.* **34**, 562–570 (2016).
47. Igawa, T. *et al.* Inbreeding ratio and genetic relationships among strains of the Western clawed frog, *Xenopus tropicalis*. *PLoS One* **10**, e0133963 (2015).
48. Smit, A. F. A. & Hubley, R. *RepeatModeler Open-1.0*. (2008–2015).
49. Jurka, J. *et al.* Repbase Update, a database of eukaryotic repetitive elements. *Cytogenet. Genome Res.* **110**, 462–467 (2005).
50. Smit, A. F. A., Hubley, R. & Green, P. *RepeatMasker Open-4.0*. (2013–2015).
51. Benson, G. Tandem Repeats Finder: A program to analyze DNA sequences. *Nucleic Acids Research* **27**, 573–580 (1999).
52. Zhang, J., Kobert, K., Flouri, T. & Stamatakis, A. PEAR: a fast and accurate Illumina Paired-End reAd mergeR. *Bioinformatics* **30**, 614–620 (2014).
53. Dobin, A. *et al.* STAR: ultrafast universal RNA-seq aligner. *Bioinformatics* **29**, 15–21 (2013).
54. Marin, R. *et al.* Convergent origination of a *Drosophila*-like dosage compensation mechanism in a reptile lineage. *Genome Research* **27**, 1974–1987 (2017).
55. Owens, N. D. L. *et al.* Measuring absolute RNA copy numbers at high temporal resolution reveals transcriptome kinetics in development. *Cell Reports* **14**, 632–647 (2016).
56. Grabherr, M. G. *et al.* Full-length transcriptome assembly from RNA-Seq data without a reference genome. *Nat. Biotechnol.* **29**, 644–652 (2011).
57. Haas, B. J. *et al.* De novo transcript sequence reconstruction from RNA-seq using the Trinity platform for reference generation and analysis. *Nat. Protoc.* **8**, 1494–1512 (2013).
58. Mudd, A. B., Bredeson, J. V., Baum, R., Hockemeyer, D. & Rokhsar, D. S. Analysis of muntjac deer genome and chromatin architecture reveals rapid karyotype evolution. *Communications Biology* **3**, 1–10 (2020).
59. Zhang, P. *et al.* Efficient sequencing of anuran mtDNAs and a mitogenomic exploration of the phylogeny and evolution of frogs. *Molecular Biology and Evolution* **30**, 1899–1915 (2013).
60. Brelsford, A., Dufresnes, C. & Perrin, N. High-density sex-specific linkage maps of a European tree frog (*Hyla arborea*) identify the sex chromosome without information on

offspring sex. *Heredity* **116**, 177–181 (2016).
61. Sun, Y.-B. *et al.* Whole-genome sequence of the Tibetan frog *Nanorana parkeri* and the comparative evolution of tetrapod genomes. *Proc. Natl. Acad. Sci. U. S. A.* **112**, E1257–62 (2015).
62. Liu, S. *et al.* Genomic data from the Tibetan Plateau frog (*Nanorana parkeri*). (2015) doi:10.5524/100132.
63. Denton, R. D., Kudra, R. S., Malcom, J. W., Du Preez, L. & Malone, J. H. The African Bullfrog (*Pyxicephalus adspersus*) genome unites the two ancestral ingredients for making vertebrate sex chromosomes. *Cold Spring Harbor Laboratory* 329847 (2018) doi:10.1101/329847.
64. Hammond, S. A. *et al.* The North American bullfrog draft genome provides insight into hormonal regulation of long noncoding RNA. *Nat. Commun.* **8**, 1433 (2017).
65. Palomar, G. *et al.* Comparative high-density linkage mapping reveals conserved genome structure but variation in levels of heterochiasmy and location of recombination cold spots in the common frog. *G3* **7**, 637–645 (2017).
66. Palomar, G. *et al.* Draft genome *Rana temporaria*. (2016) doi:10.6084/m9.figshare.4483439.v1.
67. International Cassava Genetic Map Consortium (ICGMC). High-resolution linkage map and chromosome-scale genome assembly for cassava (*Manihot esculenta* Crantz) from 10 populations. *G3* **5**, 133–144 (2015).
68. Putnam, N. H. *et al.* Chromosome-scale shotgun assembly using an in vitro method for long-range linkage. *Genome Res.* **26**, 342–350 (2016).
69. Kajitani, R. *et al.* Efficient de novo assembly of highly heterozygous genomes from whole-genome shotgun short reads. *Genome Res.* **24**, 1384–1395 (2014).
70. Shen, W., Le, S., Li, Y. & Hu, F. SeqKit: A cross-platform and ultrafast toolkit for FASTA/Q file manipulation. *PLoS One* **11**, e0163962 (2016).
71. Irisarri, I., Vences, M., Mauro, D. S., Glaw, F. & Zardoya, R. Reversal to air-driven sound production revealed by a molecular phylogeny of tongueless frogs, family Pipidae. *BMC Evolutionary Biology* **11**, 1–10 (2011).
72. Aronesty, E. Comparison of sequencing utility programs. *TOBIOIJ* **7**, 1–8 (2013).
73. O'Connell, J. *et al.* NxTrim: Optimized trimming of Illumina mate pair reads. *Bioinformatics* **31**, 2035–2037 (2015).
74. Chapman, J. A. *et al.* Meraculous: De novo genome assembly with short paired-end reads. *PLoS One* **6**, e23501 (2011).
75. Goltsman, E., Ho, I. & Rokhsar, D. Meraculous-2D: Haplotype-sensitive assembly of highly heterozygous genomes. *arXiv [q-bio.GN]* (2017).
76. Dierckxsens, N., Mardulyn, P. & Smits, G. NOVOPlasty: de novo assembly of organelle genomes from whole genome data. *Nucleic Acids Res.* **45**, e18 (2017).
77. Li, Y. *et al.* Chromosome-level assembly of the mustache toad genome using third-generation DNA sequencing and Hi-C analysis. *Gigascience* **8**, (2019).
78. Dingqi, R. *et al.* Supporting data for 'Chromosomal-level assembly of the mustache toad genome using third-generation DNA sequencing and Hi-C analysis'. (2019) doi:10.5524/100624.
79. Xu, Q., Liu, S., Wan, R., Yue, B. & Zhang, X. The complete mitochondrial genome of the

Vibrissaphora boringii (Anura: Megophryidae). *Mitochondrial DNA A DNA Mapp Seq Anal* **27**, 758–759 (2016).
80. UniProt Consortium. UniProt: a worldwide hub of protein knowledge. *Nucleic Acids Res.* **47**, D506–D515 (2019).
81. Nowoshilow, S. *et al.* The axolotl genome and the evolution of key tissue formation regulators. *Nature* **554**, 50–55 (2018).
82. Smith, J. J. *et al.* A chromosome-scale assembly of the axolotl genome. *Genome Res.* **29**, 317–324 (2019).
83. Paten, B. *et al.* Cactus: Algorithms for genome multiple sequence alignment. *Genome Res.* **21**, 1512–1528 (2011).
84. Blanchette, M. *et al.* Aligning multiple genomic sequences with the threaded blockset aligner. *Genome Res.* **14**, 708–715 (2004).
85. Kiełbasa, S. M., Wan, R., Sato, K., Horton, P. & Frith, M. C. Adaptive seeds tame genomic sequence comparison. *Genome Res.* **21**, 487–493 (2011).
86. Mello, B. Estimating timetrees with MEGA and the TimeTree resource. *Mol. Biol. Evol.* **35**, 2334–2342 (2018).
87. Tamura, K. *et al.* Estimating divergence times in large molecular phylogenies. *Proc. Natl. Acad. Sci. U. S. A.* **109**, 19333–19338 (2012).
88. Evans, B. J., Kelley, D. B., Tinsley, R. C., Melnick, D. J. & Cannatella, D. C. A mitochondrial DNA phylogeny of African clawed frogs: phylogeography and implications for polyploid evolution. *Mol. Phylogenet. Evol.* **33**, 197–213 (2004).
89. Krzywinski, M. *et al.* Circos: an information aesthetic for comparative genomics. *Genome Res.* **19**, 1639–1645 (2009).
90. Wells, D. E. *et al.* A genetic map of *Xenopus tropicalis*. *Dev. Biol.* **354**, 1–8 (2011).
91. Mácha, J. *et al.* Deep ancestry of mammalian X chromosome revealed by comparison with the basal tetrapod *Xenopus tropicalis*. *BMC Genomics* **13**, 315 (2012).
92. Duan, Z. *et al.* A three-dimensional model of the yeast genome. *Nature* **465**, 363–367 (2010).
93. Varoquaux, N. *et al.* Accurate identification of centromere locations in yeast genomes using Hi-C. *Nucleic Acids Res.* **43**, 5331–5339 (2015).
94. Uno, Y., Nishida, C., Takagi, C., Ueno, N. & Matsuda, Y. Homoeologous chromosomes of *Xenopus laevis* are highly conserved after whole-genome duplication. *Heredity* vol. 111 430–436 Preprint at https://doi.org/10.1038/hdy.2013.65 (2013).
95. R Core Team. R Core Team. R: A language and environment for statistical computing. *Foundation for Statistical Computing* (2013).
96. Quinlan, A. R. BEDTools: The Swiss-army tool for genome feature analysis. *Curr. Protoc. Bioinformatics* **47**, 11.12.1–34 (2014).
97. Bunnik, E. M. *et al.* Comparative 3D genome organization in apicomplexan parasites. *Proc. Natl. Acad. Sci. U. S. A.* **116**, 3183–3192 (2019).
98. Rao, S. S. P. *et al.* A 3D map of the human genome at kilobase resolution reveals principles of chromatin looping. *Cell* **159**, 1665–1680 (2014).
99. Darrow, E. M. *et al.* Deletion of DXZ4 on the human inactive X chromosome alters higher-order genome architecture. *Proc. Natl. Acad. Sci. U. S. A.* **113**, E4504–12 (2016).
100. Vian, L. *et al.* The Energetics and Physiological Impact of Cohesin Extrusion. *Cell* **175**,

292–294 (2018).
101. Peart, C. R. *et al.* Hi-C scaffolded short- and long-read genome assemblies of the California sea lion are broadly consistent for syntenic inference across 45 million years of evolution. *Mol. Ecol. Resour.* **21**, 2455–2470 (2021).
102. DNA Zoo Assemblies. *DNA Zoo* https://www.dnazoo.org/assemblies (2018).
103. Dobrynin, P. *et al.* Genomic legacy of the African cheetah, *Acinonyx jubatus*. *Genome Biol.* **16**, 277 (2015).
104. Hu, Y. *et al.* Comparative genomics reveals convergent evolution between the bamboo-eating giant and red pandas. *Proc. Natl. Acad. Sci. U. S. A.* **114**, 1081–1086 (2017).
105. Zhang, C. *et al.* tagHi-C Reveals 3D Chromatin Architecture Dynamics during Mouse Hematopoiesis. *Cell Rep.* **32**, 108206 (2020).
106. Stevens, T. J. *et al.* 3D structures of individual mammalian genomes studied by single-cell Hi-C. *Nature* **544**, 59–64 (2017).
107. Hochberg, Y. A sharper Bonferroni procedure for multiple tests of significance. *Biometrika* **75**, 800–803 (1988).
