# Supplementary material for: Conserved chromatin and repetitive patterns reveal slow genome evolution in frogs
Source: Nat Commun. 2024 Jan 17;15:579. doi: 10.1038/s41467-023-43012-9 (PMC10794172; doi:10.1038/s41467-023-43012-9)
Supplement: Supplementary file 3 — Description of Additional Supplementary Files [file 41467_2023_43012_MOESM3_ESM.pdf]

Description of Additional Supplementary Files

File name: Supplementary Data 1
Description: Table of sequencing data. An MS Excel file summarizing the sequencing data used to construct the six frog genome assemblies new or updated in this study, as well as the RNA-seq data used for annotating their protein-coding genes. The *X. tropicalis* ChIP-seq data are also included.

File name: Supplementary Data 2
Description: Genetic markers. An MS Excel file containing the marker number, locus identifier, chromosome name, centiMorgan position, and nucleotide position for each genetic marker in the F_2 *X. tropicalis* genetic linkage map.
