# Supplementary material for: Conserved chromatin and repetitive patterns reveal slow genome evolution in frogs
Source: Nat Commun. 2024 Jan 17;15:579. doi: 10.1038/s41467-023-43012-9 (PMC10794172; doi:10.1038/s41467-023-43012-9)
Supplement: Supplementary file 6 — Reporting Summary [file 41467_2023_43012_MOESM6_ESM.pdf]

nature portfolio

Corresponding author(s): Daniel S. Rokhsar

Last updated by author(s): Oct 5, 2023

Reporting Summary

Nature Portfolio wishes to improve the reproducibility of the work that we publish. This form provides structure for consistency and transparency in reporting. For further information on Nature Portfolio policies, see our Editorial Policies and the Editorial Policy Checklist.

Statistics

For all statistical analyses, confirm that the following items are present in the figure legend, table legend, main text, or Methods section.

n/a	Confirmed	
☐	☒	The exact sample size (*n*) for each experimental group/condition, given as a discrete number and unit of measurement
☒	☐	A statement on whether measurements were taken from distinct samples or whether the same sample was measured repeatedly
☐	☒	The statistical test(s) used AND whether they are one- or two-sided<br>*Only common tests should be described solely by name; describe more complex techniques in the Methods section.*
☒	☐	A description of all covariates tested
☐	☒	A description of any assumptions or corrections, such as tests of normality and adjustment for multiple comparisons
☐	☒	A full description of the statistical parameters including central tendency (e.g. means) or other basic estimates (e.g. regression coefficient) AND variation (e.g. standard deviation) or associated estimates of uncertainty (e.g. confidence intervals)
☐	☒	For null hypothesis testing, the test statistic (e.g. *F*, *t*, *r*) with confidence intervals, effect sizes, degrees of freedom and *P* value noted<br>*Give P values as exact values whenever suitable.*
☒	☐	For Bayesian analysis, information on the choice of priors and Markov chain Monte Carlo settings
☒	☐	For hierarchical and complex designs, identification of the appropriate level for tests and full reporting of outcomes
☐	☒	Estimates of effect sizes (e.g. Cohen's *d*, Pearson's *r*), indicating how they were calculated

Our web collection on statistics for biologists contains articles on many of the points above.

Software and code

Policy information about availability of computer code

Data collection Metamorph (v7.0), TimeTree (online tool)

Data analysis The following software and their versions are declared in the manuscript Methods sections:

3D-DNA (commit 2796c3b)
Arrow (smrtlink v6.0.0.47841)
BEDtools (v2.28.0)
BLASTN (BLAST+ v2.9.0)
BWA-MEM (v0.7.17-r1188)
BlastClust (BLAST v2.2.26)
Canu (v1.6-132-gf9284f8)
Centurion (v0.1.0-3-g985439c)
Circos (v0.69-6)
DBG2OLC (commit 1f7e752)
deepTools (v3.3.0)
FigTree (commit 901211e, https://github.com/rambaut/figtree)
FreeBayes (v1.1.0-54-g49413aa)
HiRise (Dovetail Genomics proprietary software)
ILEC (map4cns commit dd89f52, https://bitbucket.org/rokhsar-lab/map4cns)
Integrated Gene Call (IGC v5)
JoinMap (v4.1)

JuiceBox (v1.9.0 and v1.11.08)
JuicerTools (commits d3ee11b and 94ec691)
Juicer (commits d3ee11b and 94ec691)
MACS (v2.2.7.1)
MAFFT (v7.427)
MEGA7 (v7.0.26)
MUMmer (v3.23)
Meraculous (v2.2.4)
Metamorph (v7.0)
OrthoVenn2 (https://orthovenn2.bioinfotoolkits.net)
PBEC (map4cns commit dd89f52, https://bitbucket.org/rokhsar-lab/map4cns)
PBJelly (PBSuite v15.8.24)
Pilon (v1.23)
Platanus (v1.2.1)
RAxML (v8.2.11)
ROAST/MULTIZ (v012109)
RepeatMasker (v4.0.7 and v4.0.9)
RepeatModeler (v1.0.11)
R (3.5.0)
SAMtools (v1.9-93-g0ca96a4)
SSPACE (v3.0)
Scaff10X (v2.1, https://sourceforge.net/projects/phusion2/files/scaff10x)
Supernova (v1.1.5 and v2.0.1)
Tandem Repeats Finder (v4.09)
TimeTree (http://www.timetree.org)
Trimmomatic (v0.39)
Trinity (v2.5.1)
alignment_plots.py (v1.0, https://github.com/abmudd/Assembly)
align_pipeline.sh (v1.0, https://github.com/abmudd/Assembly)
cactus (commit e4d0859)
cactus_filter.py (v1.0, https://github.com/abmudd/Assembly)
hic-analysis.R (v1.0, https://bitbucket.org/rokhsar-lab/xentr10)
jcvi.graphics.karyotype (v0.8.12, https://github.com/tanghaibao/jcvi)
last (v979)
minimap2 (v2.17-r941)
multiBamSummary (deeptools v3.3.0)
pbGapLen (v0.0.2, https://bitbucket.org/rokhsar-lab/xentr10)
quickmerge (commit e4ea490)
snvrate (wgs-analysis v2.0, https://bitbucket.org/rokhsar-lab/wgs-analysis)

The more detailed Supplementary Notes sections describe the use of the following additional tools:

4Dextract.py (v1.0, https://github.com/abmudd/Assembly)
Arrow (smrtlink v5.0.0.6792)
BLASR (v5.3 and commit 4323a52)
BLASTN (BLAST+ v2.3.0, v2.6.0)
BUSCO (v3.0.2-11-g1554283)
BWA (v0.7.15-r1140)
BeforePhylo (commit 0885849, https://github.com/qiyunzhu/BeforePhylo)
FreeBayes (commit 49413aa)
GMAP (version 2019-03-15)
GenomeScope (v1.0.0-6-gd2aefdd and v2.0)
IGV (v2.7.2)
Jellyfish (v2.1.4 and 2.2.0)
Newick utilities (v1.6)
NOVOPlasty (v2.6.3)
NxTrim (commit 53c2193)
PBDAGCON (v0.3 and commit 1a2f1e7)
PEAR (v0.9.8)
SAMtools (v1.6)
STARalign.sh (v1.0, https://github.com/abmudd/Assembly)
STARlong (v2.7.0e and v2.7.1a)
STAR (v2.5.3a, v2.7.0e, and v2.7.0f)
SeqKit (v0.7.2-dev)
TransDecoder (v3.0.1)
WOMBAT (v0.1.11, https://gitlab.com/Bredeson/wombat)
assembly-stats (commit 506a640, https://github.com/sanger-pathogens/assembly-stats)
assembly-patch-finder (v0.1.0, https://bitbucket.org/bredeson/artisanal)
assembly-patch-patcher (v0.1.0, https://bitbucket.org/bredeson/artisanal)
call-compartments.R (v0.1.0, https://bitbucket.org/bredeson/artisanal)
cluster-collinear-bedpe (v0.0.1, https://bitbucket.org/bredeson/artisanal)
ea-utils fastq-mcf (commit bd148d4)
exonerate (v2.4.0)
expand-gaps.py (v0.1.0, https://bitbucket.org/bredeson/artisanal)
extract2speciesmaf.py (v1.0, https://github.com/abmudd/Assembly)

filter_trinity.py (v1.0, https://github.com/abmudd/Assembly)
gbs-analysis (commit 80613d5, https://bitbucket.org/rokhsar-lab/gbs-analysis)
tsvtk (commit 80613d5, https://bitbucket.org/rokhsar-lab/gbs-analysis)
maptk (commit 80613d5, https://bitbucket.org/rokhsar-lab/gbs-analysis)
general_decon.sh (v1.0, https://github.com/abmudd/Assembly)
gff3ToGenePred and genePredToProt (KentTools accessed March 5, 2019)
largestgenePred.py (v1.0, https://github.com/abmudd/Assembly)
map4cns (commit dd89f52, https://bitbucket.org/rokhsar-lab/map4cns)
mcscan_convert_links.py (v1.0, https://github.com/abmudd/Assembly)
mcscan_invert_chr.py (v1.0, https://github.com/abmudd/Assembly)
minimap2 (v2.5-284-g1739a26)
mpGapLen (v0.1.0, https://bitbucket.org/bredeson/artisanal)
mt_decon.sh (v1.0, https://github.com/abmudd/Assembly)
nt_decon.sh (v1.0, https://github.com/abmudd/Assembly)
nxtrim_pipeline.sh (v1.0, https://github.com/abmudd/Assembly)
organelle_pipeline.py (v1.0, https://github.com/abmudd/Assembly)
redund-contigs (v0.1.0, https://bitbucket.org/bredeson/artisanal)
repair (v0.1.0, https://bitbucket.org/bredeson/artisanal)
scaffold-read-filter.py (v0.1.0, https://bitbucket.org/bredeson/artisanal)
seqtk (v1.3-r106, https://github.com/lh3/seqtk)
trim_10X.py (v1.0, https://github.com/abmudd/Assembly)

For manuscripts utilizing custom algorithms or software that are central to the research but not yet described in published literature, software must be made available to editors and reviewers. We strongly encourage code deposition in a community repository (e.g. GitHub). See the Nature Portfolio guidelines for submitting code & software for further information.

Data

Policy information about availability of data

All manuscripts must include a data availability statement. This statement should provide the following information, where applicable:

- Accession codes, unique identifiers, or web links for publicly available datasets
- A description of any restrictions on data availability
- For clinical datasets or third party data, please ensure that the statement adheres to our policy

A reporting summary for this article is available as a Supplementary Information file. Additional data supporting the findings of this work are available throughout the main text, Methods, Supplementary Information, Supplementary Data, or archived in Zenodo (https://doi.org/10.5281/zenodo.8393403). Source Data files are provided with this manuscript. All newly generated assemblies, annotations, and raw data are deposited in the NCBI GenBank and SRA databases: X. tropicalis under BioProject accession codes PRJNA577946 [https://www.ncbi.nlm.nih.gov/bioproject/PRJNA577946] and PRJNA526297 [https://www.ncbi.nlm.nih.gov/sra/?term=PRJNA526297], E. coqui under BioProject accession code PRJNA578591 [https://www.ncbi.nlm.nih.gov/bioproject/PRJNA578591], E. pustulosus under BioProject accession code PRJNA578590 [https://www.ncbi.nlm.nih.gov/bioproject/PRJNA578590], and H. boettgeri under BioProject accession code PRJNA578589 [https://www.ncbi.nlm.nih.gov/bioproject/PRJNA578589]. L. ailaonicum and P. adspersus re-assemblies were deposited at NCBI GenBank under accession DAJOPU000000000 [https://www.ncbi.nlm.nih.gov/nuccore/DAJOPU000000000] and DYDO00000000 [https://www.ncbi.nlm.nih.gov/nuccore/DYDO00000000], respectively; the versions described in this manuscript are DAJOPU010000000 [https://www.ncbi.nlm.nih.gov/nuccore/DAJOPU000000000.1] and DYDO01000000 [https://www.ncbi.nlm.nih.gov/nuccore/DYDO00000000.1]. Raw X. tropicalis ChIP-seq data are available at the NCBI SRA under BioProject accession code PRJNA726269 [https://www.ncbi.nlm.nih.gov/bioproject/PRJNA726269] and the processed data via the NCBI GEO database under series accession GSE199671 [https://www.ncbi.nlm.nih.gov/geo/query/acc.cgi?acc=GSE199671]. The E. coqui tail fin RNA-seq data generated in this study have been deposited in the NCBI SRA database under accession code PRJNA1022815 [https://www.ncbi.nlm.nih.gov/sra/?term=PRJNA1022815]. The E. coqui hindlimb developmental series RNA-seq data are available under restricted access as the project is not yet published, access can be obtained by contacting Mara Laslo at ml125@wellesley.edu.

Research involving human participants, their data, or biological material

Policy information about studies with human participants or human data. See also policy information about sex, gender (identity/presentation), and sexual orientation and race, ethnicity and racism.

Reporting on sex and gender	This manuscript describes no human subjects or biological material.
Reporting on race, ethnicity, or other socially relevant groupings	This manuscript describes no human subjects or biological material.
Population characteristics	This manuscript describes no human subjects or biological material.
Recruitment	This manuscript describes no human subjects or biological material.
Ethics oversight	This manuscript describes no human subjects or biological material.

Note that full information on the approval of the study protocol must also be provided in the manuscript.

Field-specific reporting

Please select the one below that is the best fit for your research. If you are not sure, read the appropriate sections before making your selection.

☒ Life sciences ☐ Behavioural & social sciences ☐ Ecological, evolutionary & environmental sciences

For a reference copy of the document with all sections, see nature.com/documents/nr-reporting-summary-flat.pdf

Life sciences study design

All studies must disclose on these points even when the disclosure is negative.

Sample size	No sample sizes were predetermined.<br><br>The sample size of 75 metaphase chromosome spreads for Hymenochirus boettgeri was determined by the limited number of tadpoles (n=10) available to collect material from and the amount of material required for the preparation of each spread. It is standard in the field to examine between 2 and 10 metaphase plates with well-spread chromosomes to ensure that no chromosomes were uncounted, due to overlap or loss during preparation.<br><br>Three technical replicates for ChIP-seq experiments is standard for the field.
Data exclusions	No exclusion criteria were predetermined. No data were excluded.
Replication	Metaphase chromosome spreads for Hymenochirus boettgeri were successfully reproduced for all 10 tadpoles (75 total spreads). The mean and mode of the distribution of counts centered on 2n=18 chromosomes (mean ± SE = 18.09 ± 0.22) with a frequency of 28 / 75.<br><br>Three ChIP-seq technical replicates were performed and sequenced. Samples H3 from replicate two and Ig from replicate three did not yield any broad peaks when analyzed with MACS.
Randomization	No randomization was performed. Data collection did not involve behavioral studies or case-control trials.
Blinding	No blinding was performed. Data collection did not involve behavioral studies or case-control trials.

Reporting for specific materials, systems and methods

We require information from authors about some types of materials, experimental systems and methods used in many studies. Here, indicate whether each material, system or method listed is relevant to your study. If you are not sure if a list item applies to your research, read the appropriate section before selecting a response.

Materials & experimental systems

n/a	Involved in the study	
☐	☒	Antibodies
☐	☒	Eukaryotic cell lines
☒	☐	Palaeontology and archaeology
☐	☒	Animals and other organisms
☒	☐	Clinical data
☒	☐	Dual use research of concern
☒	☐	Plants

Methods

n/a	Involved in the study	
☐	☒	ChIP-seq
☒	☐	Flow cytometry
☒	☐	MRI-based neuroimaging

Antibodies

Antibodies used	50 ug/uL final concentration of either:<br>- Rabbit anti-Xenopus laevis CENP-A (230 ng/uL stock), custom production and affinity purification against the immunizing antigen in the Straight laboratory; or<br>- Rabbit anti-Histone H4, Abcam, ab7311 (0.5 mg/mL stock); or<br>- Rabbit anti-Histone H3 Abcam, ab1791 (0.5 mg/mL stock)<br><br>50 ug/uL final concentration of:<br>- Rabbit IgG, whole molecule, Jackson ImmunoResearch, 011-000-003 (20 mg/mL stock)
Validation	Custom antibodies were affinity purified against the antigen and validated by western blotting, immunofluorescence, and immunoprecipitation using Xenopus egg extracts and cultured cells.<br><br>Commercial antibodies were validated by the manufacturers as described on the product information sheets.

Eukaryotic cell lines

Policy information about cell lines and Sex and Gender in Research

Cell line source(s)	XTN-6 cell line was generated from dissociated stage ~20 embryos of NXR Xenopus tropicalis Nigerian strain (NXR_1018, RRID:SCR_013731), sex unknown.
Authentication	None of the cell lines were authenticated.
Mycoplasma contamination	Cell lines were not tested for mycoplasma contamination.
Commonly misidentified lines (See ICLAC register)	No commonly misidentified lines were used in this study.

Animals and other research organisms

Policy information about studies involving animals; ARRIVE guidelines recommended for reporting animal research, and Sex and Gender in Research

Laboratory animals	Xenopus tropicalis was obtained from Nasco, highly inbred Nigerian and Ivory Coast strains were obtained from Mustafa Khokha (Yale IACUC Protocol #: 2021-11035). Animals were maintained by the office of Lab Animal care at UC Berkeley, under continuing IACUC AUP-2017-12-10556-1 Richard Harland)<br>Eleiutherodactyla coqui (The invasive Hawaiian coqui derived from the Puerto Rican population) HN-11 and NH-13 were adults caught by hand in the wild from the big island by Rick Elinson and transferred to Daniel Buchholz University of Cincinnati IACUC approval 06-10-03-01. Frozen Tissues from euthanized adult animals of unknown sex were provided from the animals.<br>Engystomps pustulosus (Marcos Gridi-Papp) The animals were raised under protocol #16R06 approved by the IACUC Committee of the University of the Pacific, and euthanized to provide frozen tissues from male and female adults and from late stage tadpoles.<br>Hymenochirus boettgeri: 2 adult females purchased from Albany Aquarium. These became lab animals when they entered the UC Berkelely Animal facility maintained by OLAC, under AUP (protocol ID AUP-2014-08-6596-2) to Rebecca Heald.
Wild animals	Only applicable for the invasive Hawaiian coqui (derived from the Puerto Rican population). HN-11 and NH-13 were adults caught by hand in the wild from the big island by Rick Elinson (retired). Animals were shipped overnight from Hawaii in breathable, rigid containers with sponges to maintain hydration. The animals were euthanized via overdose of anesthetic tricaine by immersion (0.2% w/v in PBS) at the end of their reproductive life span when they no longer produce egg clutches.
Reporting on sex	This study makes no claims dependent on sex, and the findings reported in this study are not influenced by the sex of the sampled frog species. All analyses are conducted either at the species level or in X. tropicalis only, which possesses no sex-specific chromosomes.
Field-collected samples	Maintained as described by Elinson RP, del Pino EM, Townsend DS, Cuesta FC, Eichhorn P. 1990. A practical guide to the developmental biology of terrestrial-breeding frogs. Biol Bull 179: 163–177. Male / female pairs were fed twice weekly with calcium-dusted crickets and kept at 25C in 40cm 23cm 29cm plastic containers with mesh lids cleaned as needed and contained a potted plant, water bowl, pvc pipe for egg laying. The animals were euthanized via overdose of anesthetic tricaine by immersion (0.2% w/v in PBS) at the end of their reproductive life span when they no longer produce egg clutches.
Ethics oversight	This study complies with the ethical standards set forth by the Institutional Animal Care and Use Committee (IACUC) protocols at the University of California, Berkeley, Yale University, University of Cincinnati, and the University of the Pacific. The IACUC and associated facilities are subject to review and oversight by NIH's Office of Lab Animal Welfare.

Note that full information on the approval of the study protocol must also be provided in the manuscript.

Plants

Seed stocks	This study uses no plant materials.
Novel plant genotypes	This study uses no plant materials.
Authentication	This study uses no plant materials.

ChIP-seq

Data deposition

☒ Confirm that both raw and final processed data have been deposited in a public database such as GEO.

☒ Confirm that you have deposited or provided access to graph files (e.g. BED files) for the called peaks.

Data access links<br>*May remain private before publication.*	https://www.ncbi.nlm.nih.gov/sra?linkname=bioproject_sra_all&from_uid=726269<br>https://www.ncbi.nlm.nih.gov/geo/query/acc.cgi?acc=GSE199671<br>https://www.ncbi.nlm.nih.gov/gds/?term=GSE199671[ACCN]%20AND%20gsm[ETYP]

Files in database submission	GSE199671_RAW.tar<br>GSE199671_readCounts_ALL.tab.gz
Genome browser session (e.g. UCSC)	https://genome.ucsc.edu/s/bredeson/XtrCR%20ChIPseq

Methodology

Replicates	Three ChIP-seq technical replicates were performed and sequenced. Samples H3 from replicate two and Ig from replicate three did not yield any broad peaks when analyzed with MACS.
Sequencing depth	For each replicate paired end 150bp Illumina sequencing was performed. Replicates ranged from 8M reads to 137M reads, with an average of 47M total number of reads
Antibodies	For ChIP-seq, antibodies include Rb-anti-Xl Cenp-a, Rb-anti-H4 (Abcam cat# 7311), and Rb-anti-H3 (Abcam cat# 1791). Rabbit IgG antibody (Jackson ImmunoResearch cat#011-000-003).
Peak calling parameters	trimmomatic PE XtGAF1-AGTCAA_S1_L004_R1_001.fastq.gz XtGAF1-AGTCAA_S1_L004_R2_001.fastq.gz -baseout XtGAF1 -phred33 -trimlog XtGAF1-AGTCAA_S1_L004_R1_001.PE.log -summary XtGAF1-AGTCAA_S1_L004_R1_001.PE.sum ILLUMINACLIP:NexteraPE-PE.fa:2:30:10 LEADING:3 TRAILING:3 SLIDINGWINDOW:4:15 MINLEN:36 -threads 8 && mv XtGAF1_1P XtGAF1_1P.fq && mv XtGAF1_2P XtGAF1_2P.fq<br><br>minimap2 -ax sr -t 8 XENTRv10-1.fasta XtGAF1_1P.fq XtGAF1_2P.fq | samtools sort -o XtGAF1-PE_sorted.bam && samtools index XtGAF1-PE_sorted.bam<br><br>multiBamSummary bins -b *PE_sorted.bam --minMappingQuality 0 --labels CenpA H4 S2 -out readCounts.npz --outRawCounts readCounts.tab<br><br>multiBamSummary bins -b *PE_sorted.bam --minMappingQuality 0 -out readCounts_ALL.npz --outRawCounts readCounts_ALL.tab<br><br>macs2 callpeak -t XtGAF3-PE_sorted.bam -c XtGAF1-PE_sorted.bam --broad -f BAM -g 1.44e+9 -n H3_vs_input-rep2 --outdir macs2_out
Data quality	Reads were trimmed with Trimmomatic v0.39 filtering for universal Illumina primers and for Nextera-PE indices. Processed PE reads were mapped with minimap2 v2.17-r941. Read counts (MapQ0) per 10-kb bin (non-overlapping) for all samples were calculated with multiBamSummary from deeptools v3.3.0. Read counts were normalized by the total number of counts in the chromosomes per sample.<br><br>We detected 60 of 1324 total peaks (4.8%), 38/298 (13%) and 2/181 (1.16%) peaks that had a FDR < 5% and a signal level above 5-fold enrichment. The vast majority of these significant peaks overlap the regions with centromere-associated tandem repeat.
Software	Trimmomatic (v0.39), minimap2 (v2.17-r941), deepTools (v3.3.0), MACS (v2.2.7.1)
